# Reducing stillbirths: screening and monitoring during pregnancy and labour

**DOI:** 10.1186/1471-2393-9-S1-S5

**Published:** 2009-05-07

**Authors:** Rachel A Haws, Mohammad Yawar Yakoob, Tanya Soomro, Esme V Menezes, Gary L Darmstadt, Zulfiqar A Bhutta

**Affiliations:** 1 Department of International Health, Bloomberg School of Public Health, Johns Hopkins University, Baltimore, Maryland, USA; 2 Division of Maternal and Child Health, the Aga Khan University, Karachi, Pakistan

## Abstract

**Background:**

Screening and monitoring in pregnancy are strategies used by healthcare providers to identify high-risk pregnancies so that they can provide more targeted and appropriate treatment and follow-up care, and to monitor fetal well-being in both low- and high-risk pregnancies. The use of many of these techniques is controversial and their ability to detect fetal compromise often unknown. Theoretically, appropriate management of maternal and fetal risk factors and complications that are detected in pregnancy and labour could prevent a large proportion of the world's 3.2 million estimated annual stillbirths, as well as minimise maternal and neonatal morbidity and mortality.

**Methods:**

The fourth in a series of papers assessing the evidence base for prevention of stillbirths, this paper reviews available published evidence for the impact of 14 screening and monitoring interventions in pregnancy on stillbirth, including identification and management of high-risk pregnancies, advanced monitoring techniques, and monitoring of labour. Using broad and specific strategies to search PubMed and the Cochrane Library, we identified 221 relevant reviews and studies testing screening and monitoring interventions during the antenatal and intrapartum periods and reporting stillbirth or perinatal mortality as an outcome.

**Results:**

We found a dearth of rigorous evidence of direct impact of any of these screening procedures and interventions on stillbirth incidence. Observational studies testing some interventions, including fetal movement monitoring and Doppler monitoring, showed some evidence of impact on stillbirths in selected high-risk populations, but require larger rigourous trials to confirm impact. Other interventions, such as amniotic fluid assessment for oligohydramnios, appear predictive of stillbirth risk, but studies are lacking which assess the impact on perinatal mortality of subsequent intervention based on test findings. Few rigorous studies of cardiotocography have reported stillbirth outcomes, but steep declines in stillbirth rates have been observed in high-income settings such as the U.S., where cardiotocography is used in conjunction with Caesarean section for fetal distress.

**Conclusion:**

There are numerous research gaps and large, adequately controlled trials are still needed for most of the interventions we considered. The impact of monitoring interventions on stillbirth relies on use of effective and timely intervention should problems be detected. Numerous studies indicated that positive tests were associated with increased perinatal mortality, but while some tests had good sensitivity in detecting distress, false-positive rates were high for most tests, and questions remain about optimal timing, frequency, and implications of testing. Few studies included assessments of impact of subsequent intervention needed before recommending particular monitoring strategies as a means to decrease stillbirth incidence. In high-income countries such as the US, observational evidence suggests that widespread use of cardiotocography with Caesarean section for fetal distress has led to significant declines in stillbirth rates. Efforts to increase availability of Caesarean section in low-/middle-income countries should be coupled with intrapartum monitoring technologies where resources and provider skills permit.

## Introduction

Although most pregnancies progress normally, some are more complex because of antenatal or intrapartum conditions that place the mother, the developing fetus, or both at a higher risk for complications than pregnancies without these conditions. Pre-existing chronic conditions, as well as conditions that arise during pregnancy, can threaten the life and health of the fetus or the mother. Maternal hypertension, diabetes mellitus, renal disease, and autoimmune disorders, as well as placentation abnormalities and congenital anomalies, are examples of conditions that can place the pregnancy at high risk of fetal compromise. Fetal growth restriction arising from placental insufficiency is a significant cause of perinatal mortality (stillbirth or neonatal death) and morbidity (complications of prematurity) internationally [1]. Additionally, if not detected and addressed promptly, fetal hypoxia resulting from placental dysfunction or poor fetal tolerance of labour can cause stillbirth, neonatal death, or physical and developmental disabilities in the child [2].

Relatively non-invasive techniques exist to screen for a number of these conditions during the antenatal and intrapartum periods. These screening tools can also be used to monitor fetal well-being via assessment of fetal movement, heart rate, and/or growth; and feto-placental and/or uteroplacental circulatory dynamics, whether routinely at antenatal care (ANC) visits or via more complex screening tests in high-risk and post-term pregnancies [3]. Despite widespread clinical use of many of these techniques, the sensitivity and predictive value of these tests and methods are often too poor to reliably detect problems. Prompt detection of risk factors and complications is also critical, as measures of fetal distress or compromise associated with certain high-risk conditions may rapidly lead to fetal demise. Certain maternal or fetal problems may prompt the need for pharmacological intervention, early delivery, or surgical delivery (Caesarean section) rather than vaginal delivery. Optimising gestational age at delivery and judicious timing of corticosteroid administration are key challenges in responding to fetal compromise arising pre-term. The appropriate use of accurate screening and monitoring technologies can facilitate timely referral to facilities capable of providing operative delivery or other interventions for complications prior to or during labour. On the other hand, screening and monitoring techniques during pregnancy and the intrapartum period could inadvertently result in avoidable perinatal deaths, either because the technique itself is harmful or because it increases the risk of inappropriate or unnecessary use of drugs, induction of labour, early delivery, or Caesarean section.

Most studies of fetal screening and monitoring to date have been conducted in high-resource settings. Theoretically, evidence-based screening and monitoring techniques that are already in widespread use in high-income countries could be promoted to prevent stillbirth and other adverse pregnancy outcomes in low-/middle-income countries. We focus here on monitoring methods during pregnancy and the intrapartum periods, including identification and care of high-risk pregnancies and advanced monitoring techniques, with attention given where relevant to the feasibility and potential impact of implementing these techniques in low-resource settings where most stillbirths occur.

## Methods

This is the fourth in a series of papers on the evidence for interventions that impact stillbirths. Details of the search strategy and review procedures for this paper are described in detail in Paper 1 of this series [4]. Each study was assigned a level of evidence (LOE) based on its design strength, size, and findings. The cumulative strength of the body of evidence for each intervention was then graded as A, B, C, or D using the SIGN grading system; impact estimates for each intervention were further cumulatively assessed as having no/negative, uncertain, some or clear evidence of benefit.

We reviewed 14 screening and monitoring interventions for evidence of impact which are included in this paper (Table 1). For most of these interventions, we first reviewed studies reporting how effectively a given screening or monitoring test detected potential risk to the fetus (primarily observational studies), followed by studies that assessed the utility and/or impact of screening or monitoring interventions in preventing adverse outcomes, for which randomised controlled trials (RCTs) were most informative.

**Table 1:** Screening and monitoring interventions reviewed in this paper

***Identification and care of high-risk pregnancies***
Pregnancy risk screening
Fetal movement counting
Routine ultrasound scanning
Doppler velocimetry
Pelvimetry
Detection and management of maternal diabetes mellitus

***Advanced monitoring in pregnancy ***

Antenatal fetal heart rate monitoring using cardiotocography
Fetal biophysical profile test scoring
Vibroacoustic stimulation
Amniotic fluid volume assessment
Home versus hospital-based bed rest and monitoring in high-risk pregnancy
In-hospital fetal surveillance unit

***Monitoring during the intrapartum period***

Use of the partograph
Cardiotocography with or without pulse oximetry

## Results

### Identification and care of high-risk pregnancies

#### Pregnancy risk screening

##### Background

Early identification of high-risk pregnancies can theoretically facilitate monitoring, referral and prompt initiation of therapy. Multiple screening and scoring systems have been developed to assess obstetric risk generally [5,6], as well as the risk of preterm labour, Caesarean delivery, and other maternal and fetal outcomes. Risk scores using these systems can range from simple additive scores to the products of more complex multivariable models that quantify risk factors according to their association with adverse outcomes [7]. An effective risk screening system, particularly if convenient to implement and relatively non-dependent on diagnostic technologies, would be particularly useful in low-resource settings to help providers identify high-risk pregnancies and refer them for appropriate facility-based care, and to help facilities allocate scarce resources.

##### Literature-based evidence

Ten observational studies met our inclusion criteria; none tested interventions for pregnancies scored as high-risk (Table 2). Most risk scoring systems were originally developed and tested in high-resource settings. At a national hospital in New Zealand, Pattison et al. [8] developed and tested an antepartum risk scoring system (N = 29,101 consecutive pregnancies) using prior obstetric history and current pregnancy risk factors, where a fetal risk score ≥ 3 denoted high risk. One-third of the total population (N = 10,859) was scored as high-risk, and 90% of those who had a perinatal death were identified using the scoring system. Women with an antepartum risk score of 7 or more (very high risk) had a perinatal mortality rate of 200/1000, whereas the low risk group of 18,242 (63%) had a perinatal mortality rate of 4.1/1000. The system clearly identified the population at risk of fetal or early neonatal loss, but could not effectively predict the need for intervention, as 60% of the low-risk group had a complicated pregnancy requiring intervention ***[LOE: 2-]***. The same research group later used this dataset to develop a statistically derived antenatal risk scoring system using data on 27 antenatal variables from 20,985 pregnancies [9]. Tested on 3120 subsequent pregnancies, the scoring system had a positive predictive value of 0.73 in early pregnancy and 0.91 at onset of labour. Although only 16% of pregnancies were classified as high-risk at onset of labour, 87% of adverse outcomes occurred within this group. The positive predictive value of this system was higher than any previously reported statistically derived score, but requires that clinicians be able to sum logistic coefficients (basic statistical analysis), which requires more training than some other systems ***[LOE: 2-]***.

**Table 2:** Impact of pregnancy risk screening on stillbirth and perinatal mortality

**Source**	**Location and Type of Study**	**Intervention**	**Stillbirths/Perinatal Outcomes**
***Observational studies***			

Abraham et al. 1991 [18]	India. Health centre setting. Prospective cohort study. Health workers at 6 primary health centres used a home-based mothers card with pregnant, mostly illiterate women (N = 2446).	Assessed the association of perinatal mortality with risk factors recorded on a home-based mother's card to pregnant women on which risk factors and ANC attendance were documented.	PMR directly related to # of risk factors: 0 risk factors: PMR = 25.9/1000 1 risk factor: PMR = 39.7/1000 2 risk factors: PMR = 56.5/1000 3 risk factors: PMR 122.5/1000)

Chard et al. 1992 [10]	UK. N = 994 pregnant women (470 primiparae; 524 multiparae)	Used receiver-operating characteristic curves (ROC) to compare the use of weighted and unweighted risk scores in estimating an overall risk score based on individual risk factors, and relating this score to fetal outcome.	Weighted risk factor method clearly superior to unweighted risk factor method in primiparae. No difference in multiparae.

Cho et al. 1991 [163]	Korea. Chung Ang Medical Center. Cross-sectional study to test scoring system. N = 1300 pregnant women (N = 1313 infants) admitted from 1988–1990.	Assessed the utility of Edwards' scoring system adapted to a Korean setting in identifying high-risk pregnancy. Risk scoring included demographic, obstetric, medical, and miscellaneous factors.	560 infants (42.7%) were born to mothers with risk-scores greater than 7, and 753 infants (57.3%) were born to mothers with risk-scores less than 7.

Lefevre et al. 1989 [15]	USA. Rural primary care setting. Prospective study. N = 635 women. N = 47 (8.3%) adverse outcomes.	Tested the predictive value of Coopland's obstetric risk in anticipating adverse outcome (perinatal death, birthweight < 2500 g, 5-min Apgar score < 7, or newborn transferred to a level 2 or level 3 nursery.	There was a clear relationship between risk score and probability of adverse outcome. Good sensitivity could be achieved only at the expense of a very high false-positive rate, however. Risk scoring no more effective than a policy that would refer all women with standard obstetric risk factors; majority of adverse outcomes occurred in women identified as low-risk.

Majoko et al. 2002 [12]	Zimbabwe. Rural setting. Evaluation of screening test; sub-study of ANC trial. N = 5223 women who received traditional care from nurse-midwives in 12 rural health centres (N = 2890 high risk).	Used traditional risk scoring at ANC booking to group women into low- and high-risk groups. High-risk women were encouraged to deliver in facilities.	Complications: 924 (17.7%) of women; 62.4% had had risk markers identified at booking. 20% (577/2890) of women classified as high risk developed complications. Predictive ability of risk allocation: Likelihood ratio = 1.16.

Mikulandra 1986 [164]	Croatia. Prospective study.	Assessed the associations of a risk factor scale (low, moderate, and high risk) for pregnancy and delivery on perinatal outcomes. High pregnancy risk: 10.9% of cases. High intrapartum risk: 14.02% of cases.	Severe asphyxia (Apgar ≤3): 0.37%, 0.81%, and 4.36% in low, moderate, and high-risk groups, respectively (P < 0.001). SBR: 0.76% vs. 34.48% in low vs. high-risk groups (P < 0.01)

Morrison 1980 [165]	USA. Retrospective analysis. N = 1994 consecutive parturients, N = 472 (23%) high-risk (risk score ≥ 3).	Assessed the association of high-risk (risk score ≥ 3) pregnancy with adverse perinatal outcomes.	PMR: Significantly higher in high-risk group (P > 0.001). Abnormal intrapartum outcome: 71% of high-risk group (P < 0.0001).

Morrison 1979 [11]	USA. N = 16,733 deliveries. Women scored during pregnancy using a simplified, numerical form for antepartum risk scoring.	Tested the predictive value of a simplified risk scoring system in anticipating the risk of perinatal mortality.	19% of group was high-risk (score ≥ 3). PMR: 69/1000 vs. 7/1000 in high- vs. low-risk groups, respectively (P < 0.0001). 70% of perinatal deaths occurred in high-risk group.

Talsania et al. 1994 [14]	India (Ahmedabad). N = 687 indigent women enrolled during first trimester. Women scored as no, mild, moderate, or severe risk based on sociodemographic and obstetric data.	Assessed association of risk factors and risk scoring with perinatal mortality.	PMR: 84.77/1000 births overall; 7.94 in no risk, 92.20/1000 for mild, 200/1000 for severe. Statistically significant. PMR: OR = 13.09 in women with risk factors vs. women without, respectively. PM sensitivity, specificity, PPV were 98.31%, 19.90%, and 10.34% respectively.

Talsania et al. 1991 [13]	India (Ahmedabad). N = 687 women enrolled at < 12 wks gestation, given risk scoring during their first and second visits, during their second and third trimesters, and when admitted for delivery.	Assessed the association of risk factors with perinatal mortality.	81.66% had risk factors. Women with no risk factors had no stillbirths, while 20% of those in the highest risk group did.

In the UK, an effort by Chard et al. [10] to calculate obstetric risk scores from individual risk factors (N = 2029 pregnant women) found that risk scores were useful only for identifying the small group of women at particularly high risk of adverse fetal outcomes. For most women, risk scores were uninformative ***[LOE: 2-]***.

In the USA, Morrison et al. [11] found that perinatal mortality was significantly higher in the high- versus the low-risk groups identified with the application of a simplified risk scoring system, where high risk was a score of 3 or greater (69/1000 versus 7/1000, respectively, P < 0.0001). Seventy percent of perinatal deaths occurred in the high-risk group, which was 19% of the total group screened ***[LOE: 2-]***.

Other studies implemented risk scoring systems in more remote or low-resource settings in low-/middle-income countries. Attempting to predict intrapartum complications in rural Zimbabwe where most women receive care from nurse-midwives, Majoko et al. [12] employed antenatal risk assessment at the first antenatal visit based on medical and demographic measures and obstetric history (N = 5223 women at 12 health centres). All high-risk women (N = 2890) were encouraged to seek hospital delivery. Of the 924 (17.7%) women who experienced complications, 577 (62.4%) had had risk markers identified at booking; however, only 20% (577/2890) classified as high risk developed intrapartum complications. This risk screening system had a likelihood ratio of 1.16, indicating it was ineffective in identifying women at risk of pregnancy complications and generated too large a risk group for referral ***[LOE: 2-]***.

In India, Talsania et al. [13,14] reported on the application of an antenatal risk scoring system (N = 687 women), and observed that no stillbirths occurred among women with no identified risk factors, whereas among women in the highest risk group, 20% had stillbirths. Perinatal mortality was 84.77/1000 births, and 7.94 among the no risk group. The perinatal mortality rate rose with level of risk, with a rate of 92.20/1000 births for the women with mild risks to a rate of 200/1000 for those with severe risks, which was statistically significant. Sensitivity, specificity, and positive predictive values for perinatal mortality were 98.3%, 19.9%, and 10.3%, respectively ***[LOE: 1+]***.

In a rural primary care setting in the USA, Lefevre et al. [15] found a clear relationship between risk score and probability of adverse outcome, but cautioned that good sensitivity could be achieved only with a very high false-positive rate, as the majority of adverse outcomes occurred in women identified as low-risk ***[LOE: 2-]***

In a remote area of Australia, Humphrey et al. [16] employed pregnancy risk scoring (N = 2875 women with singleton births), and found that during the study period, hospital and regional perinatal mortality rates fell by more than half. Women with low-risk scores had a statistically significantly lower incidence of preterm birth, leading the authors to conclude that risk scoring can be of benefit in allocating scarce resources ***[LOE: 2-]***.

Several other retrospective analyses attempted to associate perinatal mortality with the presence of specific risk factors. In Guadeloupe, West Indies, using data from the 1984–85 Guadeloupean Perinatal Audit, de Caunes et al. [17] observed that perinatal mortality was associated with a specific combination of risk factors representing maternal demographic, socioeconomic, obstetric history and risk characteristics measurable at the first antenatal visit, leading the authors to advocate for risk assessments specific to pregnancy outcomes within specific populations ***[LOE: 3-]***.

Using risk screening as a strategy to facilitate monitoring and referral, Abraham et al. [18] adapted the Home Based Mothers Card recommended by the World Health Organisation (WHO) for a rural Indian setting. Perinatal mortality was directly associated with number of risk factors: perinatal mortality rates (PMRs) were higher among women with 3 or 4 risk factors than those with 1 or 2 risk factors ***[LOE: 2-]***.

##### Conclusion

A number of studies reviewed were able to successfully identify women at high risk of obstetric complications. However, despite good sensitivity, risk scoring systems [7] have poor positive predictive value in anticipating adverse birth outcomes, particularly when used well before term or in populations significantly different from the population in which the system was developed [19]. This limitation of risk scoring systems limits the impact of their use. No studies were found that effectively incorporated risk screening with appropriate interventions to demonstrate a possible impact on stillbirth or perinatal mortality rates compared to a control group. The evidence for risk screening at the community level yielded a Grade C assessment.

#### Fetal movement counting

##### Background

Monitoring fetal movements using counting strategies is an indirect measure of central nervous system integrity and fetal responsiveness. Commonly employed in clinical practice, fetal movement counting is a simple and inexpensive means of monitoring fetal well-being [20]. The rationale for fetal movement counting is that decreased fetal movements signal decreased oxygenation, which often precedes fetal demise [1]. Kick charts or other recording strategies involve a pregnant woman in the second half of pregnancy monitoring fetal movements, documenting the frequency of movements she feels, and reporting these counts to her physician. Changes in these counts, particularly decreases, indicate possible fetal compromise, and thus alert care providers to the need for further diagnostic tests such as non-stress testing or the biophysical profile. Cessation of movement can indicate impending fetal death, while gradual diminishment of activity can indicate chronic fetal compromise [21]. Fetal movement monitoring may be used routinely, or only in high-risk pregnancies. There are many different counting methods, and fetal movement monitoring has a wide following among clinicians, who perceive the practice to serve as an early warning system for fetal compromise. A criticism of the practice is that it may cause undue worry for the pregnant woman [22].

##### Literature-based evidence

The literature search identified one Cochrane review comprised of three RCTs; and seven observational and intervention studies (Table 3).

**Table 3:** Impact of fetal movement counting on stillbirth and perinatal mortality

**Source**	**Location and Type of Study**	**Intervention**	**Stillbirths/Perinatal outcomes**
***Reviews and meta-analyses***			

Mangesi et al. 2007 [22]	Peru, Denmark. Meta-analysis (Cochrane). 3 RCTs included (N = 66 women).	Routine fetal movement counting (intervention) versus mixed or undefined fetal movement counting (controls).	SBR: weighted mean difference = 0.23 [95% confidence interval (CI): -0.61–1.07) [NS] [Mean (SD) = 2.90 (1.90) vs. 2.67 (1.55) in intervention vs. control groups, respectively].

***Intervention studies***			

Gomez et al. 2007 [166]	Peru. Hospital setting. RCT. Pregnant women (N = 1400).	Compared two different charting methods: a novel fetal movement chart proposed by the Latin American Center for Perinatology (CLAP) (intervention) vs. the count-to-ten Cardiff chart method (comparison).	Fetal death (miscarriage+SB): Relative risk (RR) not estimable. [0/700 in both groups].

Grant et al. 1989 [28]	UK, USA, Ireland, Sweden, Belgium. Cluster RCT. 66 clusters. Pregnant women (N = 68654 women; N = 31993 intervention, N = 36661 controls).	Compared the impact on birth outcomes of asking mothers to keep routine kick charts (intervention) vs. not keeping kick charts (controls).	Unexplained late antepartum fetal death: 59/31993 (2.9/1000) vs. 58/36661 (2.7/1000) in intervention vs. control groups, respectively [NS].

Moore 1989 [27]	USA. Hospital setting. Before-after pilot study (N = 2519 deliveries before intervention, N = 1864 after introduction of intervention.)	Assessed the impact of introducing formal fetal movement assessment (intervention) compared to no monitoring before the intervention (controls).	Fetal death (miscarriage+SB): 2.1/1000 vs. 8.7/1000 after vs. before, respectively. (χ^2 ^= 6.8; P < 0.01)

***Observational studies***			

De Muylder et al. 1988 [24]	Zimbabwe. Hospital setting. Prospective cohort study. High-risk pregnant women (N = 200).	Compared the obstetrical outcome among the patients with a normal kick chart (unexposed), compared to those with an abnormal count (exposed).	SBR: 19.4% vs. 0.7% in charts that went from normal to being abnormal vs. unexposed. (P < 0.001) PMR: 22.2% vs. 2.7% for previously normal charts that became abnormal vs. unexposed (P < 0.001)

Lema 1988 [23]	Kenya. Urban hospital setting. Prospective cohort study. High-risk pregnant women (N = 110).	Compared birth outcomes among women with good fetal movements vs. poor fetal movements.	SBR: 12/1000 (1/83) vs 185/1000 (5/27) in the good vs. poor fetal movements group, respectively. No statistical significance data.

Sinha et al. 2007 [25]	UK. Hospital setting. Retrospective cohort study. N = 180 case reports.	Compared the impact of reduced fetal movements (exposed) to women without reduced fetal movements (unexposed) on PMR.	PMR: RR not estimable. [0/90 in the exposed vs. 0/90 in the control groups, respectively]. Intervention needed solely due to fetal compromise: 29/90 (32%) in the study vs. 19/90 (21%) in the control groups, respectively.

Romero Gutiérrez et al. 1994 [26]	Mexico. Hospital setting. Prospective cohort study. Pregnant women (N = 200; N = 100 intervention, N = 100 controls) 32–41 wks gestation without risk factors.	Compared the impact of decreased fetal movement (exposed) vs. normal fetal movement (unexposed) on PMR.	PMR: No difference [NS]

Several trials in high-risk pregnancies documented an association between poor fetal movements and stillbirth/perinatal mortality rates. A trial among high-risk women (N = 110) by Lema [23] found that fetal movement was a predictor of stillbirth rate, documenting differential rates [12/1000 (1/83) versus 185/1000 (5/27) in the groups with good versus poor fetal movements, respectively] ***[LOE: 2-]***. De Muylder [24] evaluated the use of a kick chart to monitor the fetus in high-risk pregnancies, finding that both stillbirth rates (SBR) and PMR increased significantly if a previously normal chart kick chart became abnormal (antepartum SBR = 194/1000 versus 7/1000; and PMR = 222/1000 vs. 27/1000 in charts that became abnormal versus normal, respectively, P < 0.001) ***[LOE: 2-]***. Two other observational studies found no difference in perinatal mortality between groups with good versus poor fetal movements measured using kick charts [25,26].

Other observational studies, RCTs, and reviews assessed impact on perinatal mortality of interventions using kick charts. A before-after study by Moore et al [27] introduced formal fetal movement monitoring into clinical practice, and stillbirth rates declined from 8.7 to 2.1/1000 over the course of the study (χ^2 ^= 6.8; P < 0.01). A Cochrane review by Mangesi et al [22] included 3 trials that tested strategies of routine kick counting, but varied study designs precluded outcome pooling (Additional file 1). Unfortunately, no included trials compared fetal movement counting with no fetal movement counting, and all studies showed nonsignificant impact on perinatal outcomes, including stillbirth incidence. ***[LOE: 1++]***.

The largest RCT testing kick charts [28] assessed the impact of the use of kick charts on unexplained stillbirth in normally-formed singleton pregnancies (N = 68654), and found no difference in rates of fetal death between intervention and control groups (59/31993 [2.9/1000] versus 58/36661 [2.7/1000], respectively [NS]). These findings alone largely shaped the UK National Institute for Health and Clinical Excellence (NICE) evidence-based routine antenatal care guidelines, which do not recommend the use of kick charts in uncomplicated pregnancy [29]***[LOE: 1+]***. However, of the 17 women in the study randomised to kick charts who alerted their provider about decreased fetal movement and subsequently delivered a stillborn baby, none had an emergency delivery, as follow-up testing using cardiotocography resulted in false negatives for all 17 women[30].

##### Conclusion

The existence of a Cochrane review of multiple RCTs [22] yields a Grade B evidence rating. In keeping with the NICE guidelines based largely on 1 study [28], evidence from these studies indicates a lack of impact of fetal movement monitoring on stillbirth or perinatal mortality. Despite indirect evidence that formal movement monitoring using a counting method is more effective than mothers' subjective assessments of fetal movement in identifying babies at risk of intrauterine death, false negatives on subsequent fetal assessment tests and clinical error may be responsible for the lack of impact on perinatal mortality, as suggested by Del Mar et al [30]. Monitoring does appear to be of some value in high-risk pregnancies [23,24], particularly those in which there is suspicion of placental insufficiency. Routine fetal movement monitoring is currently recommended only for high-risk pregnancies, particularly those in which there is clinical suspicion of restricted fetal growth or placental dysfunction revealed through ultrasonographic or Doppler studies. Further studies are warranted to determine which methods of fetal movement counting prove most effective in identifying complications (sensitivity and specificity) early enough for interventions to prevent stillbirth, as well as acceptability to and feasibility for women. Universal fetal movement monitoring for all pregnancies is unsupported by scientific evidence.

#### Routine use of ultrasound scanning

##### Background

Ultrasound scans during pregnancy are widely used, even in many resource-poor settings, but availability and quality of ultrasound machines vary, and ultrasound operators in some settings may lack the ability to accurately interpret imaging. Diagnostic ultrasound examination may be employed to date pregnancies, identify multiple pregnancies, document placental location, identify fetal anomalies (particularly when the fetus is suspected to be at high risk of malformation), identify fetal growth restriction or abnormal amniotic fluid volume, or to investigate clinical complications (e.g., bleeding). Some clinicians have postulated that routine use of ultrasound in all pregnancies could identify problems in asymptomatic pregnancies, whether early or late in gestation [31].

##### Literature-based evidence

We identified two Cochrane reviews and five other intervention/observational studies; we also conducted an independent meta-analysis incorporating 9 RCTs (Table 4).

**Table 4:** Impact of use of routine ultrasound scanning on stillbirth and perinatal mortality

**Source**	**Location and Type of Study**	**Intervention**	**Stillbirths/Perinatal Outcomes**
***Reviews and meta-analyses***			

Bricker et al. 2008 [34]	New Zealand), Norway (Trondheim), Australia, UK (Peterborough), USA. Meta-analysis (Cochrane). 8 RCTs included (N = 21,708 women).	Assessed the effects of routine ultrasound > 24 wks (intervention) vs. no/concealed/selective ultrasound > 24 wks (control).	SBR: RR = 1.11 (95% CI: 0.29–4.26) [NS] [45/10894 vs. 38/10814 in intervention vs. control groups, respectively]. PMR: R = 0.94 (95% CI: 0.55-1.64) [NS] [79/12198 vs. 75/12078 in intervention vs. control groups, respectively.]

Neilson 1998 [31]	Finland, UK, USA, Sweden, Trondheim, South Africa. Meta-analysis (Cochrane). 8 RCTs included (N = 34,245).	Assessed the effects of routine ultrasound (intervention) vs. the selective use of ultrasound (control) in early pregnancy (i.e. < 24 wks).	PMR: OR = 0.86 (95% CI: 0.67–1.12)

***Intervention studies***			

van Dyk et al. 2007 [167]	South Africa. Open cluster RCT. Pregnant women (N = 804).	Compared the impact of ultrasound screening (intervention) vs. no ultrasound (controls).	PMR: RR = 1.05 (95% CI: 0.54–2.03, P = 0.88.) [NS] [18/416 (4.3%) vs. 16/388 (4.1%) in intervention vs. control groups, respectively].

***Observational studies***			

Cristina et al. 2005 [168]	Spain. Retrospective (case-control) review of all obstetric ultrasounds. Pregnant patients (N = 5,987 examined by ultrasound scan at 20 wks; N = 40 cases with a single umbilical artery, N = 82 controls).	Compared the impact of having a single umbilical artery (cases) vs. not having this condition (controls) as diagnosed by ultrasound scan on PMR.	PMR: 5% (2/40) among single uterine artery cases (10× greater than overall patient rate). No statistical significance data.

Mahran et al. 1992 [32]	Egypt. Tertiary care setting. Comparison of diagnostic tests. Pregnant women (N = 828), of whom a proportion had growth-restricted neonates (N = 98).	Compared the effectiveness of diagnostic ultrasound (intervention) vs. fundal palpation (controls) in predicting growth restriction.	Growth restriction: 89.7% (88/98) vs. 34.7% (34/98) detection rate in ultrasound vs. fundal palpation groups, respectively.

Sylvan et al. 2005 [169]	Sweden. University clinics. Observational cohort study. Deliveries from 1985–1996; stored data (N = 209,726).	Compared the impact of routine ultrasound screening (exposed group) vs. no routine screening (unexposed) in third trimester on PMR.	PMR: [NS] [160/56,371 vs. 488/153,355 in exposed vs. unexposed, respectively.]

Viero et al. 2004 [170]	Canada. Observational study. Structurally and chromosomally normal singleton pregnancies (N = 60) with abnormal fetoplacental blood flow < 32 wks of gestation; N = 21 of these resulted in stillbirth and were delivered vaginally.	To assess the ability of grayscale placental ultrasound to detect pathological lesions in the placentas of pre-term pregnancies.	SB: charts with both abnormal uterine artery Doppler and abnormal grayscale findings strongly predictive of stillbirth (17/21 SBs; sensitivity 81%, PPV 52%, P = 0.006).

One observational study of routine ultrasound use suggested that ultrasound may help to identify some high-risk pregnancies. In Egypt, Mahran et al [32] reported that routine ultrasound was superior to fundal palpation in identifying fetal growth restriction (89.7% versus 34.7% of growth-restricted infants identified accurately with each method, respectively). No observational studies reported any statistically significant impact of routine ultrasound scanning on subsequent stillbirth rates. A Cochrane review on the impact of ultrasound during pregnancy is in progress [33].

Another Cochrane review by Neilson [31] reviewed adequately controlled trials of routine ultrasound imaging in early pregnancy (N = 9) (Additional file 2). The study found that routine ultrasound examination was associated with earlier detection of multiple pregnancies and reduced rates of induction of labour for post-term pregnancy, but ultrasound had no impact on PMR [Odds ratio (OR) = 0.86, 95% confidence interval (CI): 0.67–1.12], even in twin pregnancies, despite generally earlier diagnosis in the ultrasound-screened pregnancies. Where detection of fetal abnormality was a specific aim of the examination, ultrasound was associated with increased terminations of pregnancy ***[LOE: 1+]***

A second Cochrane review by Bricker et al. [34] assessed the impact of ultrasound in late pregnancy (8 RCTs, N = 27,024 women) and found no difference in antenatal, intrapartum and neonatal intervention or morbidity in those undergoing ultrasound screening versus those not screened (Additional file 3). The Caesarean section rate was slightly higher among the screened group, but this difference did not reach statistical significance. Routine late pregnancy ultrasound was not associated with improvements in overall perinatal mortality. 

An additional RCT (N = 1528 women) from New Zealand by Duff et al. [35] documented a statistically non-significant increase in SBR among women scanned twice during pregnancy, at 16–24 weeks and again at 32–36 weeks gestation ***[LOE: 2+]***. In another RCT by Proud et al [36] where placental grading information from ultrasound screening was either given to a clinician (intervention) or withheld (controls), the antepartum SBR (excluding lethal malformations) was 0/1014 versus 9/1011 among intervention versus controls, respectively (P < 0.05) ***[LOE: 2+]***.

##### New meta-analysis

**Figure 1:**
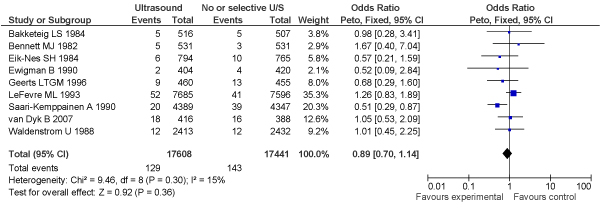
**Forest plot of results of meta-analysis of perinatal mortality rates in women examined by routine vs. selective ultrasound in early pregnancy**.

We also conducted an independent, new meta-analysis for the purposes of this review, as we identified 9 RCTs (N = 35,049 women) reporting an impact on perinatal mortality rate of ultrasound in early pregnancy versus no or selective use of ultrasound in early pregnancy (before 24 weeks) (Figure 1). We found no significant difference between the 2 groups when the results were pooled (OR = 0.89, 95% CI: 0.70–1.14).

##### Conclusion

There is no clear evidence that ultrasound examination during pregnancy is harmful [31], and its assumed benefits include (1) better gestational age assessment; (2) earlier detection of multiple pregnancies; (3) determination of placental location to rule out placenta praevia; (4) detection of clinically unsuspected fetal malformation when termination of pregnancy is more feasible, and (5) monitoring of fetal growth for pregnancies at increased risk of fetal growth restriction or macrosomia. The reduced incidence of induction of labour for apparent post-term pregnancy in the routinely scanned groups presumably results from better gestational age dating, and twin pregnancies being detected earlier. Neither of these effects has been shown to improve fetal outcome, but much larger numbers of participants would be required to accurately measure this outcome.

Based on the results of 2 Cochrane reviews, our meta-analysis, and other RCTs (overall Grade B evidence), there is no evidence that routine ultrasonography has any impact on perinatal mortality compared to the selective use of ultrasonography based on clinician judgement. It may be that routine ultrasound cannot reliably detect complications, or that high rates of false positives expose higher numbers of babies to iatrogenic intervention (particularly the risk of iatrogenic preterm birth in the event of inaccurate gestational age dating). The routine use of early ultrasonography in pregnancy cannot be recommended to prevent stillbirth, as there is no evidence of its benefit in preventing stillbirth or perinatal mortality. There is a need for decision analysis studies subsequent to diagnostic ultrasound, like the study of indicated Caesarean section for fetal macrosomia diagnosed by ultrasound by Rouse et al [37], but which report perinatal mortality outcomes. Clinics and hospitals, particularly those in resource-constrained settings, must assess whether the potential benefits are worth the cost of routine ultrasound screening for all pregnant women.

#### Doppler velocimetry

##### Background

In many high-income countries, Doppler ultrasound studies are used as a non-invasive means to assess the sufficiency of uterine and umbilical cord blood flow. These velocimetry studies can improve management of pregnancies by aiding identification of fetuses at highest risk of adverse outcomes associated with pre-eclampsia, fetal growth restriction, and congenital malformations. Management of pre-term pregnancies with signs of fetal growth restriction and pre-eclampsia is complex, especially before 32 weeks gestation. The risk of prolonged hypoxia and acidaemia leading to stillbirth or neonatal death if the pregnancy is allowed to progress must be balanced against the risks of neonatal morbidity and mortality associated with prematurity if early delivery is chosen. Doppler ultrasonographic evaluation may aid determinations of the degree to which the fetus may be or become compromised.

Early in normal pregnancy, trophoblasts invade the maternal uterine spiral arteries and reduce resistance to uterine blood flow. Impeded flow measured by uterine artery Doppler suggests a failure of this trophoblastic invasion, which is associated with subsequent pre-eclampsia, fetal growth restriction, and stillbirth [38]. Uterine artery Doppler studies could therefore be helpful in identifying women likely to develop pre-eclampsia or to have a growth-restricted fetus [39].

Observational Doppler studies of the umbilical artery, first conducted in the 1970s, consistently showed a correlation between extremely abnormal waveforms and adverse outcomes, including fetal growth restriction and stillbirth [40-42]. In growth-restricted fetuses, results of umbilical and fetal Doppler waveform analyses suggest progressive severity of fetal compromise [38,43]. Initially, umbilical artery velocity waveforms show increased resistance; subsequent deterioration is indicated by absent or even reversed end diastolic flow in the umbilical artery. Later, fetal middle cerebral artery flow shows decreased resistance, indicating brain sparing, and eventually, abnormal venous Doppler results (ductus venosus waveforms and umbilical vein pulsatility) suggest fetal cardiac dysfunction. Consequent central nervous system damage then manifests as non-reactive results to fetal tests of well-being, but there is wide variability in the timeline of fetal progression to severe compromise [38,43].

Despite these well-established markers of fetal compromise in Doppler testing, it is not clear whether abnormal results of different modalities of Doppler ultrasound lead to improved perinatal outcomes and prevention of stillbirths, nor are the most appropriate indications and timing of testing known. Additionally, if a fetus is not seriously compromised, Doppler ultrasound may potentially cause iatrogenic harm in suggesting the need for inappropriate early delivery.

##### Literature-based evidence

The literature search identified three systematic reviews, one Cochrane protocol, and 10 other intervention/observational studies (Table 5 and Table 6).

**Table 5:** Impact of uterine artery Doppler velocimetry on stillbirth and perinatal mortality

**Source**	**Location and Type of Study**	**Intervention**	**Stillbirths/Perinatal Outcomes**
***Reviews and meta-analyses***			

Papageorghiou et al. 2002 [44]	Multiple sites. Review. 15 studies of routine Doppler assessments in pregnancy in unselected populations.	Sought to relate the risk of antepartum stillbirth to uterine artery Doppler flow velocimetry at 22–24 weeks.	Fetal growth restriction and perinatal death associated with impeded uterine artery flow. Positive Doppler diagnosis appropriately identified ~40% of women who subsequently developed pre-eclampsia (6-fold increased risk with positive Doppler) and ~20% of fetal growth restriction cases (3.5-fold increased risk)

***Intervention studies***			

Subtil et al; Essai Régional Aspirine Mère-Enfant (ERASME) Collaborative Group 2003. [46]	France and Belgium. Multicentre RCT. Nulliparous women (N = 1853; N = 1253 intervention, N = 617 controls) 14–20 wks gestation.	Compared the impact of uterine Doppler (intervention) versus placebo (controls) on PMR. Women with abnormal Doppler waveforms received 100 mg of aspirin daily from Doppler exam until 36 wks.	PMR: RR = 4.02 (95% CI: 0.5–32.0) [NS] [8/1249 (0.6%) vs. 1/327 (0.2%) in intervention vs. control groups, respectively].

***Observational studies***			

Smith et al. 2007 [45]	UK. Observational study. Unselected women (N = 30,519) who had uterine artery Doppler performed 22–24 wks of gestation.	Studied the relationship between abnormal (mean pulsatility index in the top decile and a bilateral notch) vs. normal Doppler flow on the risk of antepartum stillbirth.	Antepartum SBR: adj. HR = 5.5 (95% CI: 2.8–10.6) in Doppler with mean pulsatility index in the top decile vs. controls. Antepartum SBR: adj. HR = 3.9 (95% CI: 2.0–7.8) in Doppler with a bilateral notch versus controls. Unexplained SBR: adj. HR 2.5 (95% CI: 1.1–5.6) in Doppler with mean pulsatility index in the top decile vs. controls. No association between Doppler with a bilateral notch and SB.

**Table 6:** Impact of umbilical artery and ductus venosus Doppler velocimetry on stillbirth and perinatal mortality

**Source**	**Location and Type of Study**	**Intervention**	**Stillbirths/Perinatal Outcomes**
***Reviews and meta-analyses***			

Baschat et al. 2004 [49]	Germany, Netherlands, UK, USA, Spain, Sweden. Review. 8 studies included. N = 320 fetuses with normal Doppler, N = 202 with elevated ductus venosus (DV) Doppler indices (N = 101 with umbilical artery absent or reversed end-diastolic flow (UA A/REDV), N = 34 with DV reversed atrial velocity (DV-RAV).	Assessed association of umbilical artery Doppler and ductus venosus Doppler with perinatal outcome in preterm growth-restricted fetuses.	Perinatal mortality was 5.6% (16/282) with normal DV, 11.9% (12/101) with UA A/REDV, 38.8% (64/165) with abnormal DV and 41.2% (7/17) with DV-RAV

Alfirevic and Neilson 1996 [50]	Australia, Sweden, UK (Chester, Edinburgh), South Africa (Tygerberg), Ireland (Dublin), Netherlands (Maastricht). Meta-analysis (Cochrane). 11 RCTs included (N = 6753 high-risk pregnant women).	Assessed the effects of Doppler umbilical artery waveform analysis (intervention) vs. no Doppler (controls) on obstetric care and fetal outcomes.	SBR: OR = 0.79 (95% CI: 0.46–1.34) [NS] [24/3325 vs. 31/3428 in intervention vs. control groups, respectively]. PMR: OR = 0.71 (95% CI: 0.50–1.01) [NS] [53/3433 vs. 75/3532 in intervention vs. control groups, respectively].

***Intervention studies***			

Baschat et al. 2003 [47]	Germany. Prospective cohort. N = 224 pregnancies with growth-restricted fetuses <37 weeks gestation.	Used logistic regression to assess the predictive ability of Doppler diagnosis of absent or reversed umbilical artery end-diastolic velocity, absence or reversal of atrial systolic blood flow velocity in the ductus venosus and pulsatile flow in the umbilical vein to predict stillbirth and perinatal mortality.	PMR: Umbilical artery waveform analysis most predictive compared to other Doppler modalities (R^2 ^= 0.49, P < 0.001) SBR: Umbilical artery waveform analysis most predictive compared to other Doppler modalities(R^2 ^= 0.48, P < 0.001). In cases of abnormal or reversed end-diastolic umbilical artery flow, venous pulsatility improved prediction of stillbirth.

Giles et al; DAMP Study Group 2003 [52]	Australia, New Zealand, Southeast Asia. Tertiary level referral hospitals. Multi-centre RCT. Pregnant women (N = 526) with twin pregnancies at 25 wks gestation.	Compared the impact of Doppler ultrasound umbilical artery flow velocity waveform analysis (intervention) vs. no Doppler (controls) on pregnancy outcomes. Standard ultrasound biometric assessment in both arms.	Fetal death (miscarriage + SB): OR = 0.14 (95% CI: 0.01–1.31) [NS] [0/262 vs. 3/264 in intervention vs. control groups, respectively. PMR: 9/1000 vs. 11/1000 live births in intervention vs. control groups, respectively [NS]

No authors listed 1997. [171]	France. 20 centres. Multicentre RCT. Low risk pregnant women (N = 3898) at 28 wks of gestation.	Compared the impact of umbilical Doppler 28–34 wks gestation (intervention) vs. no routine umbilical Doppler except in cases of clinical indication (controls).	SBR: OR = 0.40 (95% CI: 0.04–2.44) [NS] [2/1948 vs. 5/1943 in intervention vs. control groups, respectively]. PMR: OR = 0.33 (95% CI: 0.06–1.33) [NS] [3/1948 vs. 9/1943 in intervention vs. control groups, respectively].

Davies et al. 1992 [172]	UK (London). Single centre; unselected population. RCT. Singleton pregnancies (N = 2600) > 20 wks gestation.	Compared the impact of routine umbilical and uterine artery Doppler ultrasound to assess placental perfusion (intervention) vs. no Doppler (controls) on pregnancy outcomes. Standard ANC in both arms.	SBR: 11/1246 vs. 4/1229 in intervention vs. control groups, respectively. PMR (uncorrected): RR = 2.4 (95% CI: 1.00–5.76) [NS] [17/1246 vs. 7/1229 in intervention vs. control groups, respectively]. PMR (normally formed): RR = 3.95 (95% CI: 1.32–11.77). [16/1246 vs. 4/1229 in intervention vs. control groups, respectively].

Whittle et al. 1994 [173]	UK (Glasgow). RCT. Singleton pregnancies (N = 2986) < 26 wks gestation at 1st ANC visit. Doppler ultrasound at 26–30 wks and 34–36 wks gestation in all women.	Compared the impact of umbilical artery Doppler ultrasound revealed to clinician (intervention) vs. concealed from clinician (controls).	SBR: OR = 0.34 (95% CI: 0.10–1.07) [NS] [3 vs. 8 in intervention vs. control groups, respectively.]

***Observational studies***			

Hugo et al. 2007 [48]	South Africa (Cape Town). Secondary hospital. Case series. Singleton pregnant women (N = 572) referred for suspected poor fetal growth.	Investigated the use of a personal computer- based, continuous-wave Doppler machine by a trained midwife to assess umbilical artery flow velocity waveforms with respect to the resistance indices (RIs).	PMR: [RIs < P75]: 13.2 [RIs: P75-95]: 39.1 [RIs > P95]: 41.7 SGA (%): [RIs < P75]: 27.2% [RIs: P75-95]: 41.2% [Ris > P95]: 55.6%

Theron et al. 1992 [41]	South Africa. Prospective cohort study. Pregnant women (N = 127) with poor symphysis fundal growth (N = 39 abnormal Doppler flow velocimetry, N = 88 normal velocimetry).	Compared the impact of poor Doppler flow velocimetry of umbilical artery (exposed) with normal flow (unexposed).	PMR: OR = 33.2 (95% CI: 6.6–109.6; P < 0.000001). [43.6% vs. 2.3% in exposed vs. unexposed groups, respectively]. Fetal death (miscarriage + SB): [28.2% vs. 0% in exposed vs. unexposed groups, respectively; (P < 0.0005)].

Torres et al. 1995 [42]	Spain (Barcelona). Hospital Clinic. Prospective observational study over a 2-year period. Hypertensive pregnant women (N = 172; N = 166 with live births, N = 6 fetal deaths).	Assessed the use of umbilical artery Doppler in predicting SB. Compared the impact of absent (exposed) vs. normal end-diastolic velocity (unexposed).	SB: All had absence of end-diastolic velocity (sensitivity 100%). Fetal death (miscarriage + SB): 6/9 vs. 0/163 in absent vs. normal flow. Absent end-diastolic velocity in predicting fetal death: sensitivity: 100%, specificity: 98.2%, positive predictive value 66.7%, negative predictive value 100%.

#### Uterine artery Doppler waveform analysis

Several studies assessed whether uterine artery Doppler velocimetry in unselected populations could identify high-risk pregnancies, particularly those at risk of stillbirth. In a systematic review, Papageorghiou et al [44] reviewed 15 studies of routine Doppler assessments in pregnancy in unselected populations, and found that increased impedance to uterine artery flow was associated with increased risk of pre-eclampsia, fetal growth restriction and perinatal death, and that Doppler diagnosis of impeded artery flow appropriately identified ~40% of women who subsequently developed pre-eclampsia (6-fold increased risk with positive Doppler) and ~20% of fetal growth restriction cases (3.5-fold increased risk) ***[LOE: 1+] ***(Additional file 4).

Smith et al [45] reported a statistically significantly increased risk of antepartum stillbirth among women whose uterine artery Doppler flow velocimetry at 22–24 weeks had a pulsatility index in the top decile [adjusted Hazard Ratio (HR) = 5.5, 95% CI: 2.8–10.6] and among those with a bilateral notch (adjusted HR = 3.9, 95% CI: 2.0–7.8), compared with controls with normal Doppler flow ***[LOE: 2-]***.

An intervention RCT attempting to use Doppler in interventions to reduce rates of pre-eclampsia in France and Belgium by the Essai Régional Aspirine Mère-Enfant (ERASME) Collaborative Group [46] randomised women to either uterine Doppler (N = 1253) or placebo (N = 617). Women with abnormal waveforms in the intervention group were given low-dose aspirin to prevent pre-eclampsia; but despite the aspirin prescription, rates of pre-eclampsia were similar in the Doppler and placebo groups (28/1237 [2.3%] versus 9/616 [1.5%], respectively; RR = 1.55, 95% CI: 0.7–3.3 [NS]). The study found a non-significant elevated impact on perinatal mortality among Doppler-assessed fetuses compared to those whose mothers were given placebo (RR = 4.02; 95% CI: 0.5–32.0 [NS]).

#### Umbilical artery and venous Doppler waveform analysis

Baschat et al [47] performed umbilical artery and venous Doppler velocimetry in preterm growth-retarded fetuses (N = 224) to evaluate whether absent or reversed umbilical end-diastolic velocity, absence or reversed atrial systolic blood flow velocity in the ductus venosus, or pulsatile flow in the umbilical vein could predict stillbirth or perinatal death before 37 weeks' gestational age. Logistic regression analysis showed that umbilical artery waveform analysis was the Doppler application most predictive of perinatal mortality (R^2 ^= 0.49, P < 0.001) and stillbirth (R^2 ^= 0.48, P < 0.001). In fetuses with absent or reversed umbilical end flow, prediction of asphyxia and stillbirth was significantly enhanced by venous Doppler. Umbilical artery waveform analysis offered the highest sensitivity and negative predictive value, whereas ductus venosus and umbilical vein flow studies had the best specificity and positive predictive values for perinatal death ***[LOE: 1+]***.

Using personal computer-based, continuous-wave Doppler machines to assess umbilical artery flow velocity waveforms in women referred for suspected fetal growth restriction, Hugo et al. [48] found a direct association between resistance indices (RIs) and perinatal mortality rate (PMR) (PMRs of 13.2, 39.1 and 41.7 for women with RIs < P75, P75-95 and > P95, respectively) ***[LOE: 3]***.

Baschat et al. [49] conducted a review of studies (N = 8) where umbilical artery and ductus venosus Doppler studies were used to make decisions regarding delivery timing in pre-term growth-restricted fetuses (Additional file 5). One analysis in the review compared outcomes among fetuses with normal ductus venosus indices (N = 302) with fetuses with elevated ductus venosus index (N = 202), of whom N = 101 had absent or reversed umbilical end-diastolic flow and N = 34 had absence or reversal of atrial velocity in the ductus venosus. Perinatal mortality was 5.6% (16/282) among fetuses with normal Doppler, versus 11.9% (12/101) with absent or reversed umbilical end-diastolic flow, 38.8% (64/165) with abnormal ductus venosus index and 41.2% (7/17) with reversed atrial velocity in the ductus venosus. Abnormal ductus venosus results (N = 3 studies) effectively identified fetuses at risk of stillbirth at least 1 week prior to delivery, independent of umbilical artery waveform results, though questions remain about optimal delivery timing in growth-restricted pre-term fetuses ***[LOE: 1+]***.

Recently, researchers have focused on downstream impact on perinatal mortality of the use of umbilical artery Doppler ultrasound followed by appropriate interventions for the identified conditions. A Cochrane review by Neilson et al. [50] reviewed 11 RCTs (~7000 women) of Doppler ultrasound investigating umbilical artery waveforms in high-risk pregnancies. Compared to no Doppler ultrasound, evaluation with umbilical artery Doppler ultrasound in complicated pregnancies (especially with hypertension or presumed growth restriction) was associated with a non-significant trend toward lower perinatal mortality (OR = 0.71, 95% CI: 0.50–1.01) and stillbirth risk (OR = 0.79, 95% CI: 0.46–1.34), as well as significantly fewer inductions of labour (OR = 0.83, 95% CI: 0.74–0.93) and hospital admissions (OR = 0.56, 95% CI: 0.43–0.72) (Additional file 6). Whether a woman had received Doppler ultrasound was unassociated with fetal distress in labour or Caesarean section rates ***[LOE: 1+]***. A Cochrane protocol by Alfirevic et al indicates that a review is in progress to assess whether the use of umbilical artery, middle cerebral artery, and ductus venosus Doppler velocimetry improves subsequent obstetric care and fetal outcome [51].

Umbilical artery Doppler alone may not be any more effective than routine ultrasonography in some diagnostic assessments of fetal growth. An RCT of Doppler in twin pregnancies in New Zealand, Australia and Southeast Asia where all pregnancies were studied ultrasonographically to monitor fetal growth showed no statistically significant difference in perinatal mortality between groups on which umbilical artery Doppler was performed versus those with no Doppler [52].

##### Conclusion

Doppler ultrasound is a relatively new technique that has been applied to the study of fetal, placental and uterine circulatory dynamics. Despite its novelty, it has been evaluated by more RCTs than has any other biophysical test of fetal growth or well-being. In low-risk or unselected populations, there is little evidence that any form of routine Doppler velocimetry contributes to reductions in stillbirth rates (overall Grade C evidence). This lack of impact may be complex: Doppler ultrasound may not identify a sufficient proportion of flow abnormalities to measurably impact stillbirth incidence; Doppler-detected abnormalities may not be subsequently monitored appropriately with other tests of fetal well-being and serial Doppler testing; intervention based on abnormal Doppler may not work; or high rates of false-positives may unnecessarily expose the fetus to the risk of preterm birth, particularly if gestational age dating is inaccurate. Additionally, most existing studies are underpowered to detect small impacts on perinatal or maternal outcomes.

Uterine artery Doppler waveform analysis accurately identifies compromised fetuses at risk of stillbirth, especially in cases of placental underperfusion associated with pre-eclampsia and/or growth restriction, but no studies have shown any ability of subsequent intervention to prevent stillbirth. More studies are needed into the optimal timing of monitoring and intervention in cases of abnormal uterine artery waveforms.

Of all Doppler diagnostic studies of the fetus, umbilical artery and ductus venosus Doppler velocimetry are most predictive of fetal compromise associated with fetal growth restriction, and there is some evidence that timely and appropriate intervention for abnormal umbilical artery or ductus venosus waveforms can prevent stillbirths [53]. More evidence should soon be available: in addition to the Cochrane review of umbilical and fetal Doppler velocimetry, results are forthcoming of the multi-centre Trial of Umbilical and Fetal Flow in Europe (TRUFFLE) group, an RCT of timing of delivery in early pre-term fetal growth restriction based on early and late fetal Doppler venous changes versus cardiotocography. Such studies may shed light on the most appropriate and effective methods of fetal surveillance and optimal uses of Doppler velocimetry, including multi-vessel analysis [54].

Further studies are needed to assess whether such decision analytical models based on Doppler and other fetal surveillance findings for fetal growth restriction, pre-eclampsia, or congenital abnormalities could have any impact on stillbirths. Further research might also investigate other applications of Doppler velocimetry, including identifying women who should be given other screening tests, the comparative efficacy of Doppler versus other fetal surveillance methods, and contributing to the study of the pathophysiology of impaired placentation, uteroplacental and fetoplacental haemodynamics, and pre-eclampsia.

#### Pelvimetry

##### Background

Pelvimetry, or pelvic measurement in pregnant women with the intention of predicting likely cephalopelvic disproproportion of cephalic presentations (and thus their subsequent need for Caesarean section), can be performed by clinical manual examination, or with imaging techniques including conventional x-ray, computerised tomography scanning, or magnetic resonance imaging. Successful detection and management of cephalopelvic disproportion is thought to reduce the risk of obstructed labour and intrapartum stillbirth.

##### Literature-based evidence

We identified 1 Cochrane review and 1 other observational study of pelvimetry reporting perinatal outcomes (Table 7). The observational study by Fine et al [55] retrospectively analysed studies of x-ray pelvimetry (N = 100 trials) in cephalic presentations, comparing 3 prognostic indicators for vaginal delivery: the Thoms method, the modified Ball technique, and manual assessment. Neither pelvimetric method was significantly more accurate than manual assessment of the pelvis in predicting obstetric outcome, nor was any one method superior to the other.

**Table 7:** Impact of pelvimetry on stillbirth and perinatal outcomes

**Source**	**Location and Type of Study**	**Intervention**	**Stillbirths/Perinatal Outcomes**
***Reviews and meta-analyses***			

Pattinson et al. 1997 [56]	South Africa, U.S.A. Meta-analysis (Cochrane). 4 RCTs included (N = 895 women).	Assessed the effects of pelvimetry performed antenatally, intrapartum or postpartum (intervention) vs. no pelvimetry (controls) on PMR.	PMR: OR = 0.51 (95% CI: 0.18–1.42) [NS]. [5/449 vs. 10/446 in intervention vs. control groups, respectively].

***Observational studies***			

Fine et al. 1980 [55]	Retrospective study. N = 100 X-ray pelvimetry studies of cephalic presentations.	Compared the Thoms method of interpretation to the modified Ball technique for x-ray pelvimetry (comparing both to manual assessment of the pelvis) as prognostic indicators for safe vaginal delivery.	Uneventful nonoperative vaginal deliveries: 28.6% of patients with either inlet or midpelvic disproportion by the Thoms method, and in 22.5% of women with absolute disproportion in either plane by the modified Ball method. Prediction of obstetric outcome: Neither technique significantly more accurate than manual assessment, or than the other.

When implemented as part of a strategy of pregnancy management, pelvimetry appears to have no impact on stillbirth. A Cochrane review by Pattinson et al. [56] assessed RCTs implementing x-ray pelvimetry in cephalic presentations (N = 4 trials, N>1000 women). Pelvimetry was associated with increased rates of Caesarean section (OR = 2.17; 95% CI = 1.63–2.88) but had no impact on PMR (4 RCTs; OR = 0.51, 95% CI = 0.18–1.42 [NS]) ***[LOE: 1+] ***(Additional file 7).

##### Conclusion

There is little support for the use of pelvimetry to predict the need for Caesarean section in women with fetuses with cephalic presentations, as the dynamic and individual nature of maternal tissue changes during labour and fetal head moulding in the birth canal make antenatal pelvimetry a poor predictor of cephalopelvic disproportion. The practice may result in inadvertent harm to the mother by significantly increasing her risk of having a Caesarean section, without increasing the benefit to fetus or neonate, as pelvimetry shows no impact on stillbirth incidence. However, deficiencies in the existing studies included in Pattinson's meta-analysis should be noted: Parsons [57] attributed the increased perinatal mortality and birth asphyxia reported by Crichton [58] to lack of availability of electronic fetal monitoring, not to cephalopelvic disproportion. Even more problematic, the 2 deaths documented by Richards [59] occurred *in utero *prior to labour, which imply that cephalopelvic disproportion was not implicated. Treatment allocation strategies in all trials were of poor quality.

Given the deficiencies in existing studies, it remains plausible that other forms of imaging could effectively diagnose true cephalopelvic disproportion and avert stillbirth via Caesarean section in these cases. We classified the overall evidence as Grade C and found no evidence in support of using pelvimetry for preventing stillbirths.

#### Detection and management of maternal diabetes mellitus

##### Background

In pregnant women, pre-existing diabetes mellitus can cause severe complications for both mother and child during pregnancy and delivery, including congenital malformations, hypertension, pre-eclampsia, macrosomia and intrauterine fetal death [60-63]. Macrosomia (large size for gestational age) increases the risk to the fetus of birth trauma, including shoulder dystocia, bone fractures and brachial plexus injury, in addition to obstructed labour. Good metabolic control in the mother from prior to conception through the postpartum period reduces the risk of these complications [64-66]. Because pre-gestational diabetes is a known risk factor for stillbirth, women with this condition are usually offered intensive surveillance and management during pregnancy, which may include glycaemic control efforts through diet, exercise, and/or insulin therapy with glucose monitoring, frequent fetal surveillance using tests of fetal well-being, and/or induction at or before term. Despite widespread practice of this protocol, a recent UK study demonstrated a 4-fold higher rate of stillbirth among women with pre-gestational diabetes compared to non-diabetic women, with 83% of stillbirths unrelated to congenital malformations [60].

There is also substantial confusion surrounding optimal screening for and management of glucose intolerance and gestational diabetes. Gestational diabetes and impaired glucose tolerance are relatively common and, like pre-gestational diabetes, have been linked with adverse perinatal outcomes including stillbirth and shoulder dystocia as a consequence of fetal macrosomia [67,63,68]. However, how to best screen for gestational diabetes is controversial. The American Diabetes Association, the American College of Obstetricians and Gynecologists, and the World Health Organization recommend universal screening for gestational diabetes based on the conclusion that selective screening is inadequate [69-72], while the US Preventive Services Task Force concluded that there is insufficient evidence of benefit to justify universal screening for gestational diabetes. As with management of pre-gestational diabetes, intensive management of gestational diabetes includes glucose monitoring, dietary regulation, and tests of fetal well-being. The need for insulin therapy is usually less for gestational diabetes than with pre-gestational diabetes.

##### Literature-based evidence

Our literature search identified 4 reviews and 12 other intervention and observational studies reporting perinatal outcomes after management of women with any form of diabetes mellitus (Table 8).

**Table 8:** Impact of detection and management of maternal diabetes mellitus on stillbirth and perinatal mortality

**Source**	**Location and Type of Study**	**Intervention**	**Stillbirths/Perinatal Outcomes**
***Reviews and meta-analyses***			

Russell et al. 2007 [67]	USA. Review. 3 RCTs included, 1 reported perinatal outcomes.	Assessed the impact of treatment of gestational diabetes on perinatal outcomes.	Serious perinatal complication (shoulder dystocia, nerve injury, fracture, or perinatal death): 67% reduction (1 RCT). Macrosomia: 53% reduction (1 RCT). 2 of 3 studies lacked power to detect a difference in perinatal outcomes.

Tuffnell et al. 2003 [73].	US, UK. Review (Cochrane). 3 RCTs and quasi-RCTs included (N = 223 pregnant women with impaired glucose tolerance)	Assessed the effect of treatments for impaired glucose tolerance on perinatal outcome.	PMR: Insufficient data to assess. Reduction in BW > 90% percentile: RR = 0.55, 95% CI: 0.19–1.61) [NS]

Boulvain et al. 2001 [84]	USA. Review (Cochrane). 1 RCT included (N = 200 term diabetic women).	Assessed the effects of a policy of elective delivery (intervention) vs. expectant management (controls) on maternal and perinatal mortality and morbidity.	PMR: RR not estimable [0/100 in both groups].

Mukhopadhyay et al. [81]	United Kingdom. Review (non-Cochrane). 5 RCTs included (N = 182 diabetic pregnant women).	Compared the impact of continuous subcutaneous insulin infusion (CSII) (intervention) with multiple daily insulin injections (MDI)/intensive conventional therapy (ICT) (controls).	SBR: OR = 2.50 (95% CI: 0.53 – 11.77) [NS]; P = 0.25 [6/94 (6.4%) vs. 1/88 (1.1%) in intervention and control groups, respectively].

***Intervention studies***			

Hod et al. 2008 [82]	Multicentre, multinational. 63 study sites in 18 countries. Open-label RCT. N = 322 pregnant type I diabetic women (N = 157 intervention group, N = 165 controls).	Assessed the impact of mealtime insulin Aspart (IAsp) (intervention) with human insulin (HI) (controls), both in combination with basal neutral protamine Hagedorn (NPH) insulin.	SBR: one in each group. PMR: 14 vs. 22/1000 births in intervention and control groups, respectively.

Bancroft et al. 2000 [174]	UK (West Yorkshire). Hospital-based study (district general hospital and large teaching hospital). RCT. Pregnant women (N = 68) with impaired glucose tolerance.	Compared the effects in a group that monitored plasma glucose up to 4× daily (intervention) vs. an unmonitored group (controls). Median plasma glucose measurements in intervention group = 118 (range: 0–500); 19% of women in the monitored group treated with insulin.	PMR: 0/36 vs. 0/32 in intervention vs. control groups, respectively [NS] NMR: 0/36 vs. 0/32 in intervention vs. control groups, respectively [NS]

Karmon et al. 2009 [175]	Israel. Retrospective cohort study.	Measured pregnancy outcomes in women with diet-controlled gestational diabetes subject to a routine policy of labour induction at 40 weeks.	Rates of dystocia, congenital malformation, and macrosomia higher in offspring of diet-controlled diabetic patients than non-diabetic patients. Perinatal mortality: no difference when adjusted for confounders. SBR: Higher in non-diabetic women after 40 weeks (likely due to policy of induction of diabetic women at 40 weeks).

Langer et al. 1994 [74]	USA (Texas). Population-based. Prospective study. Pregnant women (N = 2461; N = 1145 intervention, N = 1316 diabetic controls) with gestational diabetes and a non-diabetic control group (N = 4922).	Compared the impact of intensified management (intervention) vs. conventional management (diabetic controls) vs. non-diabetic controls on adverse pregnancy outcomes.	SBR: 1/1000 (N = 1145) vs. 4/1000 (N = 1316) vs. 4/1000 (N = 4922) in the intervention, diabetic controls and non-diabetic controls, respectively. Macrosomia, Caesarean section, metabolic complications, shoulder dystocia, NICU days, and respiratory complications lower among intervention group than diabetic controls; comparable to non-diabetic controls.

***Observational studies***			

Aucott et al. 1994 [83]	USA. Prospective study. Pregnant patients (N = 78) with pre-gestational diabetes vs. matched non-diabetic pregnant controls (N = 78).	Compared the impact on stillbirth of treatment with insulin (exposed) by either infusion pump (N = 20) or split-dose therapy (N = 58) vs. matched controls (unexposed).	SB: 1/78 vs. 0/78 in exposed vs. unexposed groups, respectively.

Fadel et al. 1982 [176]	USA. Observational study. N = 84 women with gestational diabetes, N = 23 women with pre-gestational diabetes.	Compared the impact of a protocol of intensive diabetes management including frequent prenatal visits, strict metabolic control, antepartum monitoring including estriols and contraction stress tests, liberal hospitalization policy, fetal lung maturity assessment, and intrapartum fetal monitoring.	SB: 1 antepartum SB among women with gestational diabetes (due to true knot in cord); 0 SB in women with pre-gestational diabetes. Caesarean section: 15.4% among gestational diabetics, 56.5% among pre-gestational diabetics. Macrosomia: 20% among gestational diabetics, 13% among pre-gestational diabetics.

Banerjee et al. 2004 [77]	India. Antenatal clinic. Prospective study. N = 240 women with gestational (GDM) and pre-gestational (PGDM) diabetes mellitus.	All women were placed on exercise, diet, and/or insulin therapy. And were divided into three groups based on blood glucose levels and HbA1C: Tight Glycaemic Control (TC), Acceptable glycaemic control (AC) and uncontrolled glycaemic group (UC).	PMR: 4.16% vs. 18.2% vs. 22.2% in the TC, AC and UC subgroups of GDM. PMR: 0% vs. 20% vs. 40% in TC, AC and UC subgroups of PGDM.

Gonzalez-Quintero et al. 2007 [76]	USA. Centralised perinatal database. Retrospective study on prospective data. N = 3,218 women participating in the outpatient GDM management programme.	Compared the impact on stillbirth of women whose blood glucose levels were controlled vs. those uncontrolled.	SBR (n,%): 2 (0.1) vs. 4 (0.3) in women with controlled vs. uncontrolled GDM.

Huddle et al 1993 [79]	South Africa (Soweto). Retrospective study. Patient records (N = 733 women; N = 348 with gestational diabetes).	To assess the impact on perinatal mortality of the use of a specialised service for diabetic pregnant women (intervention) vs. non-use of the service (controls).	PMR: 3.7% vs. 15.6% in intervention vs. controls, respectively.

McElvy et al. 2000 [78]	USA. Retrospective before-after study. Pregnant Type-1 diabetic women enrolled peri-conceptionally (N = 306; N = 111 before, N = 103 during, N = 92 after programme).	To evaluate the impact on perinatal mortality of a focused preconceptional and early pregnancy programme for Type 1 diabetes including strict glucose control and antepartum fetal surveillance from 32 weeks gestation until delivery.	PMR: 3% vs. 2% vs. 0% before, during, and after the programme, respectively.

Landon et al. 1992 [80]	USA (Ohio). 2 teaching hospitals. Prospective cohort study. Pregnant women (N = 114) with insulin-dependent diabetes. Non-stress testing was begun weekly at 28–30 wks and 2× weekly at 32 wks (N = 1676 NSTs performed (14.7+/-3.2 tests per patient)). 8% of tests (N = 134) non-reactive, necessitating a biophysical profile.	To determine whether maternal vascular disease and/or glycaemic control are associated with fetal condition in diabetic pregnancies by comparing the effect of reactive (exposed) vs. non-reactive (unexposed) NST on perinatal outcomes.	Fetal death (miscarriage + SB): 1/114. N = 10 deliveries among patients with abnormal test results. No significant differences in ambulatory glucose profile data in exposed vs. unexposed groups. No significant differences in glycaemic parameters in women delivered for suspected fetal compromise vs. nonintervention group. 8/20 (40%) women with nephropathy or hypertension required delivery for fetal well-being, vs. 2/94 women (2%) without nephropathy or hypertension but with abnormal test results (P < 0.001).

Nachum et al. 2001 [177]	Israel. Prospective controlled study. Pre-gestationally and gestationally diabetic women (N = 681 women; N = 801 pregnancies) recruited 1986–1989.	Compared the impact of diabetic pregnancies managed by hospitalisation vs. those managed by ambulatory care.	NMR: 1/394 vs. 0/407 in hospitalised vs. ambulatory care groups, respectively.

Intensive management

Tuffnell et al. [73] undertook a systematic review of RCTs (N = 3) of strategies for intensive management of women with gestational diabetes and/or impaired glucose tolerance in pregnancy (N = 223), including obstetric monitoring, dietary regulation, and insulin therapy in some cases. No trials of treatments for gestational diabetes were included, however, and of the trials of treatments for impaired glucose tolerance that reported perinatal outcomes, only 1 trial (N = 68) of mostly Hispanic patients reported birth trauma incidence. This study found no significant difference between the group receiving intensive management versus any minimal treatment (RR = 0.37, 95% CI: 0.02–8.86 [NS]). Caesarean section rates were not significantly different ***[LOE: 1+]***.

A number of studies compared different strategies of management of diabetic pregnant women, including glycaemic monitoring and control, diet and exercise regimens, and insulin treatments. In a prospective population-based study of intensive management of women with diabetes mellitus (N = 1145), Langer et al [74] observed that an intensively managed group of women with diabetes had rates of stillbirth and neonatal complications similar to non-diabetic controls (N = 4922), while diabetic women given conventional management (N = 1316) had higher rates of these adverse outcomes ***[LOE: 2+]***. Crowther et al. [75] randomly assigned women with gestational diabetes to receive either dietary advice, blood glucose monitoring, and insulin therapy as needed, or routine care, and reported decreased perinatal mortality associated with the intervention compared to the routine care group (0 versus 5 perinatal deaths in intervention versus routine care groups, respectively, P = 0.07) ***[LOE: 1-] ***(Additional file 8).

In the US, Gonzalez-Quintero et al. [76] conducted an observational study to assess the impact on perinatal outcomes of glycaemic control among women with gestational diabetes attending an outpatient gestational diabetes management programme. Over one-third of the participants in the group with poor glycaemic control had at least one of the factors in the composite study outcome variable (macrosomia, large-for-gestational-age, hypoglycaemia, jaundice, or stillbirth) compared with only 24% in the well-controlled group (P < 0.001) ***[LOE: 2-]***. A somewhat less rigorous intervention study in India (N = 240 mothers) by Banerjee et al. [77] sought to assess the incidence of fetal complications in both gestational and pre-gestational diabetic pregnant women with tight versus acceptable or uncontrolled glycaemic control. In women with pre-gestational diabetes, the tight glycaemic control group had the lowest perinatal mortality (4.16% versus 18.8% versus 22.2% in tight, acceptable, and uncontrolled glycaemic control groups, respectively). In women with gestational diabetes, the same trend was noted with even greater impact of tight glycaemic control (0% versus 20% versus 40% in tight, acceptable, and uncontrolled glycaemic control groups, respectively). No statistical significance measures were provided ***[LOE: 1-]***.

In the US, McElvy et al. [78] conducted an evaluation of a programme that worked to achieve strict glycaemic control before and during early pregnancy in women with pre-gestational diabetes (N = 306), with intensive antepartum surveillance from 32 weeks gestation until delivery. Perinatal mortality declined steadily from 3% before the programme to a rate comparable to general population levels after completion of the programme ***[LOE: 1-]***. In Soweto, South Africa, Huddle et al [79] reported that a programme involving gestational diabetic women given special services during pregnancy observed lower rates of perinatal mortality among women participating in the service compared to non-users of the service (3.7% versus 15.6%, respectively), but no statistical data was furnished ***[LOE: 3]***.

A descriptive study by Landon et al. [80] of non-stress testing (with delivery for non-reactive tests) in women with pre-gestational diabetes found no differences in glycaemic parameters in women delivered for suspected fetal compromise compared to a non- intervention group, suggesting the possibility that glycaemic control was unrelated to fetal compromise ***[LOE: 3]***.

Several studies assessed the impact on perinatal outcomes of mode of insulin treatment. Mukhopadhyay et al. [81] conducted a meta-analysis of RCTs (6 trials) comparing the impact of continuous subcutaneous insulin infusion versus multiple-dose insulin on glyacemic control and pregnancy outcome in diabetic women. Neither glycaemic control nor pregnancy outcomes were different between treatment groups ***[LOE: 1++] ***(Additional file 9). In Israel, Hod et al. [82] conducted a small RCT (N = 322 women) comparing the impact on fetal and perinatal outcomes of insulin aspart, a fast-acting insulin analogue, versus human insulin therapy in women with pre-gestational diabetes. A slight trend toward lower perinatal mortality was observed in the insulin aspart group compared to the human insulin group (14/1000 versus 22/1000, respectively), but no statistical significance data was reported ***[LOE: 1-]***. A study of insulin therapy using either pump infusion or split-dose therapy found no difference in rates of perinatal mortality in the treated groups compared to non-diabetic controls [83].

Elective delivery

A Cochrane review by Boulvain et al. [84] assessed trials of expectant management versus elective delivery in term women with pre-existing diabetes mellitus; only 1 trial was included (N = 200), which documented no perinatal mortality in either group ***[LOE: 1+] ***(Additional file 10).

##### Conclusion

While data from RCTs is scarce, the limited evidence, largely from clinical management of diabetic patients (Grade C evidence), suggests that pregnant women who do not have vascular complications and with good glycaemic control do not as a group have an increased risk of stillbirth above the general population [85]. In settings with poor access to care and high prevalence of gestational diabetes, perinatal mortality has been shown to be reduced with diabetes screening and treatment [79]. Although retrospective studies reveal declines in the incidence of stillbirth among diabetic women in low-prevalence populations where diabetes care is available [86], few prospective trials have been able to show any impact on stillbirths or perinatal mortality, largely owing to the high sample sizes required for such studies [63]. Adequate glycaemic control and monitoring during pregnancy are a reasonable means for reducing stillbirths that has been shown to reduce many complications that may be related to stillbirth, including congenital anomalies and macrosomia. Where feasible, particularly in high-prevalence settings, large studies with rigorous designs may help confirm this recommendation.

### Advanced monitoring in pregnancy

#### Antenatal fetal heart rate monitoring using cardiotocography

##### Background

Antenatal fetal surveillance studies are widely used to assess fetal well-being and to identify the compromised fetus. Fetal heart rate patterns are a principal component of the majority of tests of fetal well-being, and cardiotocography, where it is available, is the method frequently used to electronically record the fetal heart rate. In high-income countries, beginning in the third trimester, cardiotocographic monitoring (electronic fetal monitoring) has largely replaced intermittent auscultation (periodic listening to the fetal heart rate using a stethoscope or handheld Doppler device) for monitoring fetal heart rate. During the antenatal period, external cardiotocography is used, which employs a Doppler ultrasound transducer; the mother's uterine contractions are also monitored using a pressure transducer, with both transducers strapped to the mother's abdomen while recording is in process.

Antenatal fetal heart monitoring includes both non-stress tests (NST) and contraction stress tests (CST). Fetal heart rate patterns are classified as either reassuring (reactive), non-reassuring (non-reactive) or abnormal, considering heart rate baseline, variability, and decelerations. In high-income countries, particularly the US, the NST is widely accepted as a primary fetal surveillance tool in high-risk pregnancies. It may be used in combination with ultrasound testing to observe fetal movement and amniotic fluid indices. When an NST is non-reactive, its poor predictive value (< 50%) warrants confirmatory testing with CST or a complete BPP [87]. During a CST, uterine contractions are induced (usually with intravenous oxytocin, but contractions can be generated using nipple stimulation) and subsequent fetal heart accelerations and decelerations are monitored to detect possible cases of uteroplacental insufficiency. It is important to note that antenatal fetal heart monitoring is gestational-age dependent; at 28 weeks' gestation, only about 60% of normally formed fetuses will have reactive non-stress testing due to immaturity of the fetal autonomic nervous system rather than placental insufficiency.

Variation between practitioners in interpretation of cardiotocographic tracings can potentially lead to inappropriate intervention, or false reassurance without appropriate intervention. Tracings are notoriously difficult to interpret, as fetal sleep patterns, labour progress, external stimuli, and opiate administration to the mother can alter fetal heart rate changes in the absence of fetal hypoxia or distress [2]. Additionally, as indications of fetal distress often indicate early delivery, the gestational age threshold at which antepartum testing is initiated will depend on an institution's ability to care for very preterm neonates, which, in general, is earlier in high-income settings than in lower-resource settings [21].

##### Literature-based evidence

We identified 1 Cochrane review and 4 observational studies testing the impact of antepartum cardiotocography (including both NST and CST) on perinatal outcomes (Table 9).

**Table 9:** Impact of antepartum cardiotocography on stillbirth and perinatal outcomes

**Source**	**Location and Type of Study**	**Intervention**	**Stillbirths/Perinatal Outcomes**
***Reviews and meta-analyses***			

Pattison and McCowan 1999 [91]	UK, Australia. Meta-analysis (Cochrane). 3 RCTs included (N = 1279).	Compared the impact of electronic fetal monitoring with an antenatal CTG (intervention) vs. a control group where the results of the CTG were withheld from the caregiver or no monitoring was done on perinatal morbidity and mortality and maternal morbidity.	PMR (excluding lethal abnormalities): OR = 2.65 (95% CI: 0.99–7.12) [NS]. [12/651 vs. 4/628 in intervention and control groups, respectively].

***Observational studies***			

Evertson et al. 1978 [90]	USA. Case series. N = 746 pregnant women undergoing 1119 CSTs.	Assessed the incidence of fetal deaths within one week of a negative CST.	SBR: 7/680 patients (1%) within 1 week of a negative CST. Fetal death in most cases resulted from factors other then uteroplacental insufficiency (umbilical cord accident, malformations, and placental abruption)

Flynn et al. 1982 [88]	UK. RCT. Pregnant patients (N = 300) with non-stress antepartum cardiotocography tracings (N = 569; N = 144 intervention, N = 156 controls).	Compared impact on pregnancy outcomes of revealing cardiotocography results to clinician (intervention) vs. concealing results (controls).	SBR+NMR: Significant association with 'non-reactive' traces. Significant association of nonreactive traces with rates of fetal growth restriction, admission to special care baby unit, Apgar scores at 1 and 5 min.

Freeman et al. 1982 [92]	USA and Canada. 18 institutions. Comparison of diagnostic tests. Pregnant women (N = 6168; N = 4626 CST, N = 1542 NST) with increased risk for uteroplacental insufficiency.	Compared the impact on fetal outcomes of CST (intervention) vs. NST (comparison) for primary fetal surveillance.	PMR: 8.4/1000 vs. 21.4/1000 in intervention vs. comparison groups, respectively (P < 0.05). After correction for congenital anomalies and unrelated causes: 3.5/1000 vs. 7.1/1000 intervention vs. comparison groups, respectively (P < 0.05). Fetal death (miscarriage+SB): 1.1/1000 vs. 7.8/1000 in intervention vs. comparison groups, respectively (P < 0.05). After correction: 0.4/1000 vs. 3.2/1000 in intervention vs. comparison groups, respectively (P < 0.05).

Rayburn et al. 1980 [89]	USA (Lexington). University hospital. Prospective cohort study. High-risk clinic patients (N = 561) who had undergone NST within one week prior to delivery.	Compared the association of reactive vs. non-reactive NSTs vs. no testing with fetal outcomes.	SB: 1/509 in reactive non-stress group (cord accident). Corrected PMR: 1/509 vs. 2/22 vs. 20/1000 in reactive, non-reactive and no testing groups, respectively (P < 0.05). Fetal compromise: 4%, 36%, and 13% in reactive, non-reactive and no testing groups, respectively (P < 0.001)

Several studies described how well NST and CST results correlated with subsequent perinatal outcome. An RCT by Flynn et al [88] identified a significant association of NST tracings (N = 569 tracings, N = 300 patients) with stillbirths, neonatal deaths, fetal growth restriction, complications of intrauterine hypoxia, and low Apgar scores ***[LOE: 1+]***. An observational study of high-risk pregnant women (N = 561) by Rayburn et al [89] assessed NST tracings within 1 week of delivery along with subsequent perinatal outcome. Perinatal mortality in patients with reactive NSTs was comparable to that of patients with no apparent antepartum complications (1/509 versus 6/1408, respectively), and significantly lower than among untested patients or patients with nonreactive NSTs (20/1000 and 2/22, respectively, P < 0.05) ***[LOE: 2-]***. Evertson et al [90] assessed the rates of fetal death within 1 week of negative CST results in a cohort of patients (N = 680), identifying only 7 deaths, of which most were due to factors other than placental insufficiency, suggesting that the test had low rates of false negatives.

A review by Pattison and McCowan [91] included 4 studies (N = 1,588 pregnancies) of the impact of cardiotocography use on perinatal mortality in high or intermediate risk pregnancies (Additional file 11). The trial reported a trend toward increased perinatal mortality in the cardiotocography group versus controls receiving no monitoring or whose cardiotocography results were concealed from the clinician; (3 trials, N = 1279 pregnancies, OR = 2.85, 95% CI: 0.99–7.12). Four of the 7 deaths in the largest trial reporting perinatal deaths were associated with fetal infection and prematurity, suggesting these deaths were not associated with the intervention. There was no increase in the incidence of elective Caesarean section or induction of labour ***[LOE: 1++]***.

Freeman et al [92] compared results from the NST (N = 1542 women) to the CST (N = 4626) for primary fetal surveillance and found a nearly 8-fold higher risk of antepartum fetal death in the NST group (7.8/1000 versus 1.1/1000, respectively, P < 0.05). Corrected for congenital anomalies and unrelated causes, the differential in antepartum fetal death remained 8-fold higher in the NST group (3.2/1000 versus 0.4/1000, respectively, P < 0.05) ***[LOE: 2+]***.

##### Conclusion

There are relatively few RCTs of antepartum fetal heart rate surveillance (Grade C evidence), though observational studies clearly indicate a correlation between non-reassuring cardiotocographic traces and adverse perinatal outcomes, including stillbirth. Although there is a Cochrane review on antepartum cardiotocography [91] showing a trend toward increased risk of perinatal mortality, the study was underpowered to detect such an impact, and the causes of the deaths observed appeared unlikely to have been preventable. While RCTs are lacking to evaluate an impact of NSTs in reducing stillbirth, apparent reductions in stillbirth rates have followed the incorporation of the NST into protocols for management of high-risk pregnancy in the United States [21]. As with fetal movement monitoring and umbilical Doppler velocimetry, the rate of false-positives with a non-reactive NST is high enough that additional testing, whether CST or BPP, is advised to guard against unnecessary intervention.

When used experimentally as a primary fetal surveillance tool, CST use was associated with lower rates of antepartum stillbirth [92], suggesting that it could be useful for primary fetal surveillance testing in pregnancies at highest risk of stillbirth, including those of women with hypertension, diabetes, and intrauterine growth restriction. As it typically uses intravenous oxytocin to stimulate uterine contractions, the CST is more technologically intensive than the NST and can induce labour; thus, it is contraindicated for patients with placenta previa, placental abruption, previous classical Caesarean section, and premature rupture of membranes [87]. The CST should be conducted cautiously in cases of multiple pregnancy, incompetent cervix, and polyhydramnios.

#### Fetal biophysical profile (BPP) test scoring for assessing high-risk pregnancy

##### Background

The BPP was conceptually derived from the Apgar score that is used to rate the condition of the newborn [93]. Using NST monitoring with cardiotocography and fetal ultrasound, the BPP collects 5 indicators of fetal well-being: fetal heart rate reactivity, breathing movements, gross body movements, muscular tone and qualitative amniotic fluid volume. Indications of fetal compromise often suggest the need for early delivery by induction of labour or Caesarean section.

##### Literature-based evidence

We identified one Cochrane review and four other observational studies of BPP reporting perinatal mortality outcomes (Table 10). A multi-institutional study in the US by Freeman et al [94] compared antepartum BPP versus NST, and reported that the fetal death rate among false negatives was several times lower among fetuses tested with BPP compared to the NST, though rates of false positive results with BPP were higher than with NST. Several other observational clinical studies were identified but had insufficient rigor or size to contribute meaningfully to the evidence base associating BPP results with stillbirth incidence [95-97].

**Table 10:** Impact of fetal BPP scoring on stillbirth and perinatal outcomes

**Source**	**Location and Type of Study**	**Intervention**	**Stillbirths/Perinatal Outcomes**
***Reviews and meta-analyses***			

Lalor et al. 2008 [1]	USA, UK. Meta-analysis (Cochrane). 4 RCTs included (N = 2839 pregnant women).	Compared the effects of complex (BPP; intervention) vs. simple fetal monitoring (cardiotocography and maximum pool depth) (controls).	PMR (including major malformations): RR = 1.33 (95% CI: 0.60–2.98) [NS] [13/1405 vs. 10/1434 in intervention vs. control groups, respectively]. PMR: RR = 1.30 (95% CI: 0.58–2.92) [NS] [13/1405 vs. 11/1434 in intervention vs. control groups, respectively].

***Observational studies***			

Awad 1991 [101]	Egypt. Al Fayrouz Hospital. Before-after study. N = 319 women (N = 160 intervention, N = 159 controls). Routine BPP introduced in 1990; compared to historical controls without BPP at same hospital prior to 1990.	Assessed the impact on perinatal mortality of introduction of routine BPP (intervention) vs. historical controls.	SBR: 0/1000 vs. 6/29/1000 in intervention vs. control groups, respectively. PMR (excluding malformations and alloimmunization disorders): 6.25/1000 vs. 25.16/1000 in intervention vs. control groups, respectively.

Golde et al. 1984 [97]	USA. University of Southern California Medical Centre and Women's Hospital, Los Angeles, Case series. Pregnant diabetic women (N = 107) vs. historic controls (N = 140) undergoing antepartum fetal surveillance.	Compared the impact of a package of nonstress heart rate testing, backed up by either fetal BPP or CST twice weekly (intervention), vs. weekly NSTs and daily plasma estriols (controls).	SB: 3/107 vs. 1/140 in intervention vs. control groups, respectively. 0 unexplained losses in either group.

de la Vega A 2002 [95]	Puerto Rico. Private clinic. Case series. Pregnancies (N = 1810) ≥20 wks gestation. High-resolution sonograph was performed in each trimester; BPP in 3rd trimester if risk factor was identified.	To assess the impact of testing fetal well-being using sonography and BPP in clinic cases (intervention) compared with the US average.	SBR: 14/1810 (7.7/1000 births) in this series vs. the U.S. national average of 6.7–7.8/1000 births.

Kennelly et al. 2007 [96]	UK. Single tertiary centre. Retrospective study. Records from Fetal Medicine Database, 2000–2005. Pregnant women (N = 39) with SGA twins (19 monochorionic sets, 13 dichorionic sets) with absent or reversed end diastolic flow in the umbilical artery.	To assess the impact of active monitoring with daily BPP after estimated fetal weight ≥500 g in both twins and gestational age ≥24 wks. Delivery was timed based on abnormal BPP, two equivocal BPP within 12 h or gestational age ≥32 wks.	Fetal death (miscarriage+SB): None.

Lalor et al. [1] undertook a Cochrane review of RCTs of variable quality (5 trials, N = 2839 women) comparing BPP with other forms of fetal assessment in women with high risk pregnancies (Additional file 12). There were no significant differences among pregnancies evaluated by by the various methods of fetal assessment in perinatal mortality (RR = 1.33, 95% CI: 0.60–2.98) or Apgar score <7 at 5 minutes (RR = 1.27, 95% CI: 0.85–1.92) ***[LOE: 1+]***.

##### Conclusion

Compared with conventional fetal monitoring, which is based primarily on cardiotocography/NST, BPP appears to offer no improvement in pregnancy outcomes (Grade C evidence). Although rates of false negative test results of the BPP are low, rates of false positives are high. The total number of subjects included in the meta-analysis remains small (N = 2974), and some of the studies carry a high risk of allocation bias. Although there is no consensus on optimal protocol for fetal monitoring, studies demonstrate that it is reasonable to implement the modified BPP (which consists of an NST and amniotic fluid volume measurement only), with full BPP or other tests such as the CST reserved for abnormal test results [98]. This passive and rapid approach to monitoring has been shown to be more cost-effective without diminishing its predictive value [99]. For reasons of cost and simplicity, the modified BPP may also prove to be more implementable than the full BPP in low-/middle-income country contexts with ultrasound and cardiotocographic capacity [100,101].

#### Vibroacoustic stimulation

##### Background

Adjunctive vibroacoustic stimulation with the NST is used less often than CST or BPP after non-reassuring NST results. Vibroacoustic stimulation provokes fetal response using a sound-emitting device placed on the maternal abdomen near the fetal head, along with simultaneous cardiotocography to document fetal movements and heart traces. Theoretically, the resultant startle reflex in the fetus and subsequent fetal heart rate acceleration or transient tachycardia following vibroacoustic stimulation provide reassurance of fetal well-being and minimise unnecessary intervention for nonreactive tests [102].

##### Literature-based evidence

Our literature search identified one Cochrane review on vibroacoustic stimulation and four other observational/interventional studies (Table 11). In a Cochrane review of vibroacoustic stimulation in conjunction with tests of fetal well-being (9 RCTs, N = 4838 women), Tan et al. [102] found that fetal vibroacoustic stimulation reduced the incidence of non-reactive antenatal cardiotocography tests (7 trials; RR = 0.62, 95% CI: 0.52–0.74) but the study was underpowered to detect impact on perinatal mortality of vibroacoustic stimulation compared to no or mock stimulation (RR = 0.32, 95% CI: 0.01–7.78 [NS]) ***[LOE: 1+] ***(Additional file 13).

**Table 11:** Impact of vibroacoustic stimulation on stillbirth and perinatal outcomes

**Source**	**Location and Type of Study**	**Intervention**	**Stillbirths/Perinatal Outcomes**
***Reviews and meta-analyses***			

Tan et al. 2001 [102]	Australia, USA, Mexico, Greece, Turkey, Thailand. Meta-analysis (Cochrane). 8 RCTs included (N = 4838 women).	Compared the impact of fetal vibratory acoustic stimulation (intervention) vs. mock or no stimulation (controls).	Non-reactive antenatal cardiotocography test (7 RCTs): RR = 0.62 (95% CI: 0.52–0.74) [176/2244 vs. 286/2239 in intervention vs. control groups, respectively.] PMR (2 RCTs): RR = 0.32 (95% CI: 0.01–7.78) [NS] [0/476 vs. 1/464 in intervention vs. control groups, respectively].

***Intervention studies***			

Papadopoulos et al. 2007 [103]	Greece. RCT. Pregnant women (N = 2833).	Compared the effect of vibroacoustic stimulation with a 3-s stimulus with an artificial larynx (repeated if BPP remained abnormal for 30 min; intervention) vs. no stimulation (controls).	Fetal death (miscarriage+SB): 10/1349 (0.74%) vs. 9/1484 (0.6%) in intervention vs. control groups, respectively [NS]

Petrovic 1998. [178]	Croatia. RCT. Singleton pregnancies (N = 494; N = 168 intervention, N = 326 controls). Groups were monitored using the modified BPP; given vibroacoustic stimulation in absence of fetal activity at the start of the BPP.	Compared the impact of vibroacoustic stimulation (intervention) vs. no stimulation (controls).	SBR: 2/168 vs. 4/326 in intervention vs. control groups, respectively.

Sood 2007 [104]	India. RCT. Singleton high-risk pregnancies (N = 214).	Compared the effect of vibroacoustic stimulation (intervention) vs. mock stimulation (controls). Both arms also had modified BPP conducted.	PMR: OR = 0.62 (95% CI: 0.05–5.57) [NS] [2/110 (1.8%) vs. 3/104 (2.9%) in intervention vs. control groups, respectively.

***Observational studies***			

Salamalekis et al. 1994 [179]	Greece. Case series. High-risk pregnancies (N = 180).	Compared the association with fetal deaths of reactive (study group) vs. non reactive (controls) NST results for tests conducted 24 hrs before delivery.	Fetal death (miscarriage+SB): 1 (0.67%) vs. 2 (6.25%) in intervention vs. control groups, respectively.

In Greece, Papadopoulos et al. [103] found no difference in fetal deaths in a group of women (N = 2833) randomised to vibroacoustic stimulation or no stimulation (10 [0.74%] versus 9 [0.6%], respectively [NS]). In India, Sood [104] conducted an RCT comparing vibroacoustic to mock stimulation and found no statistically significant difference in PMR (1.8% versus 2.9%, respectively; OR = 0.62, 95% CI: 0.05–5.57, P = 0.67).

##### New meta-analysis

**Figure 2:**
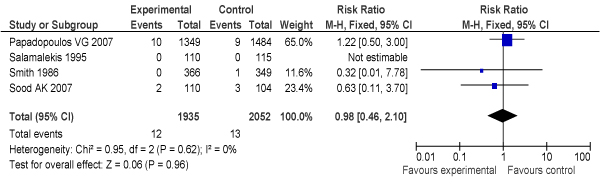
**Meta-analysis (Forest plot, Fixed effects model) of impact of vibroacoustic stimulation versus mock or no stimulation on intrauterine or perinatal deaths**.

**Figure 3:**
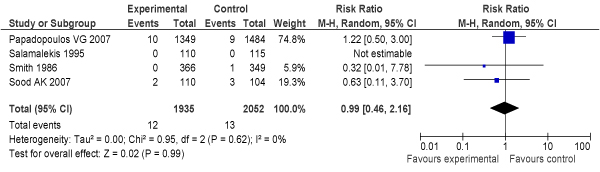
**Meta-analysis (Forest plot, Random effects model) of impact of vibroacoustic stimulation versus mock or no stimulation on intrauterine or perinatal deaths**.

We conducted a meta-analysis using available RCTs comparing outcomes (intrauterine or perinatal deaths) of pregnancies monitored by vibroacoustic stimulation compared to mock or no stimulation (4 trials, N = 1935 women in the vibroacoustic group, N = 2052 women in the mock or no stimulation group) (Figures 2 and 3). Three trials reported perinatal deaths and 1 reported intrauterine deaths, so the outcome of the meta-analysis was expanded and renamed as 'intrauterine or perinatal deaths' to include all 4 of the studies. We found no impact of vibroacoustic stimulation on intrauterine or perinatal deaths (RR [Fixed] = 0.98, 95% CI: 0.46–2.10 [NS]; RR [Random] = 0.99, 95% CI: 0.46–2.16 [NS]).

##### Conclusion

The benefits of using fetal vibroacoustic stimulation adjunctively with NSTs must be weighed with respect to the effect on the predictive reliability of the tests and the safety of the procedure. Vibroacoustic stimulation appears to offer maternal, fetal, and clinical benefits by decreasing the incidence of non-reactive cardiotocography and reducing the testing time. Although there are several RCTs and a Cochrane review evaluating vibroacoustic stimulation during pregnancy (Grade B evidence), the non-significant impact on perinatal mortality does not support including this intervention presently. Further RCTs are needed to determine optimal intensity, frequency, duration and position of vibroacoustic stimulation, as well as efficacy, predictive reliability, safety (in terms of fetal hearing impairment and neurological development) and perinatal outcome.

#### Amniotic fluid volume assessment

##### Background

Amniotic fluid protects and supports the fetus during pregnancy. Both low amniotic fluid (oligohydramnios) and high amniotic fluid (polyhydramnios) are abnormal and potentially place the fetus at risk of adverse outcomes. Oligohydramnios can be associated with maternal conditions including pre-eclampsia, or with placental membrane rupture, fetal growth restriction, post-term pregnancy, fetal kidney problems, or fetal or placental abnormalities [105,106]. Polyhydramnios is frequently associated with maternal diabetes mellitus, maternal cardiac problems, twin-twin transfusion syndrome, or fetal or placental malformations, but in 50–60% of cases, it is idiopathic [107-110]. Until ultrasound became available, the invasive nature of amniotic fluid testing limited its clinical usefulness [111]. Using ultrasound, multiple methods for measuring amniotic fluid have been developed and are used to identify at-risk pregnancies. The amniotic fluid index (AFI) is a measure of the amount of amniotic fluid which is calculated by summing centimeters of depth of 4 different pockets of fluid; alternatively, the single deepest vertical pocket or maximum pool depth may be used. At term, many clinicians will induce labour or perform Caesarean section after diagnosis of decreased amniotic fluid volume to prevent an adverse pregnancy outcome. However, using assessments of amniotic fluid volume to predict fetal complications is controversial, and the utility of amniotic fluid assessment is different when used prior to versus after the onset of labour. Here, we examine the potential for assessment of amniotic fluid volume, or interventions to achieve normal amniotic fluid volume, to effectively detect high-risk pregnancy or fetal distress and subsequent adverse perinatal outcomes. We also examined the impact of interventions to achieve normal amniotic fluid volume on perinatal outcomes

##### Literature-based evidence

Our literature search identified two Cochrane reviews, one other review, and 15 other interventional or observational trials of amniotic fluid assessment to identify polyhydramnios or oligohydramnios that reported perinatal outcomes (Table 12 and Table 13).

**Table 12:** Impact of amniotic fluid assessment for polyhydramnios on stillbirth and perinatal mortality

**Source**	**Location and Type of Study**	**Intervention**	**Stillbirths/Perinatal Outcomes**
***Reviews and meta-analyses***			

Magann et al. 2007 [107]	USA, China Systematic review. 7 studies included. N = 3 studies of idiopathic polyhydramnios, N = 4 studies of polyhydramnios that adjusted for congenital anomalies.	Assessed the association of idiopathic polyhydramnios on perinatal outcomes.	PMR: 2-fold to 5-fold increase in risk in polyhydramnios vs. normal AFI groups, respectively.

***Observational studies***			

Mazor et al. 1996 [112]	Israel. Cohort study. Singleton pregnancies (N = 4211) with intact membranes and pre-term delivery (< 37 wks).	Compared the effect in the group with increased amniotic fluid volume (exposed) vs. normal amniotic fluid volume (unexposed) by sonographic assessment.	PMR: OR = 5.8 (95% CI: 3.68–9.11). Intrapartum morbidity: OR = 2.8 (95% CI: 1.94–4.03). Polyhydramnios was an independent predictor of perinatal mortality and intrapartum morbidity.

Dashe et al. 2002 [113]	USA. Retrospective cohort study. N = 672 singleton pregnancies with hydramnios categorised as mild, moderate, or severe based on greatest amniotic fluid index of 25.0-29.9 cm, 30.0-34.9 cm, or 35.0 cm or more, respectively.	To characterise the prevalence and ultrasound detection of fetal anomalies in pregnancies with hydramnios, and to estimate anomaly and aneuploidy risks when no sonographic abnormality is noted.	77 (11%) of neonates had one or more anomalies. Fetal death rate: 4%; 60%; of these had anomalies.

Erez et al. 2005 [114]	Israel. Retrospective logistic regression analysis. N = 192 SGA neonates with polyhydramnios, N = 5,515 SGA neonates with normal amniotic fluid, N = 3,714 appropriate for gestational age (AGA) neonates with polyhydramnios, N = 83,763 AGA.	Assessed the impact of combined SGA and polyhydramnios on perinatal mortality.	PMR: OR = 20.55 (95% CI: 12.6–33.4) comparing SGA+polyhydramnios to AGA fetuses with normal AFI

**Table 13:** Impact of amniotic fluid volume assessment for oligohydramnios and associated interventions on stillbirth and perinatal outcomes

**Source**	**Location and Type of Study**	**Intervention**	**Stillbirths/Perinatal Outcomes**
***Reviews and meta-analyses***			

Hofmeyr et al. 2002 [126]	Japan, USA. Review (Cochrane). 2 studies included (N = 78 women).	Assessed the impact of improved maternal hydration (drinking 2 litres water or intravenous fluids) on amniotic fluid volume and subsequent perinatal outcomes in women with oligohydramnios and normal amniotic fluid volume.	Increased amniotic fluid volume after hydration (women with oligohydramnios): weighted mean difference (WMD) = 2.01 (95% CI: 1.43–2.60) Increased amniotic fluid volume after hydration (women with normal amniotic fluid volume): WMD = 4.5 (95% CI: 2.92–6.08) IV hypotonic hydration (women with olighydramnios): increased amniotic fluid volume=WMD 2.3 (95% CI: 1.36–3.24) Isotonic intravenous hydration: [NS]

Nabhan et al. 2008 [121]	UK, USA. Review (Cochrane). 4 RCTs included (N = 3125 women).	Compared the predictive value of AFI (intervention) versus single-deepest vertical pocket (comparison) methods of amniotic fluid volume assessment in anticipating adverse perinatal outcomes.	Fetal acidaemia: [NS] Presence of meconium: [NS] Apgar < 7 at 5 min: [NS] Caesarean section: [NS] Diagnosis of oligohydramnios: RR[Random] = 2.33 (95% CI: 1.67–3.24) Induction of labour: RR[Fixed] = 2.10 (95% CI: 1.60–2.76) Caesarean for fetal distress: RR[Fixed] = 1.45 (95% CI: 1.07–1.97)

***Intervention studies***			

Alfirevic et al. 1997 [123]	UK. Liverpool Women's Hospital. RCT. Singleton, uncomplicated pregnancies (N = 500) with gestational age ≥ 290 days.	Compared the impact of fetal monitoring by either AFI and computerised cardiotocography (intervention), or maximum pool depth and computerised cardiotocography (controls).	PMR: 0/250 in both groups [NS]

Chauhan et al. 1995 [125]	USA. RCT. Pregnant women 26–42 wks' gestation in early labour.	Compared impact on perinatal outcomes of AFI on admission during early labour (intervention) vs. no AFI (controls).	Caesarean section for fetal distress: RR = 1.3 (95% CI: 1.1–1.7, P = 0.02). [29/447 vs. 14/436 in intervention vs. control groups, respectively.] LBW, macrosomia, Apgar <7, and admissions to the neonatal intensive care unit: [NS].

Oral et al. 1999 [124]	Turkey. RCT. Singleton, uncomplicated pregnancies (N = 101) of gestational age ≥ 290 days.	Compared the impact of either AFI and computerised cardiotocography (intervention) vs. maximal vertical pocket and computerised cardiotocography (controls). Electronic fetal heart monitoring was performed in all patients.	PMR: Maximal amniotic fluid vertical pocket appeared to be slightly better than AFI for identifying the post-term pregnancy at risk for abnormal perinatal outcome.

***Observational studies***			

Anandakumar et al. 1993 [115]	Singapore. National University Hospital. Prospective cohort study. High-risk pregnant women (N = 565).	To evaluate the role of the AFI, used along with NST and fetal acoustic stimulation test, when required, in prediction of adverse pregnancy outcome.	PMR: 6/25, 4 in very low AFI (<5 cm) group (3/4 had reactive NST <7 days before death, P < 0.001 after controlling for NST results).

Baron et al. 1995 [117]	USA. Prospective cohort study. Pregnant women > 26 wks gestation who had an intrapartum AFI measurement.	Compared rates of adverse fetal and neonatal outcomes in women diagnosed with oligohydramnios via AFI (cases) vs. women with normal AFI (controls).	Meconium staining: RR = 0.67 (95% CI: 0.49–0.92) in cases vs. controls, respectively. Variable decelerations: RR = 1.44 (95% CI: 1.12–1.87) in cases vs. controls, respectively. C-section for fetal distress: RR = 6.83 (95% CI: 1.55–30.4). cases vs. controls, respectively. Neonatal complications: No difference between groups. Sensitivity and specificity of oligohydramnios diagnosis for Caesarean delivery for fetal distress: 78% and 74%, respectively.

Kreiser et al. 2001 [180]	USA. Retrospective study. Low-risk singleton pregnancies (N = 150) > 30 wks' gestation with decreased AFI. Pregnancies (N = 57) with very low AFI (≤ 5 cm); N = 93 with borderline AFI (>5 cm but < 2.5^th ^percentile).	Compared the impact in pregnancies with low AFI (intervention) vs. those with borderline AFI (controls).	PMR: 0 in both groups [NS]

Locatelli et al. 2004 [116]	Italy. Prospective study. Uncomplicated, singleton pregnant women (N = 3050) with a non-anomalous fetus reaching 40 wks' gestation recruited from 1997–2000. All women underwent semi-weekly monitoring of AFI until delivery. Oligohydramnios (N = 341).	Compared the rate of oligohydramnios in gestations with adverse perinatal outcome, including 5-min Apgar score < 7; umbilical artery pH < 7.0; Caesarean section for fetal distress; or fetal death (cases) vs. favorable outcome (controls).	Oligohydramnios: 33/167 (19.8%) vs. 308/2883 (10.7%) in cases and controls, respectively; P = 0.001).

Morris et al. 2003 [120]	UK. University teaching hospital. Prospective, double-blind cohort study. Pregnant women (N = 1584) ≥40 wks of gestation were subjected to ultrasound assessment.	To compare predictive ability single ultrasound scan to detect a single deepest pool of AFI<2 cm (exposed) vs. AFI<5 cm (unexposed) in anticipating subsequent adverse pregnancy outcome.	PMR: 0 in both groups. An AFI <5 cm but not a single deepest pool <2 cm was significantly associated with birth asphyxia or meconium aspiration. Sensitivity of AFI < 5 cm for major adverse outcome: 28.6%

Myles et al. 1992 [118]	USA. Prospective cohort study. N = 218 pregnant women on whom AFI was performed (N = 125 with greater volume in upper quadrants; N = 93 with greater volume in lower quadrants).	Assessed the predictive value of distribution of amniotic fluid measured by the 4-quadrant method, comparing perinatal outcomes among women with greater amniotic fluid volume in upper quadrants (intervention) vs. lower quadrants (comparison).	Meconium staining: 32.8% vs. 9.7% in intervention vs. comparison groups, respectively (P < 0.0001). 1-min Apgar <7: 12.0% vs. 2.2% in intervention vs. comparison groups, respectively (P < 0.007). Umbilical arterial pH<7.20: 29.6% vs. 8.9% in intervention vs. comparison groups, respectively (P < 0.0105). Umbilical venous pH <7.20: 8.9 vs. 0% in intervention vs. comparison groups, respectively (P < 0.0398).

Sherer et al. 1996 [181]	USA. Retrospective database study. N = 352 nonhypertensive, nondiabetic pregnant women delivering at < 32 wks' gestation with amniotic fluid measurement performed as part of BPP <24 hours before delivery.	Assessed association of low AFI with fetal movements.	Low AFI associated with reduced fetal movements (P < 0.0001). Higher incidence of chorioamnionitis in patients with no fetal movements (P < 0.005)

Shoham et al. 2001 [182]	Israel. Matched case-control study. N = 368 women with gestational diabetes mellitus (N = 194 with polyhydramnios, AFI>25 cm; N = 184 women with normal AFI) under strict metabolic control enrolled from 1995–1996.	To determine whether gestational diabetes (GDM) complicated with hydramnios is associated with higher rates of perinatal morbidity and mortality than those with normal amniotic fluid (AFI).	No significant differences in rates of antepartum fetal death.

Youssef et al. 1993 [119]	Egypt. Observational study. Fetuses (N = 174) within one wk of delivery.	Compared the impact of the single largest vertical pocket (oligohydramnios = depth < 1 cm) (study group) vs. the 4-quadrant amniotic fluid index (oligohydramnios ≤5 cm) (controls).	The AFI was more sensitive in predicting mortality (87.5%) and the following measures of perinatal morbidity: low 5-minute Apgar score (88.8%), fetal distress during labour (86.6%), meconium-stained amniotic fluid (63.6%), and the presence of fetal growth restriction (79.4%).

Polyhydramnios

Several observational studies explored the association of polyhydramnios with perinatal mortality, with inconsistent findings. Several observational studies [112] observed that polyhydroamnios was an independent risk factor for both perinatal mortality and intrapartum morbidity ***[LOE: 2-]***. A retrospective cohort study by Dashe et al. [113] documented a fetal death rate of 4% in pregnancies with polyhydramnios diagnosed by AFI, of which 40% were unrelated to fetal malformations ***[LOE: 2-]***. Magann et al. conducted a review of idiopathic polyhydramnios [107] including 4 studies that evaluated perinatal mortality with polyhydramnios after correcting for congenital anomalies. In the larger studies, idiopathic polyhyramnios was associated with macrosomia and a statistically significant 2- to 5-fold increase in the risk of perinatal mortality ***[LOE: 2+] ***(Additional file 14). A retrospective logistic regression analysis of SGA and appropriate-weight fetuses with and without polyhydramnios determined that the combination of SGA and polyhydramnios was an independent risk factor for perinatal mortality compared to normal weight-for-age infants with normal AFI values (OR = 20.55; CI: 12.6–33.4) [114]. The association may not apply to certain subsets of high-risk pregnancies, as a similar study among women with gestational diabetes who had AFI performed antenatally found no elevated risk of perinatal morbidity or mortality among pregnancies complicated by polyhydramnios compared to gestational diabetics without polyhydramnios ***[LOE: 2-]***. No intervention studies subsequent to diagnosis of polyhydramnios were identified.

Oligohydramnios

Observational studies of oligohydramnios cases demonstrated a consistent elevated risk of poor perinatal outcomes. Anandakumar et al. [115] studied how AFI used with non-stress cardiotocography and fetal acoustic stimulation for non-reactive NSTs might predict adverse pregnancy outcome in high-risk pregnancies (N = 565 women). Of the 4 perinatal deaths in the group with low AFI, 3 had had a reactive NST within 7 days of fetal death ***[LOE: 2-]***. In Italy, Locatelli et al. [116] reported rates of oligohydramnios twice as high in pregnancies with poor perinatal outcome than pregnancies with no adverse outcome ***[LOE: 2-]***. However, oligohydramnios identified during the intrapartum period did not appear associated with adverse perinatal outcome in high-risk pregnancies: Baron et al [117] observed that women with low AFI were more likely to have Caesarean section for fetal distress than women with normal AFI, but the rates of neonatal complications were similar ***[LOE: 2+]***.

One study found that in addition to the absolute volume of AFI, the distribution of amniotic fluid by intrauterine quadrants is predictive of fetal outcome. Myles et al. [118] observed higher rates of meconium staining, poor 1-minute Apgar scores, fetal heart decelerations, and fetal acidaemia in pregnancies where fluid volume was higher in the upper quadrants than in pregnancies where fluid volume was higher in lower quadrants ***[LOE: 2+]***.

A number of observational studies, RCTs and a systematic review compared AFI measurement to other methods of measuring amniotic fluid in ability to predict poor perinatal outcome. In an observational study in Egypt, Youssef et al. [119] (N = 174 women) found that the AFI was more sensitive than the single largest vertical pocket measurement in predicting perinatal mortality and multiple measures of perinatal morbidity. Morris et al. [120] documented similar findings, but the sensitivity of AFI < 5 cm for major adverse fetal outcome was only 28 percent. A Cochrane review [121] compared the evidence for the predictive value of 2 methods of amniotic fluid assessment (AFI versus single deepest vertical pocket) on adverse pregnancy outcomes (Additional file 15). Compiling the evidence from available RCTs (4 trials, N = 3125 women), the authors found that AFI versus single deepest vertical pocket led to significantly more cases of oligohydramnios being diagnosed (RR [Random] = 2.33, 95% CI: 1.67–3.24), more frequent labour induction (RR [Fixed] = 2.10, 95% CI: 1.60–2.76) and higher rates of Caesarean delivery for fetal distress (RR [Fixed] = 1.45, 95% CI: 1.07–1.97). They recommended that the single deepest vertical pocket measurement be used because AFI resulted in increased diagnoses of oligohydramnios and rates of labour induction with no improvement in perinatal outcomes compared to single deepest vertical pocket, suggesting better predictive value of single deepest vertical pocket. However, others have expressed concern that inter-observer reliability is lower with single deepest vertical pocket measurement (kappa = 0.33) compared to AFI (kappa = 0.72) [122]. An RCT by Alfirevic et al [123] randomised women to fetal monitoring by either AFI with computerised cardiotocography, or maximum pool depth determined by computerised cardiotocography, but found no statistically significant difference in perinatal outcome in the 2 groups. Another RCT by Oral et al [124] (N = 101 pregnancies) compared fetal monitoring (with cardiotocography) by either AFI or maximal vertical pocket. Measurement of maximal amniotic fluid vertical pocket proved slightly better than AFI in identifying post-term pregnancies with abnormal perinatal outcomes.

Only one intervention RCT compared AFI to no amniotic fluid assessment. Comparing AFI on admission (intervention) to no AFI (controls), Chauhan et al [125] found that women in the intervention group with a diagnosis of low AFI (measured as ≤ 5 cm or ≤ 5^th ^percentile) were no more likely to have Caesarean section for fetal distress, neonatal acidosis, or Apgar score < 7 at 5 minutes than untested controls or women with normal AFI results; no perinatal mortality statistics were reported ***[LOE: 1+]***.

One intervention strategy to improve perinatal outcomes is to augment amniotic fluid volume in cases of oligohydroamnios. Hofmeyr and Gülmezoglu [126] undertook a systematic review of RCTs (2 trials, N = 78 women) assessing the impact of maternal hydration status (requesting that women drink 2 litres of water prior to a repeat ultrasound) on AFI (Additional file 16). In women with and without oligohydramnios, drinking water was associated with an increase in amniotic volume (WMD for women with oligohydramnios = 2.01, 95% CI: 1.43–2.60; WMD for women with normal AFI = 4.5, 95% CI: 2.92–6.08). Intravenous hypotonic hydration in women with oligohydramnios was associated with an increase in amniotic fluid volume (WMD = 2.3, 95% CI: 1.36–3.24), but isotonic intravenous hydration had no measurable effect.

##### Conclusion

Polyhydramnios is a clear risk factor for perinatal mortality, whether associated with congenital malformations, placental insufficiency, or of idiopathic origin. However, no studies reported the impact of interventions subsequent to a diagnosis of polyhydramnios after amniotic fluid assessment, so the impact on stillbirth of amniotic fluid screening for polyhydramnios remains unclear. Although robust RCTs are limited, very low AFI values (oligohydramnios) are frequently associated with poor pregnancy outcomes, and in these cases a reactive NST loses its usual reassuring value (Grade C evidence). Where feasible, amniotic fluid volume estimation may be helpful for identifying severe oligohydramnios, but further research is needed to document subsequent intervention and perinatal mortality outcomes to determine the cost-benefit ratio of utilizing amniotic fluid assessment procedures. Amniotic fluid assessment is complicated by high variability of sequential measurements and use of different measurement methods, which can compromise the accuracy of the test. No particular method of amniotic fluid volume assessment appears superior to another [123,124], though using maximum vertical pool depth rather than AFI appears to limit unnecessary inductions of labour and Caesarean section by reducing diagnoses of oligohydramnios. There is a need for further research to test the impact of interventions to prevent or treat oligohydroamnios, particularly in the antepartum period with intact membranes, on perinatal outcomes.

#### Home versus hospital bed rest and monitoring for high-risk pregnancies

##### Background

Women with high-risk pregnancies, especially multiple pregnancies and pregnancies complicated by hypertensive disorders, are frequently admitted to hospital for bed rest and monitoring. However, in some instances, more limited monitoring in facilities and home-based bed rest or reduced physical activity may be as effective as hospital-based monitoring. Home-based activity modification, accompanied by outpatient surveillance and hospital admissions only for complications, would offer cost savings over hospital admission, reduced burden on hospital resources and personnel, and reduced disruption to the life of the mother posed by lengthy hospital stays.

##### Literature-based evidence

The literature search identified two Cochrane reviews and two other interventional studies comparing home-based versus hospital-based methods of care for high-risk pregnancies (Table 14). The first Cochrane review [127] assessed the effectiveness, in management of multiple gestations, of hospital-based bed rest versus home bed rest (reduced physical activity) with admission only for complications (6 RCTs, N>600 women, N>1400 infants) (Additional file 17). Hospital bed rest had no advantage compared to domiciliary bed rest in reducing perinatal mortality (OR = 0.89, 95% CI: 0.43–1.85), but birth weights were slightly higher in the hospitalised group. In the subset of trials of uncomplicated twin [128] and triplet [129] pregnancies, there was no advantage of hospital-based bed rest compared to non-hospitalised patients in preventing stillbirths (OR = 0.82, 95% CI: 0.38–1.77; and OR = 6.69, 95% CI: 0.13–338.79; for twin and triplet pregnancies, respectively) ***[LOE: 1++]***.

**Table 14:** Impact of home versus hospital-based bed rest and monitoring in high-risk pregnancy on stillbirth and perinatal mortality

**Source**	**Location and Type of Study**	**Intervention**	**Stillbirths/Perinatal Outcomes**
***Reviews and meta-analyses***			

Crowther 2001 [127]	Zimbabwe, Finland, Australia. Meta-analysis (Cochrane). 6 RCTs included (N = 1431 women).	Compared pregnancy outcomes among women with a multiple pregnancy and their babies who were offered bed rest in hospital (intervention) vs. admission to hospital only if complications occurred (controls).	SBR: OR = 0.89 (95% CI: 0.43–1.85) [NS]. [14/698 vs. 16/733 in intervention and control groups, respectively]. PMR: OR = 1.14 (95% CI: 0.65–2.01) [NS]. [26/694 vs. 24/733 in intervention and control groups, respectively].

Meher et al. 2005 [130]	Zimbabwe. Cochrane review. 1 RCT included (N = 218 women).	To assess the effects on the mother and the baby of some bed rest in hospital (intervention) vs. routine activity at home (controls) for primary treatment of hypertension during pregnancy.	SBR: RR = 4.91 (95% CI: 0.24–101.10) [NS]. [2/110 vs. 0/108 in intervention and control groups, respectively]. PMR: RR = 1.96 (95% CI: 0.18–21.34) [NS]. [2/110 vs. 1/108 in intervention and control groups, respectively].

***Intervention studies***			

Monincx et al. 1997 [131]	The Netherlands (Amsterdam). The Academic Medical Centre. RCT. N = 150 women recruited between September 1992 and June 1994 (N = 76 intervention group, N = 74 controls).	To compare the outcomes of high-risk pregnancy monitored antenatally at home via domiciliary care (intervention) vs. hospital admission (controls).	SBR: 1/77 (1%) vs. 0/74 in intervention and control groups, respectively. PMR: 1/77 vs. 1/74 in intervention and control groups, respectively.

[No authors listed] 1990 [132]	China (Shanhai). 6 hospitals. RCT. Women (N = 13006) of at least 30 wks' gestation (N = 6,506 intervention group, N = 6,500 controls).	Compared the impact on PMR of self-monitoring at home (intervention) vs. controls (without self-monitoring).	PMR: 6.30% vs. 10.92% in intervention and control groups, respectively (statistically significant). Fetal death (miscarriage + SB): 3.23% vs. 6.34% in intervention and control groups, respectively (statistically significant).

Another Cochrane review [130] evaluated the impact of different degrees of bed rest compared with routine activity, as well as the effects of hospital-based versus home-based bed rest, in hypertensive pregnant women both with and without proteinuria (4 RCTs, N = 449 women) (Additional file 18). Three studies were of good quality, but only 1 reported stillbirth as outcome, reporting no impact of hospital-based bed rest versus routine activity at home on stillbirth (RR = 4.91, 95% CI: 0.24–101.10). Only 1 RCT (N = 218 women) of the 2 studies comparing hospital-based bed rest with routine activity at home in cases of non-proteinuric hypertension reported stillbirth and neonatal death as a combined measure. There was no evidence of impact of hospital-based bed rest on this composite measure (RR = 1.96, 95% CI: 0.18–21.34), though they did report a reduced risk of severe hypertension (RR = 0.58, 95% CI: 0.38–0.89) and a borderline reduction in risk of pre-term birth (RR = 0.53, 95% CI: 0.29–0.99) compared to normal domiciliary activity ***[LOE: 1++]***

In The Netherlands, an RCT [131] compared domiciliary care to hospital-based care in high-risk pregnant women (N = 150). There was 1 perinatal death in each group (1/77 versus 1/74 in home- versus hospital-based groups, respectively), and the only stillbirth occurred in the domicilary care group (1/77 versus 0/74 in home- versus hospital-based groups, respectively); the sample was too small to assess statistical significance ***[LOE: 1-]***. A large RCT from China [132] (N = 13,006 women) compared a protocol of self-monitoring at home to a control group managed at hospitals. Both perinatal mortality and fetal deaths were lower among the self-monitoring group (perinatal mortality: 6.30% versus 10.92% in self-monitoring versus control groups, respectively, P < 0.05; fetal deaths: 3.23% versus 6.34%, respectively, P < 0.05).

##### Conclusion

The limited evidence available (Grade D evidence) suggests that home-based care for certain subsets of women with uncomplicated high-risk pregnancy has no apparent disadvantage compared with hospital-based bed rest and monitoring in terms of impact on stillbirths and perinatal mortality. The number of studies on this subject is small, however, and limited to the conditions of multiple gestation and hypertensive disorders of pregnancy, exclusively in high-resource settings. As one RCT above indicated, bed rest in hospital for non-proteinuric hypertension may be superior to home-based care [133], but insufficient evidence exists to recommend hospitalization in this case, as the study was underpowered. The Cochrane review found that in multiple pregnancy, bed rest (in hospital) appears to confer little advantage over no modification of physical activity; bed rest had no impact on rates of perinatal mortality, stillbirth, or pre-term birth, but may improve fetal growth [127]. The potential cost savings and increased convenience to women of home-based bed rest and monitoring indicates that further large studies of the efficacy of bed rest as an intervention as well as monitoring at home versus in hospital are needed, particularly studies that include economic analyses of costs to mothers and hospitals.

#### In-hospital fetal surveillance units

##### Background

A fetal surveillance unit provides a wide range of maternal and fetal diagnostic tests, often on an outpatient basis, of particular benefit in identifying and monitoring high-risk pregnancies. Antenatal fetal surveillance protocols, which may include multiple surveillance methods at a specified frequency, have the potential to impact perinatal mortality, neonatal morbidity, birth weight in cases of fetal growth problems, rates of prematurity, and length of hospital admission. Assessment of fetal condition can usually be performed on an outpatient basis, with admission reserved for delivery, which is usually less disruptive to the pregnant woman's family [134]. Specially designated antenatal surveillance units that offer predominantly outpatient services also offer operating cost savings compared with standard hospital admission.

##### Literature-based evidence

Our literature search identified 1 Cochrane review, 1 Cochrane protocol and 2 other intervention studies (Table 15). A Cochrane review [135] evaluated the use of antenatal outpatient day care units as an alternative to inpatient care for women with complicated pregnancy (1 RCT, N = 54 women) [136] (Additional file 19). Day care unit assessment for non-proteinuric hypertension reduced subsequent inpatient stay (difference in mean stay: 4.0 days; 95% CI: 2.1–5.9 days). The rate of induction of labour was much higher in the hospital-based inpatient care group compared to the day care unit group (OR = 4.9, 95% CI: 1.6–13.8). There were no perinatal deaths in either group ***[LOE: 1+]***. The Cochrane protocol, for a review currently in progress, proposes to assess the impact of different specified regimens of fetal surveillance for impaired fetal growth on maternal and perinatal outcomes and length of hospital admission [137]. The 2 intervention studies adopted different approaches. Soothill et al [134] conducted a before-after intervention study comparing the number and length of antenatal admissions in the 5 months after the opening of a perinatal care unit providing largely outpatient-based services to records from the 6 months prior to the opening. They found no significant change in stillbirth rate (6/1294 versus 8/1372 after versus before, respectively; rate difference (RD) = 0.0012, 95% CI: -0.0043–0.0067) ***[LOE: 2+]***, suggesting that fetal surveillance on an outpatient basis was equally effective as hospital admission in managing high-risk pregnancy. Menzies et al. [138] performed a similar study of all women admitted with pre-eclampsia to a tertiary-level perinatal unit before and after introducing standardised assessment and surveillance, and reported that perinatal outcomes did not change ***[LOE: 2-]***.

**Table 15:** Impact of a fetal surveillance unit on stillbirth and perinatal outcomes

**Source**	**Location and Type of Study**	**Intervention**	**Stillbirths/Perinatal Outcomes**
***Reviews and meta-analyses***			

Kröner et al. 2001 [135]	UK. Cochrane review. 1 RCT included (N = 54 women).	Compared the impact on perinatal mortality of antenatal day care units (intervention) vs. inpatient care (controls) for women with complicated pregnancy.	PMR: OR not estimable. [0/30 vs. 0/24 in intervention and control groups, respectively].

***Intervention studies***			

Menzies et al. 2007 [138]	Canada. Tertiary perinatal unit. Before-after design. Pregnant women (N = 700) admitted to hospital with pre-eclampsia.	Compared the impact after (intervention) and before (controls) introducing standardised assessment and surveillance for pre-eclampsia.	SBR: OR = 1.47 (95% CI: 0.50–4.34, P = 0.602) [NS] 10/405 vs. 5/295 in the intervention and control groups, respectively.

Soothill et al. 1991 [134]	UK (London). Kings College. Before-after design. Pregnancies (N = 2666).	Compared the number and length of antenatal admissions for 6 months before compared to 5 months after the opening of a fetal surveillance unit.	SB: 6/1294 vs. 8/1372 in before and after periods, respectively; (P = 0.336) [NS]

##### Conclusion

If patients could be consistently monitored on an outpatient basis, obstetricians would be more willing to admit patients only for delivery, and to perform more fetal assessments and maternal tests as outpatient services. Outpatient fetal surveillance offers benefits in terms of cost and convenience, and could improve health facility organisation, and improved record-keeping would streamline obstetric and perinatal audit, data quality for research, and teaching opportunities [135]. However, the evidence base for the benefits of outpatient surveillance of high-risk pregnancies is relatively limited, as the single Cochrane review on the subject included 1 small RCT that reported no perinatal deaths (Grade C evidence). The reduced rates of induction of labour are of interest and require further trials reporting labour induction rates with sufficient power to detect differences in subsequent perinatal outcomes. At this time, even in high-resource settings, in-hospital fetal surveillance units cannot be recommended as an intervention to prevent stillbirths.

### Monitoring in labour

#### Use of the partograph

##### Background

A partograph, alternatively called a partogram, is a simple pre-printed paper form on which midwives and obstetricians record labour observations. The tool provides a continuous pictorial overview of the progress of labour, while monitoring maternal and fetal well-being. The partograph distinguishes between the latent and active phases of labour. The active labour section has 2 straight lines called the alert and action lines. The alert line reflects a modification of the mean rate of cervical dilatation of the slowest 10% of primigravid women in the active phase of labour (1 cm per hour). Slower progress than this crosses the alert line on the partograph, which may prompt initiation of the process of transfer to a facility with emergency obstetric capacity in preparation for intervention for prolonged labour. Depending on the partograph version, the 'action line' is 2 to 4 hours to the right of the alert line. Labour crossing this line suggests primary inefficient uterine activity and prompts immediate appropriate management of slow progress of labour, usually via amniotomy, oxytocin infusion, or both.

Some evidence suggests that midwives and physicians find the partograph practical in terms of ease of use, time resourcefulness, continuity of care and educational assistance [139], which may contribute to positive maternal and fetal outcomes. Partographs are also inexpensive and relatively simple to use, making their use attractive in low-resource settings where other intrapartum monitoring technologies are unavailable or prohibitively expensive. In higher-resource settings, the partograph can be implemented alongside other fetal surveillance tests such as cardiotocography to provide more information for decision-making. However, some practitioners view the partograph as inappropriately restrictive and formulaic, prompting intervention prematurely [139], factors which could impact both clinical and maternal psychological outcomes. Various versions of the partograph are marked by different slope and position of the action line, which is likely to impact labour augmentation interventions, Caesarean section rates, and maternal satisfaction.

##### Literature-based evidence

We identified 1 Cochrane review including 5 RCTs and quasi-RCTs (N = 6963 women), and 3 intervention/observational studies (Table 16). The Cochrane review [140] compared the impact of use of the partograph in comparison with no partograph, as well as different versions of the partograph, for monitoring the progress of spontaneous labour at term (Additional file 20). None of the included studies reported intrapartum stillbirth rates, though several reported neonatal outcomes. The randomised controlled trials (RCTs) in the review that compared partograph use to no partograph use (2 trials; N = 1590 women)[141,142] showed no impact of partograph use on Caesarean section rate (risk ratio (RR) = 0.64, 95% confidence interval (CI): 0.24–1.70), instrumental vaginal delivery (RR = 1.00, 95% CI: 0.85–1.17) or Apgar score < 7 at 5 minutes (RR = 0.77, 95% CI: 0.29–2.06).

**Table 16:** Impact of use of the partograph in stillbirth and perinatal outcomes

**Source**	**Location and Type of Study**	**Intervention**	**Stillbirths/Perinatal Outcomes**
***Reviews and meta-analyses***			

Lavender et al. 2008 [140]	England and South Africa. Meta-analysis (Cochrane). 5 RCTs included; 3 reported serious neonatal morbidity or PMR. N = 6963 women.	Assessed the use of partograph vs. no partograph; and compared impact of different versions of partograph (e.g. partogram with 2-hr, 3-hr, 4-hr, or no action line).	Serious neonatal morbidity or PMR: OR not estimable. [0/1805 vs. 0/1796 in the 2-hour vs. 4-hour action line groups, respectively].

***Intervention studies***			

Fahdhy 2005 [148]	Indonesia (Medan City). Cluster RCT. 20 midwives in maternity homes. N = 626 pregnant women with vertex presentations (N = 304 intervention, N = 322 controls).	Assessed the impact of the use of the WHO partograph by trained midwives (intervention) vs. standard midwifery care without partograph (controls). 92% of partographs correctly completed; N = 71 had graph beyond alert line. 42/71 referred to hospital.	Fetal death: adj. OR = 0.62 (95% CI: 0.17–2.19) [NS] [5/304 vs. 7/302 in intervention vs. control groups, respectively.] END: adj. OR = 0.70 (0.16–3.11) [NS] [3/304 vs. 7/302 in intervention vs. control groups, respectively.] Significant decreases in obstructed labour, oxytocin use:, Apgar <7 at 1 min: No difference in Caesarean section rate, Apgar <7 at 5 min, or prolonged labour.

Lennox 1998 [147]	Indonesia, Thailand, Malaysia. Hospital-based study. Multicentre. Before-after study. 8 hospitals. N = 1740 breech presentation pregnancies (N = 817 after, N = 923 before).	Assessed the impact of use of the partograph with an agreed labour-management protocol on perinatal outcomes.	Intrapartum SB (breech): 1.1% vs. 1.9% after vs. before, respectively. [NS] Prolonged labour: Significant reduction with partograph (P < 0.05)

WHO 1994 [146]	Indonesia, Thailand, Malaysia. Hospital-based study. Multicentre. Quasi-RCT. 8 hospitals. N = 35,484 women.	Assessed the impact of use of the partograph with an agreed labour-management protocol on perinatal outcomes.	Intrapartum SB: 0.3% vs. 0.5% in intervention vs. control groups, respectively. Prolonged labour: 3.4% vs. 6.4% in intervention vs. control groups, respectively. Oxytocin augmentation: 9.1% vs. 20.7% in intervention vs. control groups, respectively. Emergency Caesarean sections: 8.3% vs. 9.9% in intervention vs. control groups, respectively.

The Cochrane review [140] also compared the impact of different versions of the partograph with different action line placement on labour outcomes. Two RCTs, both conducted in high-resource settings, compared different partograph versions with the action line either 2 hours or 4 hours after the alert line (N = 3601 women) [143,144], and found no difference in Caesarean section rates between labours monitored with the 2 different partographs (RR = 1.06, 95% CI: 0.85–1.32 [NS]). Women in the 2-hour action line group were significantly more likely to receive oxytocin augmentation (RR = 1.14, 95% CI: 1.05–1.22). One RCT, also in a high-resource setting, compared a 2-hour versus a 3-hour action line (N = 617 women)[143]. There was no difference in Caesarean section (RR = 0.78, 95% CI: 0.51–1.18). Maternal satisfaction with care was higher in the 2-hour action line group than the 3-hour action line group (RR = 0.49, 95% CI: 0.27–0.90). There was no difference in neonatal outcomes. One RCT in a high-resource setting compared the impact of partographs with a 3-hour versus a 4-hour action line (N = 613) [143]. The Caesarean section rate was significantly lower in the 4-hour action line group (RR = 1.70, 95% CI: 1.07–2.70) but there was no difference in neonatal outcomes. One RCT in a low-resource setting compared a partograph with no action line (alert line only) to a partograph with an action line (N = 694 women) [145]. The Caesarean section rate was lower in the group with no action line (RR = 0.68, 95% CI: 0.50–0.93), but there was no difference in neonatal outcomes.

The review also pooled the results from 3 RCTs to compare shorter (2-hour action line or alert line only) versus longer time-to-intervention (4-hour action line or alert plus action lines) and found no differences between the groups for Caesarean section rate, Apgar score or instrumental delivery, but early intervention in the low-resource setting reduced the Caesarean section rate.

A large multicentre study [146] compared the impact of partograph use in multiple hospitals in Southeast Asia (N = 35,484 women). The reported stillbirth rate was 0.3% in the group for which the partograph was used, versus 0.5% in the control group; this small difference was not assessed for statistical significance because partograph use was introduced in stages. A sub-study using the same WHO dataset of breech birth management using the partograph [147] found a non-significant decrease in intrapartum stillbirth after the introduction of the partograph compared with before (1.1% versus 1.9% after versus before, respectively).

In Indonesia, Fahdhy et al [148] compared the use of midwives trained to employ the partograph versus standard midwifery care without the partograph. Seventy-one of 304 labours plotted on the partograph progressed beyond the alert line; the study reported significant decreases in obstructed labour, oxytocin use, and Apgar score less than 7 at 1 minute, but there were only non-significant reductions in fetal death and early neonatal death [adjusted OR for fetal death = 0.62, 95% CI: 0.17–2.19 (NS); adjusted OR for neonatal death = 0.70, 95% CI: 0.16–3.11(NS)].

##### Conclusion

Overall, there were no significant differences in maternal or perinatal outcomes with the use of partograph versus no partograph, and no evidence that any particular version of the partograph is better than another in preventing perinatal mortality. Partographs may be comparatively more effective in low-resource settings, as the studies from Africa and Mexico in the Lavender review [140], as well as data from Southeast Asia [146] that showed reduced Caesarean section rates with use of the partograph and early intervention for slow progress of labour. The data from Southeast Asia and Indonesia also showed trends toward improved birth outcomes [146]. Our overall assessment of the grade of evidence of studies of partograph is Grade C. Given the limitations of the studies included and the potential impact of organisational issues, e.g. guidelines on partograph use, a large cluster-RCT in low-resource settings is recommended to compare partograph versus no partograph use, specifically including stillbirths as a reported outcome.

#### Intrapartum cardiotocography with and without pulse oximetry

##### Background

Methods to assess fetal heart rate and levels of oxygenation of fetal blood are monitoring strategies intended to identify early signs of fetal compromise as a result of oxygen shortage, or fetal hypoxia [2]. Severe and prolonged hypoxia is associated with stillbirth, early neonatal death, and long-term physical or mental disability, including cerebral palsy, if the baby survives. Fetal heart rate patterns are classified as either reassuring, nonreassuring or abnormal, considering heart rate baseline, variability, and decelerations. While nonreassuring tests may become reassuring with simple change in maternal position, abnormal fetal heart rate alterations or low blood oxygen levels are a frequent indication for Caesarean or instrumental delivery [149].

In many hospitals in high-income countries including the US and Canada, cardiotocography is widely used to monitor fetal heart rate in labour, though intermittent auscultation (periodic listening to the fetal heart rate using a stethoscope or handheld Doppler device) is occasionally performed. In addition to external cardiotocography, which can be employed continuously or intermittently during labour, internal cardiotocography can be performed by attaching a sensor to the fetal presenting part, usually its head. This can be done only if the membranes are ruptured, as electrode must be embedded in the baby's scalp. Intermittent auscultation via stethoscope or handheld Doppler is more common than electronic fetal monitoring methods in low-resource settings where fetal heart rate monitoring is available.

Currently, cardiotocography is recommended in high-risk pregnancy and labours induced or augmented with oxytocin [150]. Because of the poor ability of cardiotocography alone to detect true fetal distress, the practice of pulse oximetry is thought to provide additional helpful information to corroborate cardiotocographic traces. Pulse oximetry is intended as a follow-up procedure in the presence of a nonassuring cardiotocographic test, and is intended to improve the accuracy of the assessment of fetal well-being in the intrapartum period [151]. In this procedure, a sensor is attached via a clip to the fetus, generally on the scalp, cheek, temple, or back; oxygenation values exceeding 30% are considered reassuring even when a cardiographic trace is nonreassuring [152]. While non-reassuring tests may become reassuring with simple change in maternal position, abnormal fetal heart rate alterations are a frequent indication for Caesarean or instrumental delivery [149].

There is some danger that improper interpretation of cardiotocographic tracings can lead to inappropriate intervention, or false reassurance that delays necessary intervention. There is a need for sensitive and specific methods of using cardiotocography, potentially in conjunction with pulse oximetry, to improve detection of fetal compromise due to hypoxia. Improved detection of hypoxia, primarily by expediting delivery, could improve outcomes and prevent stillbirth in these instances.

##### Literature-based evidence

The literature review identified two Cochrane reviews on intrapartum cardiotocography, one Cochrane review examining pulse oximetry in conjunction with cardiotocography, and two other observational/interventional studies (Table 17). In a large Cochrane review (12 trials, N>37,000 women), Alfirevic and Devane [2] evaluated RCTs and quasi-RCTs comparing continuous cardiotocography in labour (with and without fetal blood sampling) with no fetal monitoring, intermittent auscultation, or intermittent cardiotocography (Additional file 21). Compared to intermittent auscultation, continuous cardiotocography showed no significant difference in overall PMR (11 trials, N = 33,513 women, RR = 0.85, 95% CI: 0.59–1.23), but did significantly reduce the risk of neonatal seizures (RR = 0.50, 95% CI: 0.31–0.80, 9 trials, N = 32,386) although no significant difference was detected in cerebral palsy (RR = 1.74, 95% CI: 0.97–3.11, 2 trials, N = 13,252). Continuous cardiotocography was associated with an increased risk of Caesarean delivery (RR = 1.66, 95% CI: 1.30–2.13, 10 trials, N = 18,761 women) and instrumental vaginal birth (RR = 1.16, 95% CI: 1.01–1.32, 9 trials, N = 18,151 women). Results of subgroup analysis were consistent with overall results presented above, and the addition of fetal blood sampling appeared to have no effect on outcomes ***[LOE: 1+]***. A new Cochrane review assessing cardiotocography versus intermittent auscultation of fetal heart for assessment of fetal well-being is in progress [153].

**Table 17:** Impact of intrapartum cardiotocography with or without pulse oximetry on stillbirth and perinatal outcomes

**Source**	**Location and Type of Study**	**Intervention**	**Stillbirths/Perinatal Outcomes**
***Reviews and meta-analyses***			

East et al. 2007 [149]	USA, Australia, Germany. Meta-analysis (Cochrane). 4 RCTs included (N = 1789).	To compare the effectiveness and safety of fetal pulse oximetry + cardiotocography (intervention) vs. conventional surveillance techniques (cardiotocography only).	Fetal death (miscarriage+SB)/NMR: RR = 0.93 (95% CI: 0.20–4.44) [NS]. [3/942 vs. 3/847 in intervention and control groups, respectively] for gestation from 36 weeks and fetal blood sampling (FBS) not required prior to study entry.

Neilson 2006 [154]	Sweden, Finland, UK, Hong Kong, Netherlands, Singapore. Meta-analysis (Cochrane). 4 RCTs included (N = 9829).	To compare the effects of analysis of fetal ECG waveforms during labour (intervention) vs. alternative methods of fetal monitoring (no ECG) (controls).	PMR: RR = 2.29 (95% CI: 0.59–8.83) [NS]. [6/4953 vs. 2/4876 in intervention and control groups, respectively].

Alfirevic et al. 2006 [2]	Athens, Copenhagen, Denver, Dublin, Australia, Pakistan, USA, Sheffield. Meta-analysis (Cochrane) 11 RCTs included (N = 33,513).	To assess the effectiveness of continuous cardiotocography during labour (intervention) vs. intermittent auscultation (controls).	PMR: RR = 0.85 (95% CI: 0.59–1.23) [NS]. [50/16849 vs. 57/16664 in intervention and control groups, respectively].

***Observational studies***			

Seelbach Gobel 1999 [183]	Germany. Multicentreed study involving 3 obstetric centres. Observational study. N = 400 deliveries monitored by fetal pulse oximetry.	Compared the durations of different fetal arterial oxygen saturations in neonates with a pH < 7.15 vs. ≥ 7.15, base excess < -12 mmol/L vs. > -12 mmol/L in the umbilical artery post partum and in neonates with an Apgar score < 7 vs. ≥ 7.	Duration of low fetal arterial oxygen saturation: significantly longer in neonates with a 1-minute Apgar score <7 vs. ≥ 7, with pH < 7.15 vs. = 7.15 and with base excess < -12 mmol/L vs. ≥ -12 mmol/L. Duration of medium fetal arterial oxygen saturation: no significant differences between the groups. Duration of high fetal arterial oxygen saturation: significantly shorter for children with pH < 7.15 vs. ≥ 7.15 and with base excess < -12 mmol/L vs. ≥ -12 mmol/L; no significant difference in children with Apgar score < 7 vs. ≥ 7. The duration of low fetal arterial oxygen saturation proved to be the best predictor of a decline of scalp pH between 2 fetal scalp blood samples. The pH declined significantly with a longer duration of low fetal arterial oxygen saturation (0.02 per 10 minutes). No decrease of pH by more than 0.05 was observed unless fetal arterial oxygen saturation had remained at ≤ 30% for ≥ 10 minutes.

Stiller et al. 2002 [184]	Switzerland. Test sensitivity and specificity analysis. N = 107 sets of measures.	To determine the sensitivity and specificity for acidosis of intrapartum fetal oxygen saturation measured by reflectance pulse oximetry.	Mean fetal oxygen saturation was 42.8%, over the mean 132 minutes of 107 recordings. Depending on stage and umbilical artery parameter, fetal oxygen saturation cutoffs were 33% to 36%, with sensitivities of 0.67 to 0.8 and specificities of 0.62 to 0.90. Umbilical artery values tended to be less favorable at SpO_2 _levels < 40%; above 40% no unfavorable values were reported.

Another Cochrane review [154] (4 RCTs, N = 9829 women) compared fetal electrocardiogram ST waveform analysis with alternative methods of fetal monitoring during labour, including continuous cardiotocography (Additional file 22). In comparison to continuous cardiotocography alone, the use of adjunctive electrocardiogram analysis reduced the risk of neonatal acidosis at birth (3 trials, N = 8872 women; RR = 0.64, 95% CI: 0.41–1.00, N = 8108 babies) and neonatal encephalopathy (3 RCTs, RR = 0.33, 95% CI: 0.11–0.95). Procedurally, adjunctive electrocardiogram evaluating the ST segment was associated with fewer fetal scalp samples during labour (3 RCTs, RR = 0.76, 95% CI: 0.67–0.86) and fewer instrumental vaginal deliveries (3 RCTs, RR = 0.87, 95% CI: 0.78–0.96), but had no impact on Caesarean section rates or Apgar score < 7 at 5 minutes. Use of another electrocardiogram method, time-interval analysis, showed no benefit other than a trend towards fewer operative deliveries (1 RCT, RR = 0.87, 95% CI: 0.76–1.01); there was no significant increased risk of perinatal death in the electrocardiogram plus cardiotocography group versus the group with cardiotocography alone (RR = 2.29, 95% CI: 0.59–8.83) ***[LOE: 1++]***.

A third Cochrane review [149] assessed RCTs that comparied maternal and fetal outcomes after fetal pulse oximetry was used in labour (with or without concurrent use of cardiotocography or auscultation), compared with cardiotocography alone (Additional file 23). Adjunctive fetal pulse oximetry with cardiotocography was associated with significantly decreased rates of Caesarean section for nonreassuring fetal status compared to cardiotocography alone in groups without fetal blood sampling prior to study entry (RR = 0.68, 95% CI: 0.47–0.99), and in groups with fetal blood sampling prior to study entry (RR = 0.03, 95% CI: 0.00–0.44). Based on 2 trials reporting outcomes, there was no impact of adding fetal pulse oximetry to cardiotocography on fetal/neonatal death (RR = 0.93, 95% CI: 0.20–4.44) ***[LOE: 1++]***.

##### Conclusion

Although there are several studies of fetal pulse oximetry and intrapartum cardiotocography, few reported stillbirth or perinatal mortality as outcomes. The available results show no statistically significant impact on stillbirths or perinatal mortality, whether cardiotocography is used alone or in conjunction with fetal pulse oximetry in labour. Continuous cardiotocography appears associated with increased rates of operative delivery and lower rates of neonatal seizures compared to no or intermittent cardiotocography, but has no demonstrated impact on rates of perinatal mortality acidosis, dystocia, or long-term physical or developmental outcomes. There is currently no evidence of benefit from randomised or quasi-randomised studies for intrapartum cardiotocography alone or in conjunction with electrocardiogram or fetal pulse oximetry in preventing stillbirth.

One drawback of intrapartum cardiotocography is that its poor predictive value of true fetal distress and hypoxia is not sufficiently enhanced by any adjunctive technologies at this time. Pulse oximetry appears safe, though long-term developmental studies have not been performed, but it does not have any clear impact on stillbirths or perinatal mortality [149]. Adjunctive electrocardiogram possibly reduces neonatal encephalopathy, academia, and instrumental delivery, but these findings need additional research to verify. Minimally invasive technologies and tests are needed in addition to intrapartum cardiotocography to more accurately identify intrapartum hypoxia and dystocia.

There are robust studies and Cochrane reviews on fetal pulse oximetry and intrapartum cardiotocography (Grade B evidence). The non-significant impact of cardiotocography with or without fetal pulse oximetry on stillbirth/perinatal mortality in these studies suggests no evidence of benefit. In high-income countries, documented stillbirth rates, particularly stillbirths associated with intrauterine asphyxia, in high-income countries have followed increasing prevalence of cardiotocographic fetal monitoring with Caesarean section for fetal distress [155-157], though this association may be confounded. Electronic fetal monitoring (with access to operative delivery) may an important tool in the arsenal of strategies to prevent stillbirth, though this is unproven. More precise tests for fetal distress and indications for Caesarean section are needed.

## Summary

Although a reasonable number of Cochrane reviews and RCTs were available which assessed the impact of one or more implementation strategies for many screening and monitoring interventions, none of the interventions we reviewed demonstrated convincing evidence of impact on stillbirths or perinatal mortality. A number of studies that did report statistically significant impact on stillbirth or perinatal mortality, or which suggested large magnitude differences between intervention and control/comparison groups, lacked sufficient rigor to justify recommending the routine use of the interventions evaluated.

The evidence for all interventions reviewed in this paper is summarised in Table 18. Of the range of monitoring interventions evaluated, fetal movement counting and Doppler monitoring were promising for further evaluation in high-risk pregnancies in low-resource settings. Low amniotic fluid measurements were strongly predictive of stillbirth, but interventions to restore adequate amniotic fluid volume or to deliver the baby based on identification of oligohydramnios have not been systematically tested to conclude whether amniotic fluid assessment is a useful diagnostic tool that leads to actions which prevent stillbirth.

**Table 18:** Collective grading of evidence for impact of monitoring interventions in pregnancy on stillbirth and related perinatal outcomes

	**Evidence of no or negative impact** (leave out of programmes)	**Uncertain evidence** (need for additional research before including in programmes)	**Some evidence** (may include in programmes, but further evaluation is warranted)	**Clear evidence** (merits inclusion in programmes)
Pregnancy risk screening		**X**		

Fetal movement counting		**X**	**X** **(for high-risk pregnancies)**	

Ultrasound scanning		**X**		

Doppler velocimetry		**X (uterine artery and unselected populations)**	**X (umbilical artery and ductus venosus in high-risk pregnancies)**	

Pelvimetry		**X**		

Detection and management of diabetes mellitus		**X**		

Antepartum fetal heart rate monitoring with cardiotocography (NST, CST)		**X**		

Fetal BPP scoring		**X**		

Vibroacoustic stimulation		**X**		

Amniotic fluid volume assessment		**X (oligohydramnios clear risk factor)**		

Home vs. hospital bed rest and monitoring for high-risk pregnancies		**X**		

In-hospital fetal surveillance unit		**X**		

Use of the partograph		**X**		

Intrapartum cardiotocography and pulse oximetry			**X (based largely on observational evidence)**	

### Research gaps

Despite the existence of many tools, devices, and techniques for monitoring pregnancy for complications, there is a dearth of rigorous evidence that any screening and monitoring intervention has a direct impact on stillbirth rates in unselected and low-risk populations. This stems from several common shortcomings in the design and interpretation of studies investigating the use of screening and monitoring techniques. First, the small size and insufficient rigor of many screening and monitoring studies to date renders them underpowered to detect significant differences in perinatal mortality subsequent to their implementation. Many studies assessing the impact of the interventions covered in this review did not report stillbirths or perinatal mortality at all. For virtually all the interventions included in this review (with the exception of x-ray pelvimetry), there is a need for RCTs sufficiently powered to detect statistically significant impact on stillbirth or perinatal mortality, if such differences exist. Second, for screening and monitoring interventions to reduce stillbirth incidence, they must effectively identify women at higher risk of stillbirth in time for an appropriate and effective intervention to be provided. Most studies we reviewed limited their analysis to the first step of successful screening or monitoring: detection of women at increased risk of stillbirth or perinatal death. A number of studies showed that positive test results corresponded fairly well with increased risk of adverse pregnancy outcome. However, a trial of an otherwise effective screening method will show no impact on stillbirths if the subsequent intervention fails to prevent stillbirth, either because intervention is ineffective or provided too late. In most of the studies we reviewed, few women who screened positive were treated according to an established protocol. This flaw precludes assessment of whether the lack of impact of any given screening technique is attributable to a failed screening method or a failed intervention. This fact highlights the need for time-to-decision and time-to-intervention studies, as well as analyses that consider which interventions are employed, and protocols for employing them, subsequent to adverse findings of screening and monitoring studies. The quality, appropriateness, and timeliness of the intervention care provided must also be considered along with the effectiveness of screening or monitoring in detecting true complications.

In addition to these tests of diagnostic accuracy and intervention effectiveness, other types of studies are also needed, including innovative pilot studies, safety studies, and effectiveness trials, to bolster the weak evidence base for screening and monitoring interventions during labour (Table 19). In high-income countries, there is a particular need for better understanding of placental pathophysiology, as up to half of unexplained stillbirths show signs of growth restriction, much of which is attributed to placental insufficiency [158]. Placental dysfunction is often observed in other major known causes of stillbirth including pre-eclampsia and abruption, suggesting the need for accurate placental function or placental biomarker screening tests, especially those appropriate for use in unselected populations. One such test in early pregnancy which measures pregnancy associated plasma protein-A has been associated with a 40–50-fold increase in risk of stillbirth due to placental dysfunction in a selected population, but it is unclear what interventions could prevent stillbirth among women with such positive test results [159]. Appropriate management of pregnancies complicated by placental dysfunction and/or fetal growth restriction requires further research, particularly when detected too early in pregnancy to consider early delivery.

**Table 19:** Research gaps

***Pilot/cohort studies of interventions***
• Alternative imaging or diagnostic technologies (alternatives to X-ray pelvimetry) to predict cephalopelvic disproportion in the antepartum period
• Pathophysiology of impaired placentation and identification of clinical markers of poor placentation/perfusion to develop tests of stillbirth risk
• Development of optimal methodologies for vibroacoustic stimulation (frequency, placement, amplitude, etc), and studies of efficacy and predictive reliability
• Safety of vibroacoustic stimulation (auditory function, cognitive development) and pulse oximetry (cognitive development)
• Interventions to prevent and treat oligohydramnios, particularly in cases of intact membranes
• Non-interventional sensitivity, specificity, and predictive value testing of untested screening techniques in unselected populations (low- and high-risk pregnant women)
• Development of predictive variables for stillbirth at term
• Low-tech strategies, such as the partograph, for identifying high-risk pregnancies in low-resource settings
• New adjunctive techniques to improve the positive predictive value of fetal distress and hypoxia of cardiotocography

***Well-designed RCTs of interventions powered to detect stillbirth rates***

• Community-based pregnancy risk screening schemes
• Formal fetal movement monitoring in high-risk pregnancies
◦ Comparisons of different methods
◦ Impact of timing from monitoring-to-intervention on perinatal mortality
• Optimal combinations of tests to screen for fetal growth restriction
• Optimal management of fetal growth restriction and timing of delivery
• Ultrasound assessment of placental appearance (lesions and calcifications) in high-risk pregnancy
• Ability of uterine artery Doppler ultrasound in combination with other testing for pre-eclampsia prediction and subsequent development of prevention measures for women at highest risk
• Optimising glycaemic control in managing diabetes mellitus in pregnancy
• Assessment of stillbirth risk in instances of gestational diabetes and impaired glucose tolerance (little data compared to pre-existing diabetes mellitus)
• Usefulness of BPP in identifying fetal compromise
• Vibroacoustic stimulation studies in labour
• Impact of in-hospital fetal surveillance units on stillbirth outcomes
• Partograph versus no partograph use

***Effectiveness and cost-effectiveness trials in large populations/at scale***

• Cost-benefit analyses of routine ultrasound for gestational age dating and multiple pregnancy detection in resource-poor settings
• Cost-effectiveness studies of fetal surveillance units in hospitals
• Safety of out-of-hospital bed rest and outpatient fetal surveillance in high-risk pregnancies in resource-poor settings (including economic analyses)

Additional questions surround which screening and monitoring interventions could be most effective in low-/middle-income country settings where 98% or more of the global burden of stillbirths occurs [160]. Screening and monitoring are often technology-dependent, as illustrated by the cardiotocographic machine required for the BPP, CST, and NST; and the use of video ultrasound machines for amniotic fluid assessment. This makes some of these interventions impractical or unaffordable in low-resource or remote settings at the present time. However, certain technologies, such as handheld Doppler ultrasound and video ultrasound scanning machines, are increasingly available in low-resource settings; there is a need for studies to define the most cost-effective ways of using these and other available technologies to identify high-risk pregnancies. Additionally, other techniques, such as fetal movement monitoring and the partograph, require little investment other than training of midwives or mothers, and may be appropriate for use in high-risk pregnancies, if these pregnancies can be identified and if the use of these techniques result in improved outcomes, which has not yet been conclusively demonstrated. Additionally, there is some potentially promising evidence that fetal surveillance typically provided on an inpatient basis, often with bed rest, for high-risk pregnancies can be offered on an outpatient basis with bed rest or reduced activity at home without deleterious impact on pregnancy outcomes; this evidence requires confirmatory effectiveness trials in low-resource settings before programmatic adoption of this approach could be recommended.

Practicality, logistical feasibility, and cost-effectiveness are chief concerns in many low-resource areas. Where ANC services are available and widely utilised, the prospects for identifying and monitoring high-risk pregnancy improve. Along with interventions that improve detection of risk factors and complications, improved management and referral of women with complications are critical to manage obstetric risk in low-/middle-income countries. Unfortunately, in areas where ANC attendance is poor, few of these screening and monitoring techniques are likely to be implementable.

### Implications for programmes and clinical practice

A few monitoring approaches showed promise for use in high-risk pregnancies, including fetal movement monitoring and umbilical Doppler velocimetry. Before recommending these interventions for widespread use, further large RCTs of sufficient rigour to detect differences in stillbirth and perinatal outcomes are needed to determine the utility and effectiveness of these monitoring approaches and interventions used after positive tests.

This review identified several interventions – for example, amniotic fluid measurement for signs of oligohydramnios, especially as pregnancy approaches or exceeds term; routine ultrasound scanning, and intrapartum cardiotocography – that are in widespread use in high-resource settings, but for which rigorous evidence is lacking. For these interventions, their continued use is reasonable even though the evidence base for impact on stillbirths is lacking. Intrapartum cardiotocography in particular has been shown in RCTs to elevate the risk of operative delivery. Prevailing wisdom credits cardiotocography and available Caesarean section with the diminution of stillbirth rates in high-income countries in recent decades; however, the role of cardiotocography in reducing stillbirth should be confirmed with more rigorous evidence. Efforts to achieve glycaemic control in cases of maternal diabetes mellitus are also encouraged, despite the lack of strong evidence showing an impact of such interventions on stillbirth, on the grounds that women with good glycaemic control experience fewer complications and negative outcomes of pregnancy.

Particularly in pregnancies deemed high-risk based on screening test results, clinical progression of pregnancy, or reproductive history, multiple antepartum testing modalities can minimise deficiencies in sensitivity and predictive value when used in combination to assess fetal well-being and abnormalities. The BPP reflects one standardised strategy by which tests of fetal well-being have been packaged to permit a more comprehensive assessment of fetal well-being. In cases where one test raises the possibility that a pregnancy is high-risk, multiple tests of fetal well-being should be considered if time allows, along with the patient's overall clinical history, to determine the appropriateness of intervention. Multiple tests are recommended in many protocols for identifying and making clinical decisions about management of pregnancies with suspected fetal growth restriction [161]. In these cases the collective results of a battery of tests including all the components of the BPP and Doppler velocimetry, as well as ultrasound biometry, can be informative in decision-making to maximise gestational age and inform the strategic timing of corticosteroid administration and early delivery via induction or Caesarean section. However, conflicting test results – a recent study showed only 44.5% concordance between BPP and Doppler assessments of compromise in growth-restricted fetuses – can complicate interpretation [162].

Recommendations for clinical decision-making protocols and introduction of interventions in low-/middle-income countries are more difficult. Resource constraints preclude the implementation of many of the interventions reviewed in this paper. The interventions requiring the lowest investment in resources and training – fetal movement monitoring and the partograph – show some evidence of benefit in high-risk pregnancies, but more studies are needed. Where safe Caesarean section is available, ensuring that intermittent or continuous intrapartum fetal heart rate monitoring is performed may improve targeting of Caesarean section while improving birth outcomes.

Screening and monitoring interventions cannot be effective without responsive, quality care and careful clinical management, particularly for high-risk pregnancies. Health systems quality improvement activities, including boosting provider skill in interpreting test results and determining appropriate intervention, timely response including swift referral, and evaluative strategies to document outcomes after intervention are essential complementary activities to ensure that an effective screening or monitoring technique translates to measurable improvements in perinatal mortality outcomes.

### Conclusion

Screening pregnancies to identify risk factors, complications, or indications of fetal distress and providing appropriate surveillance for identified risk factors theoretically encourages appropriate use of interventions, including induction of labour, Caesarean section, and pharmacological treatment, to prevent fetal loss and adverse maternal or neonatal outcomes. Screening and monitoring are futile, however, in the absence of effective interventions to act promptly and appropriately to manage identified risk factors and complications. The weak evidence in this review for impact of screening and monitoring interventions on stillbirth incidence is due at least in part to limitations in study designs that result in uninterpretable data. Screening tests may be ineffective, inappropriate, or occur too late to prevent adverse outcomes, but more problematically, most studies did not report the impact of intervention (particularly standardised intervention) following positive screening test results. Effective interventions exist to prevent stillbirth associated with many maternal infections or conditions (e.g., syphilis, malaria, pre-eclampsia) and placental dysfunction (e.g., oligohydramnios, placental insufficiency, abruption). Because screening and monitoring techniques during pregnancy also pose the risk of inappropriate or unnecessary use of drugs, induction of labour, iatrogenic preterm birth, or Caesarean section, it is practically, ethically, and economically important to validate the sensitivity, specificity, and positive predictive value of available screening and monitoring techniques to protect women and their babies from iatrogenic harm; and to ensure the wise use of scarce medical resources in low- and middle-income settings.

## List of abbreviations used

AFI: amniotic fluid index; ANC: antenatal care; BPP: biophysical profile; CI: confidence interval; CST: contraction stress test; CTG: cardiotocography; ECG: electrocardiogram; FHR: fetal heart rate; HR: hazard ratio; IU: international units; LOE: level of evidence; NS: non-significant; NST: non-stress test; OR: odds ratio; PMR: perinatal mortality rate; PPV: positive predictive value; PROM: premature rupture of membranes; PPROM: pre-term premature rupture of membranes; RCT: randomised clinical/controlled trial; RD: risk difference; RI: resistance index; RR: relative risk/risk ratio; SB: stillbirth; SBR: stillbirth rate; SD: standard deviation; SGA: small for gestational age; TBA: traditional birth attendant; UK: United Kingdom; USA: United States of America; WHO: World Health Organisation; WMD: weighted mean difference.

## Competing interests

The authors declare that they have no competing interests.

## Authors' contributions

The paper was written and reviewed by all the authors.

## Supplementary Material

Additional file 1 **Web Table 1. Component studies in Mangesi et al. 2007 meta-analysis: Impact of fetal movement monitoring**. Component studies in Mangesi et al. 2007 meta-analysis showing impact on stillbirths/perinatal mortality Click here for file

Additional file 2 **Web Table 2. Component studies in Neilson 1998 meta-analysis: Impact of routine ultrasound in pregnancy**. Component studies in Neilson 1998 meta-analysis showing impact on stillbirths/perinatal mortality Click here for file

Additional file 3 **Web Table 3. Component studies in Bricker et al. 2008 meta-analysis: Impact of ultrasound in late pregnancy**. Component studies in Bricker et al. 2008 meta-analysis showing impact on stillbirths/perinatal mortality Click here for file

Additional file 4 **Web Table 4. Component studies in Papageorghiou et al. 2002: Impact of second trimester uterine artery Doppler on perinatal mortality**. Component studies in Papageorghiou et al. 2002 meta-analysis showing impact on stillbirths/perinatal mortality Click here for file

Additional file 5 **Web Table 5. Component studies in Baschat 2004: impact of Doppler velocimetry on stillbirth and perinatal mortality in pre-term growth-restricted fetuses**. Component studies in Baschat 2004 meta-analysis showing impact on stillbirths/perinatal mortality Click here for file

Additional file 6 **Web Table 6. Component studies in Neilson et al. 2000 meta-analysis: Impact of Doppler screening during pregnancy**. Component studies in Neilson et al. 2000 meta-analysis showing impact on stillbirths/perinatal mortality Click here for file

Additional file 7 **Web Table 7. Component studies in Pattinson et al. 1997 meta-analysis: Impact of pelvimetry during pregnancy**. Component studies in Pattinson et al. 1997 meta-analysis showing impact on stillbirths/perinatal mortality Click here for file

Additional file 8 **Web Table 8. Component studies in Russell et al. 2007 meta-analysis: Impact of management of gestational diabetes on stillbirth and perinatal outcomes**. Component studies in Russell et al. 2007 showing impact on stillbirths/perinatal mortality Click here for file

Additional file 9 **Web Table 9. Component studies in Mukhopadhyay et al. 2007: Impact of continuous subcutaneous insulin infusion on intrauterine fetal death**. Component studies in Mukhopadhyay et al. 2007 showing impact on stillbirths/perinatal mortality Click here for file

Additional file 10 **Web Table 10. Component studies in Boulvain et al. 2001 meta-analysis: Impact of elective delivery on perinatal mortality**. Component studies in Boulvain et al. 2001 showing impact on stillbirths/perinatal mortality Click here for file

Additional file 11 **Web Table 11. Component studies in Pattison and McCowan 1999 meta-analysis: Impact of cardiotocography for antepartum fetal assessment on perinatal mortality**. Component studies in Pattison and McCowan 1999 showing impact on stillbirths/perinatal mortality Click here for file

Additional file 12 **Web Table 12. Component studies in Lalor et al. 2008 meta-analysis: Impact of fetal biophysical profile**. Component studies in Lalor et al. 2008 showing impact on stillbirths/perinatal mortality Click here for file

Additional file 13 **Web Table 13. Component studies in Tan et al. 2001 meta-analysis: Impact of vibroacoustic stimulation**. Component studies in Tan et al. 2001 meta-analysis showing impact on stillbirths/perinatal mortality Click here for file

Additional file 14 **Web Table 14. Component studies in Magann et al 2007 review: impact of idiopathic polyhydramnios on stillbirth and perinatal mortality**. Component studies in Magann et al. 2007 review showing impact on stillbirths/perinatal mortality Click here for file

Additional file 15 **Web Table 15. Component studies in Nabhan and Abdelmoula 2008 **[185]**meta-analysis: comparison of single deepest vertical pocket vs. AFI in predicting perinatal outcome**. Component studies in Nabhan and Abdelmoula 2008 review showing impact on stillbirths/perinatal mortality Click here for file

Additional file 16 **Web Table 16. Component studies in Hofmeyr and Gulmezoglu 2002 **[126]**meta-analysis: impact of maternal hydration on amniotic fluid volume and perinatal outcomes**. Component studies in Hofmeyr and Gulmezoglu 2002 review showing impact on stillbirths/perinatal mortality Click here for file

Additional file 17 **Web Table 17. Component studies in Crowther 2001 meta-analysis: Impact of hospitalisation for bed rest for women with a multiple pregnancy on stillbirth and perinatal mortality**. Component studies in Crowther 2001 review showing impact on stillbirths/perinatal mortality Click here for file

Additional file 18 **Web Table 18. Component studies in Meher et al. 2005: Impact of some rest in hospital vs. routine activity at home on stillbirth and perinatal mortality**. Component studies in Meher et al. 2005 review showing impact on stillbirths/perinatal mortality Click here for file

Additional file 19 **Component studies in Kroner et al. 2001: Impact of antenatal day care units on perinatal mortality**. Component studies in Kroner et al. 2001 review showing impact on stillbirths/perinatal mortality Click here for file

Additional file 20 **Web Table 20. Component studies in Lavender et al. 2008 meta-analysis: Impact of different designs of partogram on neonatal morbidity or perinatal mortality**. Component studies in Lavender et al. 2008 review showing impact on stillbirths/perinatal mortality Click here for file

Additional file 21 **Web Table 21. Component studies in Alfirevic and Devane 2006 meta-analysis: Impact of continuous cardiotocography on stillbirth and perinatal mortality**. Component studies in Lavender et al. 2008 review showing impact on stillbirths/perinatal mortality Click here for file

Additional file 22 **Web Table 22. Component studies in Neilson 2006 meta-analysis: Impact of fetal electrocardiogram (ECG) on perinatal mortality**. Component studies in Neilson meta-analysis showing impact on stillbirths/perinatal mortality Click here for file

Additional file 23 **Web Table 23. Component studies in East et al. 2007 meta-analysis: Impact of fetal pulse oximetry on fetal/neonatal death**. Component studies in East et al. 2007 meta-analysis showing impact on stillbirths/perinatal mortality Click here for file
