# Supplementary material for: Reducing stillbirths: screening and monitoring during pregnancy and labour
Source: BMC Pregnancy Childbirth. 2009 May 7;9(Suppl 1):S5. doi: 10.1186/1471-2393-9-S1-S5 (PMC2679411; doi:10.1186/1471-2393-9-S1-S5)
Supplement: Additional file 1 — Web Table 1. Component studies in Mangesi et al. 2007 meta-analysis: Impact of fetal movement monitoring. Component studies in Mangesi et al. 2007 meta-analysis showing impact on stillbirths/perinatal mortality [file 1471-2393-9-S1-S5-S1.doc]

**Web Table 1. Component studies in Mangesi et al. 2007 [1] meta-analysis: Impact of fetal movement monitoring**

| **Source** | **Location and Type of Study** | **Intervention** | **Stillbirths / Perinatal outcomes** |
| --- | --- | --- | --- |
| 1. Gomez et al. 2003 [2] | Peru. Hospital-based study.  RCT. High-risk, singleton pregnant women (N=1400). (N=700 intervention group, N=700 controls). | To compare the impact of Latin American Centre for Perinatology and human development fetal movement chart method (intervention) versus ‘count-to-ten’ Cardiff method (controls). | Fetal death (miscarriage + SB): RR not estimable.  [0/700 vs. 0/700 in intervention and control groups, respectively]. |
| 2. Grant et al. 1989 [3] | UK (Oxford). 66 clusters.  Cluster RCT. Pregnant women 28-32 wks gestation. (N=68,654; N=31993 intervention, N=36661 controls). | To compare the impact of counting fetal movements formally every day using a ‘count-to-ten’ chart (Cardiff) (intervention) vs. the control group where the fetal movements were not monitored, but women were asked about fetal movements at each antenatal visit and allowed to raise concerns. | SBR: weighted mean difference=0.23 (95% CI: -0.61-1.07)**[NS]**  [mean (SD)=2.90 (1.90) vs. 2.67 (1.55) in intervention vs. controls, respectively.] |
| 3. Thomsen et al. 1990 [4] | Denmark.  RCT. Pregnant women 16-18 wks gestation without obstetric complications and medical diseases (N=1,191; N=577 intervention, N=614 controls). | To compare the impact of fetal movement counting by modified Cardiff method (intervention) vs. hormonal analysis (controls). | SBR: RR=3.19 (95% CI: 0.13-78.20)**[NS]**  [1/577 vs. 0/614 in intervention and control groups, respectively]. |

References

1. Mangesi L, Hofmeyr GJ: **Fetal movement counting for assessment of fetal wellbeing**. *Cochrane Database of Systematic Reviews* 2007, **1**:CD004909.

2. Gomez L, Padilla L, De La Vega G, Bautista F, Villar A: **Compliance with a fetal movement chart by high risk patients**. *American Journal of Obstetrics and Gynecology* 2003, **189**:S179.

3. Grant A, Elbourne D, Valentin L, Alexander S: **Routine formal fetal movement counting and risks of antepartum late deaths in nomally formed singletons**. *Lancet* 1989, **2**:345-347.

4. Thomsen SG, Legarth J, Weber T, Kristensen J: **Monitoring of normal pregnancies by daily fetal movement registration or hormone assessment. A random allocation study**. *Journal of Obstetrics and Gynaecology* 1990, **10**:189-193.
