# Supplementary material for: Reducing stillbirths: screening and monitoring during pregnancy and labour
Source: BMC Pregnancy Childbirth. 2009 May 7;9(Suppl 1):S5. doi: 10.1186/1471-2393-9-S1-S5 (PMC2679411; doi:10.1186/1471-2393-9-S1-S5)
Supplement: Additional file 2 — Web Table 2. Component studies in Neilson 1998 meta-analysis: Impact of routine ultrasound in pregnancy. Component studies in Neilson 1998 meta-analysis showing impact on stillbirths/perinatal mortality [file 1471-2393-9-S1-S5-S2.doc]

**Web Table 2. Component studies in Neilson 1998 meta-analysis [1]: Impact of routine ultrasound in pregnancy**

| **Source** | **Location and Type of Study** | **Intervention** | **Stillbirths / Perinatal Outcomes** |
| --- | --- | --- | --- |
| 1. Eik-Nes et al. [2-4] | Norway (Alesund).  RCT. Pregnant women (N=1628); recruitment 1979-1981. | Compared the impact on perinatal mortality of routine ultrasound examinations at 18 (biparietal diameter measured) and 32 wks (biparietal diameter and abdominal circumference) with additional examination at 36 wks if SGA or breech (intervention) vs. selective examination for specific clinical indications only (controls; only 23% of controls had an ultrasound). | PMR: OR=0.58 (95% CI: 0.22-1.56) **[NS]**  [6/794 vs. 10/765 in intervention and control groups, respectively]. |
| 2. Saari-Kemppainen [5-7] | Finland (Helsinki). Tertiary care setting.  RCT. N=9310 women (N=4691 intervention, N=4619 controls). Recruitment 1986-1987. | Compared the impact of routine ultrasound examination at 16-20 wks (intervention) vs. selective scanning for specific reasons (controls). | PMR: OR=0.52 (95% CI: 0.31-0.86)  [20/4389 vs. 39/4347 in intervention vs. control groups, respectively]. |
| 3. Bennett et al. 1982 [8] | UK (London). Consultant antenatal clinics.  RCT. N=1062 women. | Compared the impact on perinatal mortality of the ultrasound results revealed (intervention) vs. concealed (controls). All women underwent ultrasonic examination at ~16 wks. | PMR: OR=1.65 (95% CI: 0.41-6.64) **[NS]**  [5/531 vs. 3/531 in intervention vs. control groups, respectively]. |
| 4. Ewigman et al. 1990 [9] | USA (Missouri). Tertiary care setting.  RCT. N=915 women. | To assess the impact on perinatal mortality of routine ultrasound - optimally at 10-12 wks, but permissible up to 18 wks (intervention). Ultrasound for specific indications permitted at any time in either group. | PMR: OR=0.53 (95% CI: 0.11-2.65) **[NS]**  [2/404 vs. 4/420 in intervention vs. control groups, respectively]. |
| 5. LeFevre and the RADIUS study group 1993 [10-12] | USA. Tertiary care setting.  RCT. N=15,530 women. | Compared the impact on perinatal mortality of intended ultrasound screen at 18-20 and at 31-33 wks gestation (intervention) vs. selective ultrasonography for specific reasons only (controls). | PMR: OR=1.25 (95% CI: 0.83-1.89) **[NS]**  [52/7685 vs. 41/7596 in intervention vs. control groups, respectively]. |
| 6. Kieler et al. [13-15] | Sweden (Stockholm). Tertiary care setting.  RCT. N=4997 (N=2482 intervention group, N=2515 controls). | To assess the impact of an ultrasound scan at about 15 wks (range 13-19 wks; 98.7% had scan)(intervention) vs. no scan (controls; 4.1% of controls had scan < 19 wks, 31% had scan > 19 wks). | PMR: OR=1.01 (95% CI: 0.45-2.25) **[NS]**  [12/2413 vs. 12/2432 in intervention vs. control groups, respectively]. |
| 7. Bakketeig et al. 1984 [16] | Norway (Trondheim).  RCT. N=1009 pregnant women attending for ANC 1979-1980. | To assess the effect of ultrasound examinations at both 19 and 32 wks gestation + routine ANC (intervention) vs. routine ANC only (controls). | PMR: OR=0.98 (95% CI: 0.28-3.41) **[NS]**  [5/516 vs. 5/507 in intervention vs. control groups, respectively]. |
| 8. Geerts et al. 1996 [17] | South Africa (Tygerberg). Tertiary care setting.  RCT. Women <24 wks of pregnancy. | Compared the effect of 'level 1' ultrasound examination by obstetric registrar or medical officer (intervention) vs. ultrasound examination only for specific clinical indication (controls; 25% had ultrasound examination at some time). | PMR: OR=0.68 (95% CI: 0.29-1.59) **[NS]**  [9/460 vs. 13/455 in intervention vs. control groups, respectively]. |

References

1. Neilson J: **Ultrasound for fetal assessment in early pregnancy**. *Cochrane Database of Systematic Reviews;* 1998(4):CD000182.

2. Eik-Nes SH, Salvesen KA, Vatten LJ, Okland O: **Effects of routine two-stage ultrasound screening in pregnancy: the Alesund randomised controlled trial revisited** In*.*; [Submitted for publication].

3. Eik-Nes SH, Okland O, Aure JC, Ulstein M: **Ultrasound screening in pregnancy: a randomised controlled trial**. *Lancet* 1984, **1**(8390):1347.

4. Salvesen K: **Routine ultrasonography in utero and development in childhood - a randomized controlled follow-up study**. University of Trondheim; 1993.

5. Saari-Kemppainen A: **Use of antenatal care services in a controlled ultrasound screening trial**. *Acta Obstet Gynecol Scand* 1995, **74**(1):12-14.

6. Saari-Kemppainen A, Karjalainen O, Ylostalo P, Heinonen OP: **Fetal anomalies in a controlled one-stage ultrasound screening trial. A report from the Helsinki Ultrasound Trial**. *J Perinat Med* 1994, **22**(4):279-289.

7. Saari-Kemppainen A, Karjalainen O, Ylostalo P, Heinonen OP: **Ultrasound screening and perinatal mortality: controlled trial of systematic one-stage screening in pregnancy. The Helsinki Ultrasound Trial**. *Lancet* 1990, **336**(8712):387-391.

8. Bennett MJ, Little G, Dewhurst J, Chamberlain G: **Predictive value of ultrasound measurement in early pregnancy: a randomized controlled trial**. *Br J Obstet Gynaecol* 1982, **89**(5):338-341.

9. Ewigman B, LeFevre M, Hesser J: **A randomized trial of routine prenatal ultrasound**. *Obstet Gynecol* 1990, **76**(2):189-194.

10. LeFevre ML, Bain RP, Ewigman BG, Frigoletto FD, Crane JP, McNellis D: **A randomized trial of prenatal ultrasonographic screening: impact on maternal management and outcome. RADIUS (Routine Antenatal Diagnostic Imaging with Ultrasound) Study Group**. *Am J Obstet Gynecol* 1993, **169**(3):483-489.

11. LeFevre ML, Evans JK, Ewigman B: **Is smoking an indication for prenatal ultrasonography? RADIUS Study Group**. *Arch Fam Med* 1995, **4**(2):120-123.

12. Ewigman BG, Crane JP, Frigoletto FD, LeFevre ML, Bain RP, McNellis D: **Effect of prenatal ultrasound screening on perinatal outcome. RADIUS Study Group**. *N Engl J Med* 1993, **329**(12):821-827.

13. Kieler H, Axelsson O, Haglund B, Nilsson S, Salvesen KA: **Routine ultrasound screening in pregnancy and the children's subsequent handedness**. *Early Hum Dev* 1998, **50**(2):233-245.

14. Kieler H, Haglund B, Waldenstrom U, Axelsson O: **Routine ultrasound screening in pregnancy and the children's subsequent growth, vision and hearing**. *Br J Obstet Gynaecol* 1997, **104**(11):1267-1272.

15. Waldenstrom U, Axelsson O, Nilsson S, Eklund G, Fall O, Lindeberg S, Sjodin Y: **Effects of routine one-stage ultrasound screening in pregnancy: a randomised controlled trial**. *Lancet* 1988, **2**(8611):585-588.

16. Bakketeig LS, Eik-Nes SH, Jacobsen G, Ulstein MK, Brodtkorb CJ, Balstad P, Eriksen BC, Jorgensen NP: **Randomised controlled trial of ultrasonographic screening in pregnancy**. *Lancet* 1984, **2**(8396):207-211.

17. Geerts LT, Brand EJ, Theron GB: **Routine obstetric ultrasound examinations in South Africa: cost and effect on perinatal outcome--a prospective randomised controlled trial**. *Br J Obstet Gynaecol* 1996, **103**(6):501-507.
