# Supplementary material for: Reducing stillbirths: screening and monitoring during pregnancy and labour
Source: BMC Pregnancy Childbirth. 2009 May 7;9(Suppl 1):S5. doi: 10.1186/1471-2393-9-S1-S5 (PMC2679411; doi:10.1186/1471-2393-9-S1-S5)
Supplement: Additional file 3 — Web Table 3. Component studies in Bricker et al. 2008 meta-analysis: Impact of ultrasound in late pregnancy. Component studies in Bricker et al. 2008 meta-analysis showing impact on stillbirths/perinatal mortality [file 1471-2393-9-S1-S5-S3.doc]

**Web Table 3. Component studies in Bricker et al. 2008 meta-analysis [1]: Impact of ultrasound in late pregnancy**

| **Source** | **Location and Type of Study** | **Intervention** | **Stillbirths / Perinatal Outcomes** |
| --- | --- | --- | --- |
| 1. Eik-Nes 2000 [2] | Norway.  RCT. High-risk pregnant women (N = 1628). | To compare the impact of routine ultrasound examination at 18 weeks (biparietal diameter measured) and 32 weeks (biparietal diameter and mean abdominal diameter) with additional examination at 36 weeks' gestation if fetus small-for-gestational age and/or presenting by breech (intervention) vs. selective examination for clinical indications only (controls). | PMR: RR = 0.58 (95% CI: 0.21 – 1.58] **[NS]**.  [6/794 vs. 10/765 in intervention and control groups, respectively]. |
| 2. McKenna et al. 2003 [3] | UK.  RCT. Low risk, singleton women (N = 1998) recruited over a 21 month period. | To assess the impact of ultrasound examinations by the specially trained midwife to assess liquor volume, fetal weight and placental maturity (intervention). The intervention group also had assessment at 30-32 weeks and at 36-37 weeks by a midwife as part of routine care with midwife estimate of fetal size, presentation, position and amniotic fluid volume. The comparison group had selective ultrasound examinations if indicated. | SBR: RR = 2.00 (95% CI: 0.18 – 22.02) [NS].  [2/999 vs. 1/999 in intervention and control groups, respectively].  PMR: same as above. |
| 3. Neilson et al. 1984 [4] | UK.  Pseudorandomization. Low risk pregnant women (N = 877). | To compare the impact of ultrasound results revealed (intervention) vs. concealed (controls). In the study group, the ultrasound results were plotted and reported in the case notes (i.e. revealed). No requests for control group measurements to be revealed occurred, but this option was available to clinicians. All women (both intervention and control) had an ultrasound examination < 24 weeks' gestation for gestational dating. All had further ultrasound scan at 34-36.5 weeks' gestation to measure crown rump length and trunk area. | SBR: RR = not estimable.  [0/433 vs. 0/444 in intervention and control groups, respectively].  PMR: RR = 0.34 (95% CI: 0.01 – 8.37) **[NS]**.  [0/433 vs. 1/444 in intervention and control groups, respectively]. |
| 4. Duff 1993 [5] | New Zealand.  RCT. Pregnant women (N = 1527). | To compare the impact of ultrasound in late gestation (intervention) vs. ultrasound if clinically indicated (controls). All women had a dating scan 16-24 weeks' gestation. Study group had a further scan at 32-36 weeks' gestation (ideally 34 weeks' gestation) which aimed to detect small-for-gestational-age fetuses, and if estimated fetal weight fell below the 20th centile for gestation, this was reported and additional scans recommended but not arranged. Clinicians were able to order further scans for the control group if clinically indicated. | SBR: RR = 4.01 (95% CI: 0.85 – 18.80) **[NS]**.  [8/763 vs. 2/764 in intervention and control groups, respectively].  PMR: RR = 2.50 (95% CI: 0.79 – 7.95) **[NS]**.  [10/763 vs. 4/764 in treatment and control groups, respectively]. |
| 5. Proud and Grant 1987. [6] | Peterborough.  RCT. Pregnant women (N = 2000) attending the ultrasound department for routine third trimester scans, including multiple pregnancies. | To assess the impact on PMR if the placental grading at the routine third trimester scan was revealed (intervention) vs. concealed (controls).  All women were offered routine early pregnancy ultrasound and 2 routine scans in the third trimester. Clinical management in both groups was left entirely to the clinician responsible for care. | SBR: RR = 0.08 (95% CI: 0.01 – 0.64).  [1/1014 vs. 12/1011 in intervention and control groups, respectively].  PMR: RR = 0.31 (95% CI: 0.10 – 0.94).  [4/1014 vs. 13/1011 in treatment and control groups, respectively]. |
| 6. Ewigman et al. 1993 [7] | USA.  RCT. Pregnant women (N = 15151). | To compare the ultrasound screening at 18-20 weeks' and 31-33 weeks' gestation (intervention) vs. selective ultrasonography (controls). | SBR: RR = 1.46 (95% CI: 0.86 – 2.48) **[NS]**.  [34/7685 vs. 23/7596 in intervention and control groups, respectively].  PMR: RR = 1.25 (95% CI: 0.83 – 1.89) **[NS]**.  [52/7685 vs. 41/7596 in intervention and control groups, respectively]. |
| 7. Bakketeig et al. 1984 [8] | Norway (Trondheim).  RCT. Pregnant women (N = 1009) attending for antenatal care between 1979-1980. | Compared the impact of ultrasound examinations at 19 weeks and 32 weeks' gestation + routine antenatal care (intervention) vs. routine care only (controls). | PMR: RR = 0.98 (95% CI: 0.29 – 3.36) **[NS]**.  [5/510 vs. 5/499 in intervention and control groups, respectively]. |
| 8. Newnham et al. 1993 [9] | Australia.  RCT. Singleton pregnancies (N = 2834). | Compared the impact on perinatal mortality of the ‘intensive group’ (intervention) vs. the ‘regular’ group (controls). The 'regular' group had an ultrasound examination at 18 weeks for fetal biometry, subjective amniotic fluid assessment and placental morphology and location, and any further scans in pregnancy were conducted on clinician’s request. The 'intensive group' had the aforementioned ultrasound examination, plus an amniotic fluid index and continuous wave Doppler ultrasound of the umbilical artery and an arcuate artery within the placental vascular bed at 18, 24, 28, 34 and 38 weeks' gestation. The Doppler ultrasound parameter reported was systolic/diastolic ratio. Results of these examinations were recorded in the hospital chart, but no clinical management guidance was given. | SBR: RR = 0.84 (95% CI: 0.36 – 1.93) **[NS]**.  [10/1415 vs. 12/1419 in intervention and control groups, respectively].  PMR: RR = 0.59 (95% CI: 0.30 – 1.17) **[NS]**.  [13/1415 vs. 22/1419 in intervention and control groups, respectively]. |

References

1. Bricker L, Neilson JP, Dowswell T: **Routine ultrasound in late pregnancy (after 24 weeks' gestation)**. *Cochrane Database Syst Rev* 2008(4):CD001451.

2. Eik-Nes SH, Salvesen KA, Okland O, Vatten LJ: **Routine ultrasound fetal examination in pregnancy: the 'Alesund' randomized controlled trial**. *Ultrasound Obstet Gynecol* 2000, **15**(6):473-478.

3. McKenna D, Tharmaratnam S, Mahsud S, Bailie C, Harper A, Dornan J: **A randomized trial using ultrasound to identify the high-risk fetus in a low-risk population**. *Obstet Gynecol* 2003, **101**(4):626-632.

4. Neilson JP, Munjanja SP, Whitfield CR: **Screening for small for dates fetuses: a controlled trial**. *Br Med J (Clin Res Ed)* 1984, **289**(6453):1179-1182.

5. Duff GB: **A randomized controlled trial in a hospital population of ultrasound measurement screening for the small for dates baby**. *Aust N Z J Obstet Gynaecol* 1993, **33**(4):374-378.

6. Proud J, Grant AM: **Third trimester placental grading by ultrasonography as a test of fetal wellbeing**. *Br Med J (Clin Res Ed)* 1987, **294**(6588):1641-1644.

7. Ewigman BG, Crane JP, Frigoletto FD, LeFevre ML, Bain RP, McNellis D: **Effect of prenatal ultrasound screening on perinatal outcome. RADIUS Study Group**. *N Engl J Med* 1993, **329**(12):821-827.

8. Bakketeig LS, Eik-Nes SH, Jacobsen G, Ulstein MK, Brodtkorb CJ, Balstad P, Eriksen BC, Jorgensen NP: **Randomised controlled trial of ultrasonographic screening in pregnancy**. *Lancet* 1984, **2**(8396):207-211.

9. Newnham JP, Evans SF, Michael CA, Stanley FJ, Landau LI: **Effects of frequent ultrasound during pregnancy: a randomised controlled trial**. *Lancet* 1993, **342**(8876):887-891.
