# Supplementary material for: Reducing stillbirths: screening and monitoring during pregnancy and labour
Source: BMC Pregnancy Childbirth. 2009 May 7;9(Suppl 1):S5. doi: 10.1186/1471-2393-9-S1-S5 (PMC2679411; doi:10.1186/1471-2393-9-S1-S5)
Supplement: Additional file 4 — Web Table 4. Component studies in Papageorghiou et al. 2002: Impact of second trimester uterine artery Doppler on perinatal mortality. Component studies in Papageorghiou et al. 2002 meta-analysis showing impact on stillbirths/perinatal mortality [file 1471-2393-9-S1-S5-S4.doc]

**Web Table 4. Component studies in Papageorghiou et al. 2002 [1]: Impact of second trimester uterine artery Doppler on perinatal mortality**

| **Source** | **Location and Type of Study** | **Intervention** | **Stillbirths / Perinatal Outcomes** |
| --- | --- | --- | --- |
| Albaiges et al. (2000) [2] | UK.  Screening study. N = 1757 singleton pregnancies. | To evaluate the role of colour Doppler of uterine arteries at 23 weeks’ gestation in predicting adverse pregnancy outcome. | The sensitivity of bilateral notches or mean pulsatility index above 1.45 was 83.3% for fetal death, with positive predictive value being 3.9%. |
| Aquilina et al. (2001) [3] | UK.  Screening efficacy study. N = 689 women with inhibin-A levels measured before 20 weeks of gestation. | To assess the efficacy of inhibin-A alone, uterine artery Doppler alone, and inhibin-A combined with Doppler at 20 weeks’ gestation in predicting pre-eclampsia according to the different cut-off values of inhibin-A and resistance indices. | The combined Doppler and inhibin-A was better than Doppler (P < 0.05) or inhibin-A (P < 0.001) alone at false-positive rate of around 7% in terms of sensitivity. The positive likelihood ratio was greater for the combined method, compared to Doppler or inhibin-A alone. |
| Bewley et al. (1991) [4] | UK.  Screening, cross-sectional study. N = 977 women at 16-24 weeks of gestation. | To evaluate the screening characteristics of a mid-trimester uteroplacental Doppler scan. | Pregnancies with high averaged resistance index had a greater prevalence of proteinuric hypetension, placental abruption, small-for-gestational age infants, and fetal loss. |
| Bower et al. (1993) [5] | UK.  Cross-sectional study. N = 2430 women. | To assess the value of continuous wave uterine arteries Doppler ultrasound as a screening test in unselected population. | The predictive values of abnormal flow velocity waveforms for moderate and severe pre-eclampsia were better than for other complications of pregnancy (sensitivity 79-88%, specificity 85%). |
| Bower et al. (1993) [6] | UK.  Screening study. N = 2058 unselected women. | To determine the value of the early diastolic notch and colour Doppler imaging of the uterine arteries as a screening test for pre-eclampsia. | Significant pre-eclampsia: 68-fold higher in women with a persistent notch at 24 weeks. |
| Frusca et al. (1997) [7] | Italy.  Screening study. N = 456 nulliparous women. | To evaluate the role of uterine Doppler velocimetry at 20 and 24 weeks gestation in predicting gestational hypertension and small-for-gestational age babies. | Pregnancy complications more common in women with high uteroplacental resistance index (10%) vs. those with normal uterine artery velocimetry (P = 0.0001). |
| Harrington et al. (1996) [8] | Germany. University hospital ultrasound department.  Prospective, cross-sectional analysis. N = 1326 unselected women. | To evaluate the use of Doppler of the uterine arteries at 19-21 weeks of gestation in predicting pre-eclampsia and intrauterine growth retardation. | Women with notching had a significantly higher risk of developing proteinuric pregnancy-induced hypertension (p < 0.00001) or delivering a small-for-gestational age baby (p < 0.00001). |
| Irion et al. (1998) [8] | Switzerland. Tertiary care university hospital.  Prospective cohort study. N = 1311 nulliparous women. | Evaluation of uterine artery blood flow velocity waveforms analysis to predict pre-eclampsia and low birth weight. | All abnormalities of the studied Doppler indices were significantly associated with pre-eclampsia and low birth weight. |
| Kurdi et al. (1998) [9] | Saudi Arabia.  Screening study. N = 1022 unselected women at the time of the 20-week anomaly scan. | To determine the role of one-stop Doppler imaging of the uterine arteries at the time of the 20-week anomaly scan to determine women at risk of pre-eclampsia and intrauterine growth restriction (IUGR). | Pre-eclampsia: OR = 12.8 (95% CI: 5.3 – 30.8) for women with bilateral notches; OR = 0.11 (95% CI: 0.04 – 0.28) in women with normal uterine artery Doppler studies. |
| Newnham et al. (1990) [10] | Australia.  Prospective double-blind study. N = 535 medium-risk pregnancies. | To assess the role of Doppler flow velocity waveform systolic to diastolic (S/D) ratios in predicting fetal hypoxia and IUGR during the second and third trimester of pregnancy. | There were three pregnancy losses, and the preceding S/D ratios in these were within the normal range.  Umbilical artery S/D ratios significantly associated with IUGR. Uteroplacental S/D ratios less predictive of IUGR.  Umbilical artery S/D ratios not significantly associated with fetal hypoxia at 28 weeks, but at 34 weeks. Uteroplacental S/D ratios not predictive of fetal hypoxia. |
| North et al. (1994) [11] | Australia and New Zealand.  Screening study. N = 458 women. | Compared the clinical outcomes in women with abnormal versus normal placental uterine artery resistance on Doppler ultrasound. | PMR: 1/77 (1.3%) vs. 1/380 (0.03%) **[NS]** in abnormal vs. normal groups. |
| Papageorghiou et al. (2001) [12] | UK. (London). Multicentered (seven hospitals).  Screening study. N = 8335 consecutive singleton pregnancies at 23 weeks of gestation. | The role of transvaginal uterine artery colour Doppler in predicting subsequent development of pre-eclampsia and fetal growth restriction (FGR). | Pre-eclampsia with FGR was better predicted (69% sensitivity) by increased pulsatility index (PI) than pre-eclampsia (23.9%) or FGR (13.2%) alone. The sensitivity of bilateral notches was similar to that of increased PI in predicting these complications. The best values were when abnormal result was either high PI or presence of notches, but the screen-positive rate was increased. |
| Steel et al. (1990) [13] | UK.  Screening study. N = 1198 nulliparous women at a median of 18 weeks’ gestation. | To assess the association between uteroplacental Doppler ultrasound waveforms with pregnancy complications. | Hypertension: more frequent among women with abnormal waveforms vs. normal  [29/118 (25%) vs. 45/896 (5%).  Abnormal waveforms associated more with proteinuria and intrauterine growth retardation. |
| Todros et al. (1995) [14] | Italy.  Screening study. N = 916 low risk pregnancies. | To assess the efficacy of the Doppler velocimetry of the uterine and umbilical arteries to predict pre-eclampsia and fetal growth retardation. | The umbilical and uterine artery systolic/diastolic ratios were significantly higher in the abnormal compared to the normal outcome group. The sensitivity with the uterine arteries at 19 to 24 weeks’ gestation was 59% for the detection of pregnancy-induced hypertension and 11% for small for gestational age infants. At 26 to 31 weeks, the values were 69% and 58%, respectively. |
| Valensise et al. (1993) [15] | Italy.  Prospective screening study. N = 272 primiparous women at 22 weeks’ gestation. | To assess the role of uterine artery Doppler flow velocity waveforms for predicting subsequent development of gestational hypertension and intrauterine fetal growth retardation. | The abnormal resistance index had a sensitivity of 74%, specificity of 97.5% and kappa index of 0.72 in predicting gestational hypertension. For intrauterine growth retardation, the values were 66%, 95% and 81, respectively. |

References

1. Papageorghiou AT, Yu CK, Cicero S, Bower S, Nicolaides KH: **Second-trimester uterine artery Doppler screening in unselected populations: a review**. *J Matern Fetal Neonatal Med* 2002, **12**(2):78-88.

2. Albaiges G, Missfelder-Lobos H, Lees C, Parra M, Nicolaides KH: **One-stage screening for pregnancy complications by color Doppler assessment of the uterine arteries at 23 weeks' gestation**. *Obstet Gynecol* 2000, **96**(4):559-564.

3. Aquilina J, Thompson O, Thilaganathan B, Harrington K: **Improved early prediction of pre-eclampsia by combining second-trimester maternal serum inhibin-A and uterine artery Doppler**. *Ultrasound Obstet Gynecol* 2001, **17**(6):477-484.

4. Bewley S, Cooper D, Campbell S: **Doppler investigation of uteroplacental blood flow resistance in the second trimester: a screening study for pre-eclampsia and intrauterine growth retardation**. *Br J Obstet Gynaecol* 1991, **98**(9):871-879.

5. Bower S, Schuchter K, Campbell S: **Doppler ultrasound screening as part of routine antenatal scanning: prediction of pre-eclampsia and intrauterine growth retardation**. *Br J Obstet Gynaecol* 1993, **100**(11):989-994.

6. Bower S, Bewley S, Campbell S: **Improved prediction of preeclampsia by two-stage screening of uterine arteries using the early diastolic notch and color Doppler imaging**. *Obstet Gynecol* 1993, **82**(1):78-83.

7. Frusca T, Soregaroli M, Valcamonico A, Guandalini F, Danti L: **Doppler velocimetry of the uterine arteries in nulliparous women**. *Early Hum Dev* 1997, **48**(1-2):177-185.

8. Harrington K, Cooper D, Lees C, Hecher K, Campbell S: **Doppler ultrasound of the uterine arteries: the importance of bilateral notching in the prediction of pre-eclampsia, placental abruption or delivery of a small-for-gestational-age baby**. *Ultrasound Obstet Gynecol* 1996, **7**(3):182-188.

9. Kurdi W, Campbell S, Aquilina J, England P, Harrington K: **The role of color Doppler imaging of the uterine arteries at 20 weeks' gestation in stratifying antenatal care**. *Ultrasound Obstet Gynecol* 1998, **12**(5):339-345.

10. Newnham JP, Patterson LL, James IR, Diepeveen DA, Reid SE: **An evaluation of the efficacy of Doppler flow velocity waveform analysis as a screening test in pregnancy**. *Am J Obstet Gynecol* 1990, **162**(2):403-410.

11. North RA, Ferrier C, Long D, Townend K, Kincaid-Smith P: **Uterine artery Doppler flow velocity waveforms in the second trimester for the prediction of preeclampsia and fetal growth retardation**. *Obstet Gynecol* 1994, **83**(3):378-386.

12. Papageorghiou AT, Yu CK, Bindra R, Pandis G, Nicolaides KH: **Multicenter screening for pre-eclampsia and fetal growth restriction by transvaginal uterine artery Doppler at 23 weeks of gestation**. *Ultrasound Obstet Gynecol* 2001, **18**(5):441-449.

13. Steel SA, Pearce JM, McParland P, Chamberlain GV: **Early Doppler ultrasound screening in prediction of hypertensive disorders of pregnancy**. *Lancet* 1990, **335**(8705):1548-1551.

14. Todros T, Ferrazzi E, Arduini D, Bastonero S, Bezzeccheri V, Biolcati M, Bonazzi B, Gabrielli S, Pilu GL, Rizzo G *et al*: **Performance of Doppler ultrasonography as a screening test in low risk pregnancies: results of a multicentric study**. *J Ultrasound Med* 1995, **14**(5):343-348.

15. Valensise H, Bezzeccheri V, Rizzo G, Tranquilli AL, Garzetti GG, Romanini C: **Doppler velocimetry of the uterine artery as a screening test for gestational hypertension**. *Ultrasound Obstet Gynecol* 1993, **3**(1):18-22.
