# Supplementary material for: Reducing stillbirths: screening and monitoring during pregnancy and labour
Source: BMC Pregnancy Childbirth. 2009 May 7;9(Suppl 1):S5. doi: 10.1186/1471-2393-9-S1-S5 (PMC2679411; doi:10.1186/1471-2393-9-S1-S5)
Supplement: Additional file 5 — Web Table 5. Component studies in Baschat 2004: impact of Doppler velocimetry on stillbirth and perinatal mortality in pre-term growth-restricted fetuses. Component studies in Baschat 2004 meta-analysis showing impact on stillbirths/perinatal mortality [file 1471-2393-9-S1-S5-S5.doc]

**Web Table 5. Component studies in Baschat 2004 [1]: impact of Doppler velocimetry on stillbirth and perinatal mortality in pre-term growth-restricted fetuses**

| **Source** | **Location and Type of Study** | **Intervention** | **Stillbirths / Perinatal Outcomes** |
| --- | --- | --- | --- |
| 1. Bilardo et al. 2004 [2] | Germany, Netherlands, UK.  Multi-centre prospective case study. Singleton growth-restricted fetuses delivered 26-33 weeks’ gestation for fetal distress who were monitored via Doppler until <24 hr before delivery (N=70). | Compared pregnancy outcomes among women with Doppler-diagnosed elevated ductus venosus (DV) index with normal DV index. | Antepartum SBR: 6/37 vs. 0/33 in elevated DV vs. normal DV groups, respectively. |
| 2. Ozcan et al. 1998 [3] | USA (Connecticut).  Prospective case study. Fetuses 26-32 weeks’ gestation with estimated fetal weight <5^th^ centile (N=19). | Compared pregnancy outcomes among women with Doppler-diagnosed DV reversed atrial velocity (RAV) vs. normal DV. | Antepartum SBR: 1/5 vs. 0/14 in DV-RAV vs. normal DV groups, respectively. |
| 3. Baschat et al. 2000 [4] | USA (Maryland).  Prospective case study. N=121 growth restricted fetuses with umbilical artery pulsatility >2 standard deviations above gestational age mean and birth weight <10^th^ centile. | Compared pregnancy outcomes among women with Doppler-diagnosed abnormal veins vs. normal DV. | Antepartum SBR: 11/50 vs. 0/71 in abnormal veins vs. normal DV groups, respectively. |
| 4. Muller et al. 2002 [5] | Germany (Wurzburg(.  Prospective case study. Singleton high-risk pregnancies (N=35) with umbilical absent or reverse end-diastolic flow (UA-A/REDV). | Compared pregnancy outcomes among women with Doppler diagnosed DV-RAV vs. UA A/REDV. | Antepartum SBR: 2/12 vs. 0/23 in DV-RAV vs UA-A/REDV groups, respectively. |
| 5. Baschat et al. 2003 [6] | USA (Maryland).  Prospective case study. Pre-term suspected growth-restricted fetuses before 37 weeks’ gestation (N=224). | Compared pregnancy outcomes among women with Doppler diagnosed abnormal veins vs. UA A/REDV. | Antenatal SB: 12/28 vs. 0/9 in abnormal veins vs. UA A/REDV groups, respectively. |
| 6. Figueras et al. 2003 [7] | Spain (Barcelona).  Retrospective case study. Singleton pregnancies (N=68) at or after 26 weeks of pregnancy in which delivery occurred within 3 days of Doppler surveillance. | Compared pregnancy outcomes among women with Doppler-diagnosed elevated DV index vs. normal DV index to contraction stress test results. | Antepartum SBR: 0/27 vs. 0/31 in elevated DV vs. normal DV groups, respectively. |
| 7. Hofstaetter C et al 2002 [8] | Germany (Bonn).  Prospective case study. Growth-restricted fetuses with reversed umbilical artery flow (N=37). | Compared pregnancy outcomes in women with Doppler-diagnosed abnormal veins vs. UA A/REDV. | Antepartum SBR: 12/28 vs. 0/9 in abnormal veins vs. UA A/REDV groups, respectively. |
| 8. Hofstaetter et al. 1996 [9] | Sweden (Malmo).  Prospective case study. High-risk complicated pregnancies (N=87) referred for umbilical artery Doppler assessment. | Compared pregnancy outcomes among women with elevated DV index vs. normal DV index. | Antepartum SBR: 0/22 vs. 0/65 in elevated DV vs. normal DV groups, respectively. |

**References**

1. Baschat AA: **Doppler application in the delivery timing of the preterm growth-restricted fetus: another step in the right direction**. *Ultrasound Obstet Gynecol* 2004, **23**(2):111-118.

2. Bilardo CM, Wolf H, Stigter RH, Ville Y, Baez E, Visser GH, Hecher K: **Relationship between monitoring parameters and perinatal outcome in severe, early intrauterine growth restriction**. *Ultrasound Obstet Gynecol* 2004, **23**(2):119-125.

3. Ozcan T, Sbracia M, d'Ancona RL, Copel JA, Mari G: **Arterial and venous Doppler velocimetry in the severely growth-restricted fetus and associations with adverse perinatal outcome**. *Ultrasound Obstet Gynecol* 1998, **12**(1):39-44.

4. Baschat AA, Gembruch U, Reiss I, Gortner L, Weiner CP, Harman CR: **Relationship between arterial and venous Doppler and perinatal outcome in fetal growth restriction**. *Ultrasound Obstet Gynecol* 2000, **16**(5):407-413.

5. Muller T, Nanan R, Rehn M, Kristen P, Dietl J: **Arterial and ductus venosus Doppler in fetuses with absent or reverse end-diastolic flow in the umbilical artery: correlation with short-term perinatal outcome**. *Acta Obstet Gynecol Scand* 2002, **81**(9):860-866.

6. Baschat AA, Gembruch U, Weiner CP, Harman CR: **Qualitative venous Doppler waveform analysis improves prediction of critical perinatal outcomes in premature growth-restricted fetuses**. *Ultrasound Obstet Gynecol* 2003, **22**(3):240-245.

7. Figueras F, Martinez JM, Puerto B, Coll O, Cararach V, Vanrell JA: **Contraction stress test versus ductus venosus Doppler evaluation for the prediction of adverse perinatal outcome in growth-restricted fetuses with non-reassuring non-stress test**. *Ultrasound Obstet Gynecol* 2003, **21**(3):250-255.

8. Hofstaetter C, Gudmundsson S, Hansmann M: **Venous Doppler velocimetry in the surveillance of severely compromised fetuses**. *Ultrasound Obstet Gynecol* 2002, **20**(3):233-239.

9. Hofstaetter C, Gudmundsson S, Dubiel M, Marsal K: **Ductus venosus velocimetry in high-risk pregnancies**. *Eur J Obstet Gynecol Reprod Biol* 1996, **70**(2):135-140.
