# Supplementary material for: Reducing stillbirths: screening and monitoring during pregnancy and labour
Source: BMC Pregnancy Childbirth. 2009 May 7;9(Suppl 1):S5. doi: 10.1186/1471-2393-9-S1-S5 (PMC2679411; doi:10.1186/1471-2393-9-S1-S5)
Supplement: Additional file 6 — Web Table 6. Component studies in Neilson et al. 2000 meta-analysis: Impact of Doppler screening during pregnancy. Component studies in Neilson et al. 2000 meta-analysis showing impact on stillbirths/perinatal mortality [file 1471-2393-9-S1-S5-S6.doc]

**Web Table 6. Component studies in Neilson et al. 2000 [1] meta-analysis: Impact of Doppler screening during pregnancy**

| **Source** | **Location and Type of Study** | **Intervention** | **Stillbirths / Perinatal Outcomes** |
| --- | --- | --- | --- |
| 1. Biljan et al. 1992 [2] | UK (Chester).  RCT. N=704 women with high-risk singleton pregnancies. | Compared the impact of Doppler (intervention) vs. no Doppler (controls). | SBR: OR=0.51 (95% CI: 0.05-4.91) **[NS]**  [1/338 vs. 2/366 in intervention vs. control groups, respectively]. |
| 2. Burke et al. 1992 [3] | Ireland (Dublin).  RCT. N=476 women referred for fetal assessment. | To assess the effect of Doppler (intervention) vs. no Doppler (controls) (0.8% of controls underwent Doppler examination). | SBR: OR=1.46 (95% CI: 0.25-8.49) **[NS]**  [3/241 vs. 2/235 in intervention vs. control groups, respectively]. |
| 3. Johnstone et al. 1993 [4] | UK (Edinburgh, Scotland).  RCT. N=2329 participants. | Compared the impact of Doppler (intervention) vs. no Doppler (controls) (0.2% of controls underwent Doppler examination). | SBR: OR=1.06 (95% CI: 0.26-4.24) **[NS]**  [4/1132 vs. 4/1197 in intervention vs. control groups, respectively]. |
| 4. Tyrrell et al. [5] | UK (Leeds).  RCT. N=500 participants. | Assessed the impact on stillbirths of Doppler plus modified biophysical profile exam (intervention) vs no Doppler (controls) (4.8% of controls had Doppler for clinical indications. | SBR: OR=2.73 (95% CI: 0.38-19.53) **[NS]**  [3/250 vs. 1/250 in intervention vs. control groups, respectively]. |
| 5. Neales et al. 1994 [6] | UK (London).  RCT. N=467 women at 24 wks or more with a singleton pregnancy with a fetus with an abdominal circumference <5th percentile on ultrasound measurement. | Compared the impact of Doppler revealed (intervention) vs. Doppler concealed (controls). | SBR: OR=0.65 (95% CI: 0.23-1.81) **[NS]**  [6/236 vs. 9/231 in intervention vs. control groups, respectively]. |
| 6. Nienhuis et al. 1995[7, 8] | Netherlands (Maastricht).  RCT. N=150 women with singleton pregnancies with clinical suspicion of fetal growth restriction. | Compared the impact of Doppler (intervention) vs. no Doppler (controls) (39% of controls underwent Doppler examination). | SBR: OR=0.37 (95% CI: 0.05-2.68) **[NS]**  [1/74 vs. 3/76 in intervention vs. control groups, respectively]. |
| 7. Hofmeyr et al. 1991 [9] | UK (Oxford).  RCT. N=897 women with high risk pregnancies. | Compared the impact of Doppler (intervention) vs. no Doppler (controls). | SBR: OR=0.54 (95% CI: 0.11-2.67) **[NS]**  [2/438 vs. 4/459 in intervention vs. control groups, respectively]. |
| 8. Newnham et al. 1991 [10] | UK (Perth, Scotland).  RCT. N=545 participants. | Compared the impact of Doppler (intervention) vs. no Doppler (controls). | SBR: OR=1.47 (95% CI: 0.25-8.53) **[NS]**  [3/275 vs. 2/270 in intervention vs. control groups, respectively].  PMR: OR=0.98 (95% CI: 0.38-2.51) **[NS]**  [9/275 vs. 9/270 in intervention vs. control groups, respectively]. |
| 9. Almstrom et al. 1992 [11] [12] | Sweden.  RCT. N=426 women with singleton pregnancies with ultrasound evidence of SGA at or after 31 wks gestation. | Compared the impact of Doppler (intervention) vs. cardiotocography (controls). Doppler was not used in the control pregnancies. | SBR: OR=0.13 (95% CI: 0.01-2.14) **[NS]**  [0/214 vs. 2/212 in intervention vs. control groups, respectively]. |
| 10. Trudinger et al. [13] | Australia (Sydney).  RCT. N=289 women with singleton pregnancies who had been admitted to the antenatal ward after 28 wks of gestation. | Compared the impact on stillbirths of revealed Doppler (intervention) vs. concealed Doppler (controls). | SBR: OR=0.65 (95% CI: 0.07-6.39) **[NS]**  [1/127 vs. 2/162 in intervention vs. control groups, respectively]. |
| 11. Pattinson et al. 1994 [14, 15] | South Africa (Tygerberg).  RCT. N=212 women. | Compared the impact of revealed Doppler (intervention) vs. concealed Doppler (controls). | PMR: OR=0.82 (95% CI: 0.27-2.50) **[NS]**  [6/108 vs. 7/104 in intervention vs. control groups, respectively]. |

References

1. Neilson JP, Z Alfirevic: **Doppler ultrasound for fetal assessment in high risk pregnancies**. *Cochrane Database of Systematic Reviews;* 2000(4):CD000073.

2. Biljan M, Haddad N, McVey K, Williams J: **Efficiency of continuous-wave Doppler in screening high risk pregnancies in a district general hopital (a prospective randomized study on 674 singleton pregnancies).** In: *Proceedings of 26th British Congress of Obstetrics and Gynaecology: 1992; Manchester, UK.*; 1992.

3. Burke G, Stuart B, Crowley P, Ni Scanaill S, Drumm J: **Does Doppler ultrasound alter the management of high risk pregnancy? Care, concern and cure in perinatal medicine.** In: *Carnforth: Parthenon: 1992 May.; Amsterdam.*; 1992 May.: 597-604.

4. Johnstone FD, Prescott R, Hoskins P, Greer IA, McGlew T, Compton M: **The effect of introduction of umbilical Doppler recordings to obstetric practice**. *Br J Obstet Gynaecol* 1993, **100**(8):733-741.

5. Tyrrell SN, Lilford RJ, Macdonald HN, Nelson EJ, Porter J, Gupta JK: **Randomized comparison of routine vs highly selective use of Doppler ultrasound and biophysical scoring to investigate high risk pregnancies**. *Br J Obstet Gynaecol* 1990, **97**(10):909-916.

6. Neales K, Motteram J, Maxwell D: **A randomised controlled trial to assess the use of Doppler ultrasound in the management of patients with intrauterine growth retardation. Personal communication.** In*.*; 1992.

7. Nienhuis S: **Costs and effects of Doppler ultrasound measurements in suspected intrauterine growth retardation. A randomised clinical trial** Maastricht: Universitaire Pers Maastrich; 1995.

8. Nienhuis SJ, Ruissen CJ, Hoogland HJ, Gerver JW, Vles J, de Haan J: **Cost-effectiveness of a Doppler policy in suspected intrauterine growth retardation - a randomized controlled trial**. In: *Proceedings of the XIII World Congress of Gynecology and Obstetrics (FIGO): 1991; Singapore*; 1991.

9. Hofmeyr GJ, Pattinson R, Buckley D, Jennings J, Redman CW: **Umbilical artery resistance index as a screening test for fetal well-being. II: Randomized feasibility study**. *Obstet Gynecol* 1991, **78**(3 Pt 1):359-362.

10. Newnham JP, O'Dea MR, Reid KP, Diepeveen DA: **Doppler flow velocity waveform analysis in high risk pregnancies: a randomized controlled trial**. *Br J Obstet Gynaecol* 1991, **98**(10):956-963.

11. Almstrom H, Axelsson O, Cnattingius S, Ekman G, Maesel A, Ulmsten U, Arstrom K, Marsal K: **Comparison of umbilical-artery velocimetry and cardiotocography for surveillance of small-for-gestational-age fetuses**. *Lancet* 1992, **340**(8825):936-940.

12. Marsal K, Almstrom H, Axelsson O, Cnattingius S, Ekman G, al e: **Umbilical artery velocimetry is more effective than cardiotocography for surveillance of growth retarded fetuses**. *Journal of Perinatal Medicine;* 1991, **2**:84S.

13. Trudinger BJ, Cook CM, Giles WB, Connelly A, Thompson RS: **Umbilical artery flow velocity waveforms in high-risk pregnancy. Randomised controlled trial**. *Lancet* 1987, **1**(8526):188-190.

14. Pattinson RC, Norman K, Odendaal HJ: **The role of doppler velocimetry in the management of pregnancies: a randomized controlled trial.** In: *Proceedings of 11th Conference on Priorities in Perinatal Care in South Africa: 1992 March 12-15.; Caledon, South Africa.*; 1992 March 12-15.: 59-63.

15. Pattinson RC, Norman K, Odendaal HJ: **The role of Doppler velocimetry in the management of high risk pregnancies**. *Br J Obstet Gynaecol* 1994, **101**(2):114-120.
