# Supplementary material for: Reducing stillbirths: screening and monitoring during pregnancy and labour
Source: BMC Pregnancy Childbirth. 2009 May 7;9(Suppl 1):S5. doi: 10.1186/1471-2393-9-S1-S5 (PMC2679411; doi:10.1186/1471-2393-9-S1-S5)
Supplement: Additional file 7 — Web Table 7. Component studies in Pattinson et al. 1997 meta-analysis: Impact of pelvimetry during pregnancy. Component studies in Pattinson et al. 1997 meta-analysis showing impact on stillbirths/perinatal mortality [file 1471-2393-9-S1-S5-S7.doc]

**Web Table 7. Component studies in Pattinson et al. 1997 [1] meta-analysis: Impact of pelvimetry during pregnancy**

| **Source** | **Location and Type of Study** | **Intervention** | **Stillbirths / Perinatal Outcomes** |
| --- | --- | --- | --- |
| 1. Crichton 1962 [2] | South Africa (Cape Town).  RCT. N=305 women in labour whose attending doctors requested radiography. | To assess the impact of X-ray pelvimetry on pelvic adequacy (intervention). | PMR: OR=0.63 (95% CI: 0.21-1.91) **[NS]**  [5/151 vs. 8/154 in intervention vs. control groups, respectively]. |
| 2. Parsons et al. 1985 [3] | USA.  RCT. N=300 primigravidae with vertex presentations to receive oxytocin for induction of labour or augmentation of labour. | To assess the impact of clinical and/or x-ray pelvimetry, after which a decision was made on the use of oxytocin or caesarean section (intervention). | PMR: OR not estimable.  [0/102 vs. 0/98 in intervention vs. control groups, respectively]. |
| 3. Richards et al. 1985 [4] | South Africa.  RCT. N=102 women with prior caesarean section. | To assess the impact of X-ray pelvimetry (intervention). In the intervention group, pelvimetry was performed at 36 wks' gestation. If the pelvic inlet was < 10.5 cm in the antero-posterior diameter or < 11.5 cm in the transverse diameter, an elective caesarean section was performed. A trial of scar was performed on the rest. In the control group, all women had a trial of scar with expectant management. | PMR: OR=0.13 (95% CI: 0.01-2.07) **[NS]**  [0/52 vs. 2/50 in intervention vs. control groups, respectively]. |
| 4. Thubisi et al. 1993 [5] | South Africa (Congella).  RCT. N=153 pregnant women with prior caesarean section. | Assessed the impact on perinatal mortality of intervention with X-ray pelvimetry at 36 wks. A sagittal inlet < 11 cm, sagittal outlet < 10 cm, transverse inlet < 11.5 cm, and transverse outlet (bispinous) < 9 cm was an indication for caesarean section. The remainder of the group and the control group underwent a trial of scar with expectant management. | PMR: OR not estimable.  [0/144 in both groups]. |

References

1. Pattinson RC, E Farrell: **Pelvimetry for fetal cephalic presentations at or near term**. *Cochrane Database of Systematic Reviews;* 1997(2):CD000161.

2. Crichton D: **The accuracy and value of cephalopelvimetry**. *Journal of Obstetrics and Gynaecology of the British Commonwealth;* 1962, **69**:366-378.

3. Parsons MT, Spellacy WN: **Prospective randomized study of x-ray pelvimetry in the primigravida**. *Obstet Gynecol* 1985, **66**(1):76-79.

4. Richards A, Strang A, Moodley J, Philpott H: **Vaginal delivery following caesarean section - is X-ray pelvimetry a reliable predictor?** In: *Proceedings of 4th Conference on Priorities in Perinatal Care in South Africa: 1985.; Natal, South Africa.*; 1985.: 62-65.

5. Thubisi M, Ebrahim A, Moodley J, Shweni PM: **Vaginal delivery after previous caesarean section: is X-ray pelvimetry necessary?** *Br J Obstet Gynaecol* 1993, **100**(5):421-424.
