# Supplementary material for: Reducing stillbirths: screening and monitoring during pregnancy and labour
Source: BMC Pregnancy Childbirth. 2009 May 7;9(Suppl 1):S5. doi: 10.1186/1471-2393-9-S1-S5 (PMC2679411; doi:10.1186/1471-2393-9-S1-S5)
Supplement: Additional file 8 — Web Table 8. Component studies in Russell et al. 2007 meta-analysis: Impact of management of gestational diabetes on stillbirth and perinatal outcomes. Component studies in Russell et al. 2007 showing impact on stillbirths/perinatal mortality [file 1471-2393-9-S1-S5-S8.doc]

### Web Table 8. Component studies in Russell et al. 2007 [1]: Impact of management of gestational diabetes on stillbirth and perinatal outcomes

| **Source** | **Location and Type of Study** | **Intervention** | **Stillbirths / Perinatal Outcomes** |
| --- | --- | --- | --- |
| Crowther et al 2005 [2] | Australia.  RCT. Pregnant women (N=1000; N=490 intervention, N=510 controls) 24-34 wks’ gestation with gestational diabetes. | Compared the impact of dietary advice, blood glucose monitoring, and insulin therapy as needed (intervention) vs. routine care (controls). | SBR: 0/490 vs. 3/510 in intervention vs. control groups, respectively (P=0.26)  PMR: 0/490 vs. 5/510 in intervention vs. control groups, respectively (P=0.07).  NMR: 0/490 vs. 2/510 in intervention vs. control groups, respectively (P=0.50). |

References

1. Russell MA, Carpenter MW, Coustan DR: **Screening and diagnosis of gestational diabetes mellitus**. *Clin Obstet Gynecol* 2007, **50**(4):949-958.

2. Crowther CA, Hiller JE, Moss JR, McPhee AJ, Jeffries WS, Robinson JS: **Effect of treatment of gestational diabetes mellitus on pregnancy outcomes**. *N Engl J Med* 2005, **352**(24):2477-2486.
