# Supplementary material for: Reducing stillbirths: screening and monitoring during pregnancy and labour
Source: BMC Pregnancy Childbirth. 2009 May 7;9(Suppl 1):S5. doi: 10.1186/1471-2393-9-S1-S5 (PMC2679411; doi:10.1186/1471-2393-9-S1-S5)
Supplement: Additional file 9 — Web Table 9. Component studies in Mukhopadhyay et al. 2007: Impact of continuous subcutaneous insulin infusion on intrauterine fetal death. Component studies in Mukhopadhyay et al. 2007 showing impact on stillbirths/perinatal mortality [file 1471-2393-9-S1-S5-S9.doc]

**Web Table 9. Component studies in Mukhopadhyay et al. 2007 [1]: Impact of continuous subcutaneous insulin infusion on intrauterine fetal death**

| **Source** | **Location and Type of Study** | **Intervention** | **Stillbirths / Perinatal Outcomes** |
| --- | --- | --- | --- |
| Botta et al. 1986 [2] | RCT. N = 10 pregnant women with type I diabetes mellitus at second or third month of pregnancy. | Compared the impact of continuous subcutaneous insulin infusion (intervention) vs. intensified traditional insulin therapy (controls). | Fetal death (miscarriage + SB): OR = 2.25 (95% CI: 0.02 – 215.09) **[NS]**  [1/5 vs. 0/5 in intervention and control groups, respectively] |
| Burkart et al. 1988 [3] | Germany.  RCT. N = 189 pregnant diabetic women (N = 48 intervention group, N = 41 controls). | Compared the impact of continuous subcutaneous insulin infusion (intervention) vs. intensified conventional treatment (controls). | Fetal death (miscarriage + SB): OR = 1.72 (95% CI: 0.01 – 138.30) **[NS]**  [1/48 vs. 0/41 in intervention and control groups, respectively] |
| Carta et al. 1993 [4] | RCT. N = 15 pregnant women with type I and 14 with type II. | Compared the impact of continuous subcutaneous insulin infusion (intervention) vs. intensive conventional insulin therapy (controls). | Fetal death (miscarriage + SB): OR = 4.83 (95% CI: 0.14, infinity)  [2/14 vs. 0/15 in intervention and control groups, respectively] |
| Coustan et al. 1988 [5] | USA.  RCT. N = 22 pregnant diabetic women. | Compared the impact of insulin pump therapy (intervention) vs. intensive conventional insulin therapy (controls). | Fetal death (miscarriage + SB): OR = excluded  [0/11 vs. 0/11 in intervention and control groups, respectively] |
| Nosari et al. 1993 [6] | **Full text not available.** |  | Fetal death (miscarriage + SB): OR = 2.14 (95% CI: 0.09 – 134.70) **[NS]**  [2/16 vs. 1/16 in intervention and control groups, respectively] |

References

1. Mukhopadhyay A, Farrell T, Fraser RB, Ola B: **Continuous subcutaneous insulin infusion vs intensive conventional insulin therapy in pregnant diabetic women: a systematic review and metaanalysis of randomized, controlled trials**. *Am J Obstet Gynecol* 2007, **197**(5):447-456.

2. Botta RM, Sinagra D, Angelico MC, Bompiani GD: **[Comparison of intensified traditional insulin therapy and micropump therapy in pregnant women with type 1 diabetes mellitus]**. *Minerva Med* 1986, **77**(17):657-661.

3. Burkart W, Hanker JP, Schneider HP: **Complications and fetal outcome in diabetic pregnancy. Intensified conventional versus insulin pump therapy**. *Gynecol Obstet Invest* 1988, **26**(2):104-112.

4. Carta Q, Meriggi E, Trossarelli GF, Catella G, Dal Molin V, Menato G, Gagliardi L, Massobrio M, Vitelli A: **Continuous subcutaneous insulin infusion versus intensive conventional insulin therapy in type I and type II diabetic pregnancy**. *Diabete Metab* 1986, **12**(3):121-129.

5. Coustan DR, Reece EA, Sherwin RS, Rudolf MC, Bates SE, Sockin SM, Holford T, Tamborlane WV: **A randomized clinical trial of the insulin pump vs intensive conventional therapy in diabetic pregnancies**. *JAMA* 1986, **255**(5):631-636.

6. Nosari I, Maglio ML, Lepore G, Cortinovis F, Pagani G: **Is continous subcutaneous insulin infusion more effective than conventional insulin therapy in the treatment of pregnant diabetic women?** *Diabetes Nutr Metab* 1993, **6**:33-37.
