# Supplementary material for: Reducing stillbirths: screening and monitoring during pregnancy and labour
Source: BMC Pregnancy Childbirth. 2009 May 7;9(Suppl 1):S5. doi: 10.1186/1471-2393-9-S1-S5 (PMC2679411; doi:10.1186/1471-2393-9-S1-S5)
Supplement: Additional file 10 — Web Table 10. Component studies in Boulvain et al. 2001 meta-analysis: Impact of elective delivery on perinatal mortality. Component studies in Boulvain et al. 2001 showing impact on stillbirths/perinatal mortality [file 1471-2393-9-S1-S5-S10.doc]

**Web Table 10. Component studies in Boulvain et al. 2001 [1]: Impact of elective delivery on perinatal mortality**

| **Source** | **Location and Type of Study** | **Intervention** | **Stillbirths / Perinatal Outcomes** |
| --- | --- | --- | --- |
| 1. Kjos et al. 1993 [2] | USA.  RCT. N=200 women. (N=100 intervention group, N=controls). | Compared the impact on PMR of active induction of labour by IV oxytocin < 5 days (for pregnancies with unclear gestational age, amniocentesis was performed, and induction delayed until lecithin/sphingomyelin (L/S) ratio ≥ 2.0) vs. expectant management until 42 wks with twice-weekly antenatal testing and weekly consultation. Induction of labour indicated by fetal distress, pre-eclampsia, poor metabolic control (see above definition), estimated fetal weight > 4200 g, or term > 42 wks (294 days). | PMR: RR not estimable **[NS]**  [0/100 in both groups]. |

References

1. Boulvain M, Stan C, Irion O: **Elective delivery in diabetic pregnant women**. *Cochrane Database Syst Rev* 2001(2):CD001997.

2. Kjos SL, Henry OA, Montoro M, Buchanan TA, Mestman JH: **Insulin-requiring diabetes in pregnancy: a randomized trial of active induction of labor and expectant management**. *Am J Obstet Gynecol* 1993, **169**(3):611-615.
