# Supplementary material for: Reducing stillbirths: screening and monitoring during pregnancy and labour
Source: BMC Pregnancy Childbirth. 2009 May 7;9(Suppl 1):S5. doi: 10.1186/1471-2393-9-S1-S5 (PMC2679411; doi:10.1186/1471-2393-9-S1-S5)
Supplement: Additional file 11 — Web Table 11. Component studies in Pattison and McCowan 1999 meta-analysis: Impact of cardiotocography for antepartum fetal assessment on perinatal mortality. Component studies in Pattison and McCowan 1999 showing impact on stillbirths/perinatal mortality [file 1471-2393-9-S1-S5-S11.doc]

**Web Table 11. Component studies in Pattison and McCowan 1999 meta-analysis [1]: Impact of cardiotocography for antepartum fetal assessment on perinatal mortality**

| **Source** | **Location and Type of Study** | **Intervention** | **Stillbirths / Perinatal Outcomes** |
| --- | --- | --- | --- |
| 1. Brown 1982 [2] | UK (Sheffield). Jessop Hospital for Women and Northern General Hospital.  RCT. N=353 women (N=171 intervention group, N=182 controls. | Compared the impact of antenatal CTG monitored group and revealed (intervention) vs. monitored but concealed group (control).  Antenatal CTG not practiced routinely, biochemical tests and ultrasound used in both groups. | PMR (non lethal): OR=6.99 (95% CI: 0.44-112.41) **[NS]**.  [2/182 vs. 0/171 in intervention and control groups, respectively]. |
| 2. Kidd et al. 1985 | UK (Dundee). Ninewells Hospital and Medical School.  Quasi-RCT. N=396 women (N=198 intervention, N=198 controls). | Compared the impact of antenatal CTG monitored and revealed (intervention) vs. monitored but concealed group (control). | PMR (non lethal): OR=7.46 (95% CI: 0.77-72.18) **[NS]**.  [3/198 vs. 0/198 in intervention and control groups, respectively]. |
| 3. Lumley et al. 1993 [4] | Australia (Melbourne). Queen Victoria Medical Centre.  RCT. N=539 women (N=274 women intervention group, N=265 controls). | Compared the impact on perinatal mortality of antenatal CTG monitored group (intervention) vs. non monitored group (controls). | PMR (non lethal): OR=1.67 (95% CI: 0.50-5.49) **[NS]**.  [7/271 vs. 4/259 in intervention and control groups, respectively]. |

References

1. Pattison N, McCowan L: **Cardiotocography for antepartum fetal assessment**. *Cochrane Database of Systematic Reviews;* 1999(1):CD001068.

2. Brown VA, Sawers RS, Parsons RJ, Duncan SL, Cooke ID: **The value of antenatal cardiotocography in the management of high-risk pregnancy: a randomized controlled trial**. *Br J Obstet Gynaecol* 1982, **89**(9):716-722.

3. Kidd LC, Patel NB, Smith R: **Non-stress antenatal cardiotocography--a prospective randomized clinical trial**. *Br J Obstet Gynaecol* 1985, **92**(11):1156-1159.

4. Lumley J, Lester A, Anderson I, Renou P, Wood C: **A randomised trial of weekly cardiotocography in high risk obstetric patients**. *Br J Obstet Gynaecol;* 1993, **90**:1018-1026.
