# Supplementary material for: Reducing stillbirths: screening and monitoring during pregnancy and labour
Source: BMC Pregnancy Childbirth. 2009 May 7;9(Suppl 1):S5. doi: 10.1186/1471-2393-9-S1-S5 (PMC2679411; doi:10.1186/1471-2393-9-S1-S5)
Supplement: Additional file 12 — Web Table 12. Component studies in Lalor et al. 2008 meta-analysis: Impact of fetal biophysical profile. Component studies in Lalor et al. 2008 showing impact on stillbirths/perinatal mortality [file 1471-2393-9-S1-S5-S12.doc]

**Web Table 12. Component studies in Lalor et al. 2008 meta-analysis [1]: Impact of fetal biophysical profile**

| **Source** | **Location and Type of Study** | **Intervention** | **Stillbirths / Perinatal Outcomes** |
| --- | --- | --- | --- |
| 1. Manning et al. 1984 [2] | Canada.  RCT. N=735 high-risk women. (N=375 intervention group, N=360 controls). | Compared the impact on PMR of weekly BPP reported to physician (intervention) vs. weekly BPP with only results of cardiotocography (NST) given to physician; full BPP concealed (controls). BPP performed twice weekly for diabetic and post-term mothers in both intervention and control groups. | PMR (including major malformations): RR=0.96 (95% CI: 0.24-3.81) **[NS]**  [4/375 vs. 4/360 in intervention vs. control groups, respectively].  PMR: OR=0.96 (95% CI: 0.24-3.86) **[NS]**  [4/375 vs. 4/360 in intervention vs. control groups, respectively]. |
| 2. Nageotte et al. 1994 [3] | USA (California).  RCT. N=1,307 women (N=679 intervention group, N=628 controls). | Compared the impact on perinatal mortality in the intervention group (every abnormal 'modified' BPP was followed by full BPP as a back up test) vs. control group in which every abnormal 'modified' BPP was followed by a CST as a back up test. | PMR (including major malformations): RR=1.54 (95% CI: 0.37-6.42) **[NS]**  [5/679 vs. 3/628 in intervention vs. control groups, respectively].  PMR (excluding major malformations): OR=3.09 (95% CI: 0.53-17.89) **[NS]**  [4/679 vs. 1/628 in intervention vs. control groups, respectively]. |
| 3. Platt et al. 1985 [4] | USA.  Quasi-RCT. N=652 pregnant women (N=279 intervention, N=373 controls). | Compared the impact on PMR of twice-weekly BPP (intervention) vs. twice-weekly cardiotocography (NST) plus maximum pool depth measurement (controls). Labour induced for abnormal results, otherwise managed expectantly until 43 weeks. | PMR (including major malformations): RR=1.34 (95% CI: 0.27-6.57) **[NS]**  [3/279 vs. 3/373 in intervention vs. control groups, respectively].  PMR (excluding major malformations): OR=0.89 (95% CI: 0.25-3.14) **[NS]**  [4/279 vs. 6/373 in intervention vs. control groups, respectively]. |
| 4. Alfirevic et al. 1995 [5] | UK (London).  RCT. N=145 women (N=72 intervention group, N=73 controls). | Compared the impact on PMR of twice-weekly BPP (intervention) vs. twice-weekly cardiotocography (NST) plus maximum pool depth measurement (controls). Labour induced for abnormal results, otherwise managed expectantly until 43 weeks. | PMR (including major malformations): RR=3.04 (95% CI: 0.13-73.44) **[NS]**  [1/72 vs. 0/73 in intervention vs. control groups, respectively].  PMR (excluding major malformations: OR=7.49 (95% CI: 0.15-377.63) **[NS]**  [1/72 vs. 0/73 in intervention vs. control groups, respectively]. |

References

1. Lalor JG, Fawole B, Alfirevic Z, Devane D: **Biophysical profile for fetal assessment in high risk pregnancies**. *Cochrane Database Syst Rev* 2008(1):CD000038.

2. Manning FA, Lange IR, Morrison I, Harman CR: **Fetal biophysical profile score and the nonstress test: a comparative trial**. *Obstet Gynecol* 1984, **64**(3):326-331.

3. Nageotte MP, Towers CV, Asrat T, Freeman RK: **Perinatal outcome with the modified biophysical profile**. *Am J Obstet Gynecol* 1994, **170**(6):1672-1676.

4. Platt LD, Walla CA, Paul RH, Trujillo ME, Loesser CV, Jacobs ND, Broussard PM: **A prospective trial of the fetal biophysical profile versus the nonstress test in the management of high-risk pregnancies**. *Am J Obstet Gynecol* 1985, **153**(6):624-633.

5. Alfirevic Z, Walkinshaw SA: **A randomised controlled trial of simple compared with complex antenatal fetal monitoring after 42 weeks of gestation**. *Br J Obstet Gynaecol* 1995, **102**(8):638-643.
