# Supplementary material for: Reducing stillbirths: screening and monitoring during pregnancy and labour
Source: BMC Pregnancy Childbirth. 2009 May 7;9(Suppl 1):S5. doi: 10.1186/1471-2393-9-S1-S5 (PMC2679411; doi:10.1186/1471-2393-9-S1-S5)
Supplement: Additional file 13 — Web Table 13. Component studies in Tan et al. 2001 meta-analysis: Impact of vibroacoustic stimulation. Component studies in Tan et al. 2001 meta-analysis showing impact on stillbirths/perinatal mortality [file 1471-2393-9-S1-S5-S13.doc]

**Web Table 13. Component studies in Tan et al. 2001 meta-analysis [1]: Impact of vibroacoustic stimulation**

| **Source** | **Location and Type of Study** | **Intervention** | **Stillbirths / Perinatal Outcomes** |
| --- | --- | --- | --- |
| 1.Marden et al. 1997 [2] | USA (Colorado).  RCT. N=577 women of at least 31 wks' gestation. | Compared the impact on pregnancy outcomes of vibroacoustic stimulation (3 sec at the midpoint between the maternal pubic symphysis and umbilicus, with fetal movements palpated by hand at fundus; intervention) vs. mock stimulation with manual fetal movement monitoring (controls). | Non-reactive cardiotocography test: RR=1.02 (95% CI: 0.49-2.12) **[NS]**  [14/297 vs. 13/280 in intervention vs. control groups, respectively]. |
| 2.Marquez et al. 1993 [3] | Mexico.  RCT. N=180 women ≥ 32 wks' gestation. | Compared the impact on pregnancy outcomes of vibroacoustic stimulation (5 sec; intervention) vs. mock or no stimulation (controls). | Non-reactive cardiotocography test: RR=0.30 (95% CI: 0.09-1.05) **[NS]**  [3/90 vs. 10/90 in intervention vs. control groups, respectively]. |
| 3. Newnham et al. 1990 [4] | Western Australia.  RCT. N=300 women ≥ 34 wks' gestation. Excluded if CST contraindicated. | Assessed the impact on pregnancy outcomes of randomisation to vibroacoustic stimulation in cases where 5 min cardiotocographic monitoring was nonreactive (3 sec maternal abdominal stimulation near fetal head, repeated at 1-min intervals if fetal heart rate tachycardia was not observed (intervention), vs. manual stimulation in cases where 20 min cardiotocographic monitoring was nonreactive (stimulation for 20 min, plus food, plus nipple stimulation CST if fetus remained non-reactive 20 more min; controls). | PMR: RR not estimable. [NS]  [0/82 vs. 0/90 in intervention vs. control groups, respectively]. |
| 4.Perez-Delboy et al. 2002 [5] | USA (New York).  RCT. N=113 women. | Compared the impact on pregnancy outcomes of vibroacoustic stimulation (1 sec on maternal abdomen, repeated for 2 and then 3 seconds at 10 min intervals if nonreactive; intervention), vs. traditional non-stress test (controls). | Non-reactive cardiotocography test: RR=0.08 (95% CI: 0.00-1.37) **[NS]**  [0/61 vs. 5/92 in intervention vs. control groups, respectively]. |
| 5. Salamalekis et al. 1995. [6] | Greece.  RCT. N=225 women. | Assessed the impact on pregnancy outcomes of vibroaccoustic stimulation for nonreactive charts after monitoring for 5 minutes (4 one-second pulses, 1 second between pulses on maternal abdomen near fetal head; intervention), vs non-stress monitoring for 20 minutes, continued 20 more minutes for non-reactive cases (controls). | PMR: RR not estimable. [NS]  [0/110 vs. 0/115 in intervention vs. control groups, respectively].  Non reactive cardiotocography test: RR=1.39 (95% CI: 0.69-2.81) **[NS]**  [16/110 vs. 12/115 in intervention vs. control groups, respectively]. |
| 6.Saracoglu et al. 1999 [7, 8] | Turkey. Perinatology Unit.  RCT. N=400. | Compared the impact on pregnancy outcomes of vibroacoustic stimulation (1 sec up to 4 times on maternal abdomen near fetal head) for nonreactive charts after 5 minutes NST monitoring (intervention), vs. controls. | Non reactive cardiotocography test: RR=0.61 (95% CI: 0.37-1.00) **[NS]**  [22/200 vs. 36/200 in intervention vs. control groups, respectively]. |
| 7.Smith et al. 1986 [9] | USA (California).  RCT. N=1715 women. | Compared the effect on pregnancy outcomes of vibroacoustic stimulation (3 sec up to 3 times at 1-minute intervals on maternal abdomen near fetal head) for nonreactive charts after 5 minutes NST monitoring (intervention) vs. NST (controls). | PMR: RR=0.32 (95% CI: 0.01-7.78). [NS]  [0/366 vs. 1/349 in intervention vs. control groups, respectively].  Non reactive cardiotocography test: RR=0.65 (95% CI: 0.50-0.85) **[NS]**  [78/851 vs. 122/864 in intervention vs. control groups, respectively]. |
| 8.Tongsong et al. 1994 [10] | Thailand.  RCT. N=1273 women. | Compared the impact on pregnancy outcomes of vibroacoustic stimulation (1 sec, repeated up to 3 times if no fetal heart acceleration within 15 seconds, cycle repeated if reactive test not obtained in 10 mins, and monitoring extended for 20 mins if not reactive in first 20 mins; intervention), vs. standard non-stress testing, extended 20 mins if not reactive in first 20 mins. | Non reactive cardiotocography test: RR=0.49 (95% CI: 0.35-0.70) **[NS]**  [43/635 vs. 88/638 in intervention vs. control groups, respectively]. |

References

1. Tan KH, Smyth R: **Fetal vibroacoustic stimulation for facilitation of tests of fetal wellbeing**. *Cochrane Database Syst Rev* 2001(1):CD002963.

2. Marden D, McDuffie RS, Jr., Allen R, Abitz D: **A randomized controlled trial of a new fetal acoustic stimulation test for fetal well-being**. *Am J Obstet Gynecol* 1997, **176**(6):1386-1388.

3. Marquez TL, Andrade EH, Goldsmit DM, De La Huerta MIP, Garcia RBL: **The value of antepartum fetal heart rate with vibroacoustic stimulation (translation)**. *Ginecologia y Obstetricia de Mexico;* 1993, **61**:356-359.

4. Newnham JP, Burns SE, Roberman BD: **Effect of vibratory acoustic stimulation on the duration of fetal heart rate monitoring tests**. *Am J Perinatol* 1990, **7**(3):232-234.

5. Perez-Delboy A, Weiss J, Michels A, Cleary J, Shevell T, Malone F: **A randomized trial of vibroacoustic stimulation for antenatal fetal testing** *American Journal of Obstetrics and Gynecology;* 2002, **187**:S146.

6. Salamalekis E, Batalias L, Kassanos D, Loghis C, Pyrgiotis E, Zourlas PA: **The acoustic stimulation test and antenatal cardiotocography as diagnostic tools in high risk pregnancies**. *Journal of Obstetrics and Gynaecology;* 1995, **15**:292-294.

7. Saracoglu F, Gol K, Sahin I, Turkkani T, Atlay C: **The predictive value of fetal acoustic stimulation**. *Prenatal and Neonatal Medicine* 1998, **3**(Suppl 1):64.

8. Saracoglu F, Gol K, Sahin I, Turkkani B, Oztopcu C: **The predictive value of fetal acoustic stimulation**. *J Perinatol* 1999, **19**(2):103-105.

9. Smith CV, Phelan JP, Platt LD, Broussard P, Paul RH: **Fetal acoustic stimulation testing. II. A randomized clinical comparison with the nonstress test**. *Am J Obstet Gynecol* 1986, **155**(1):131-134.

10. Tongsong T, Piyamongkol W: **Comparison of the acoustic stimulation test with nonstress test. A randomized, controlled clinical trial**. *J Reprod Med* 1994, **39**(1):17-20.
