# Supplementary material for: Reducing stillbirths: screening and monitoring during pregnancy and labour
Source: BMC Pregnancy Childbirth. 2009 May 7;9(Suppl 1):S5. doi: 10.1186/1471-2393-9-S1-S5 (PMC2679411; doi:10.1186/1471-2393-9-S1-S5)
Supplement: Additional file 14 — Web Table 14. Component studies in Magann et al 2007 review: impact of idiopathic polyhydramnios on stillbirth and perinatal mortality. Component studies in Magann et al. 2007 review showing impact on stillbirths/perinatal mortality [file 1471-2393-9-S1-S5-S14.doc]

**Web Table 14. Component studies in Magann et al 2007 [1] review: impact of idiopathic polyhydramnios on stillbirth and perinatal mortality**

| **Source** | **Location and Type of Study** | **Intervention** | **Stillbirths / Perinatal Outcomes** |
| --- | --- | --- | --- |
| Panting-Kemp et al. 1999 [2] | USA.  Prospective case-control study. N=151 women with singleton pregnancies complicated by idiopathic polyhydramnios (amniotic fluid index >24 cm) delivered from 1996- 1998, matched with N=302 controls. | To determine whether there is any association between idiopathic polyhydramnios and adverse perinatal outcome. | PMR: NS.  Higher rates of malpresentation, macrosomia, and primary Caesarean delivery. |
| Chen et al. 2005 [3] | Taiwan. Tertiary hospital.  Retrospective review of hospital records, 1990-2001. Singleton pregnancies >20 weeks gestation. | Assessed the risks of adverse perinatal outcomes of polyhydramnios without associated fetal anomalies. | Significant increase in fetal death among polyhydramnios patients.  Polyhydramnios group also had higher rates ioflow Apgar scores, fetal death, fetal distress in labor, NICU transfer and neonatal death, |
| Biggio et al. 1999 [4] | USA (Alabama). University hospital.  Retrospective study. Computerized records analysis of N=370 women with singleton pregnancies beyond 20 weeks' gestation and hydramnios diagnosed sonographically by amniotic fluid index of 25 cm or more, largest vertical pocket of 8 cm or more, or subjective impression, compared to controls with normal AFI (N=36,426). The incidence of hydramnios was 1%. | To determine whether hydramnios is associated with an increased risk of adverse perinatal outcomes. | PMR: 49/1000 vs. 14/1000 in hydramnios vs. normal amniotic fluid groups, respectively (P<0.001).  All of the increased risk was in nondiabetic women. PMR 60/1000 vs. 0/1000 in non-diabetic vs. diabetic women with polyhydramnios (P=0.03). |
| Maymon et al. 1998 [5] | Israel.  Retrospective case study. N=60,702 singleton term gestations (>37 weeks), N=1211 with polyhydramnios (AFI of 25 cm or more, max vertical pocket at least 8 cm, or subjective assessment).. | To use logistic regression to determine if hydramnios at term gestation is an independent risk factor for poor pregnancy outcome and perinatal death. | PMR: adjusted OR=5.5 (95% CI: 3.2-9.3)  Antepartum SB: 0.6 vs. 0.2% (P<0.005). |

References

1. Magann EF, Chauhan SP, Doherty DA, Lutgendorf MA, Magann MI, Morrison JC: **A review of idiopathic hydramnios and pregnancy outcomes**. *Obstet Gynecol Surv* 2007, **62**(12):795-802.

2. Panting-Kemp A, Nguyen T, Chang E, Quillen E, Castro L: **Idiopathic polyhydramnios and perinatal outcome**. *Am J Obstet Gynecol* 1999, **181**(5 Pt 1):1079-1082.

3. Chen KC, Liou JD, Hung TH, Kuo DM, Hsu JJ, Hsieh CC, Hsieh TT: **Perinatal outcomes of polyhydramnios without associated congenital fetal anomalies after the gestational age of 20 weeks**. *Chang Gung Med J* 2005, **28**(4):222-228.

4. Biggio JR, Jr., Wenstrom KD, Dubard MB, Cliver SP: **Hydramnios prediction of adverse perinatal outcome**. *Obstet Gynecol* 1999, **94**(5 Pt 1):773-777.

5. Maymon E, Ghezzi F, Shoham-Vardi I, Franchi M, Silberstein T, Wiznitzer A, Mazor M: **Isolated hydramnios at term gestation and the occurrence of peripartum complications**. *Eur J Obstet Gynecol Reprod Biol* 1998, **77**(2):157-161.
