# Supplementary material for: Reducing stillbirths: screening and monitoring during pregnancy and labour
Source: BMC Pregnancy Childbirth. 2009 May 7;9(Suppl 1):S5. doi: 10.1186/1471-2393-9-S1-S5 (PMC2679411; doi:10.1186/1471-2393-9-S1-S5)
Supplement: Additional file 15 — Web Table 15. Component studies in Nabhan and Abdelmoula 2008 [185]meta-analysis: comparison of single deepest vertical pocket vs. AFI in predicting perinatal outcome. Component studies in Nabhan and Abdelmoula 2008 review showing impact on stillbirths/perinatal mortality [file 1471-2393-9-S1-S5-S15.doc]

**Web Table 15. Component studies in Nabhan and Abdelmoula 2008 [1] meta-analysis: comparison of single deepest vertical pocket vs. AFI in predicting perinatal outcome**

| **Source** | **Location and Type of Study** | **Intervention** | **Stillbirths / Perinatal Outcomes** |
| --- | --- | --- | --- |
| Alfirevic 1997 [2] | UK.  RCT. N = 500 women with singleton uncomplicated post-term pregnancies. | Compared the impact on perinatal mortality of amniotic fluid index (intervention) vs. single deepest vertical pocket (controls). | RR = not estimable.  [0/250 vs. 0/250 in intervention and control groups, respectively]. |
| Chauhan 2004 [3] | USA.  RCT. N = 1088 pregnant women. | Compared the impact on perinatal mortality of amniotic fluid index (intervention) vs. single deepest vertical pocket (controls). | RR = not estimable.  [0/530 vs. 0/558 in intervention and control groups, respectively]. |

References

1. Nabhan AF, Abdelmoula YA: **Amniotic fluid index versus single deepest vertical pocket as a screening test for preventing adverse pregnancy outcome**. *Cochrane Database Syst Rev* 2008(3):CD006593.

2. Alfirevic Z, Luckas M, Walkinshaw SA, McFarlane M, Curran R: **A randomised comparison between amniotic fluid index and maximum pool depth in the monitoring of post-term pregnancy**. *Br J Obstet Gynaecol* 1997, **104**(2):207-211.

3. Chauhan SP, Doherty DD, Magann EF, Cahanding F, Moreno F, Klausen JH: **Amniotic fluid index vs single deepest pocket technique during modified biophysical profile: a randomized clinical trial**. *Am J Obstet Gynecol* 2004, **191**(2):661-667; discussion 667-668.
