# Supplementary material for: Reducing stillbirths: screening and monitoring during pregnancy and labour
Source: BMC Pregnancy Childbirth. 2009 May 7;9(Suppl 1):S5. doi: 10.1186/1471-2393-9-S1-S5 (PMC2679411; doi:10.1186/1471-2393-9-S1-S5)
Supplement: Additional file 16 — Web Table 16. Component studies in Hofmeyr and Gulmezoglu 2002 [126]meta-analysis: impact of maternal hydration on amniotic fluid volume and perinatal outcomes. Component studies in Hofmeyr and Gulmezoglu 2002 review showing impact on stillbirths/perinatal mortality [file 1471-2393-9-S1-S5-S16.doc]

**Web Table 16. Component studies in Hofmeyr and Gulmezoglu 2002 meta-analysis [1]: impact of maternal hydration on amniotic fluid volume and perinatal outcomes**

| **Source** | **Location and Type of Study** | **Intervention** | **Stillbirths / Perinatal Outcomes** |
| --- | --- | --- | --- |
| Oral maternal hydration in oligohydramnios | | | |
| Doi 1998 [2, 3] | Japan.  RCT. 84 women with low amniotic fluid index and gestational age more than 35 weeks (N = 21 intervention group, N = 21 controls). | Compared the impact of oral intake of 2 litres of water over 2 hours (intervention) vs. controls. | Increase in amniotic fluid volume: WMD = 3.30 (95% CI: 2.36 – 4.24).  [Mean (SD): 3.8 (1.9) vs. 0.5 (1.1) in intervention and control groups, respectively]. |
| Kilpatrick 1991 [4, 5] | USA.  RCT. N = 40 women with oligohydramnios. | Compared the impact of women asked to drink 2 litres of water 2-4 hours before repeat ultrasound examination on the same or the next day (intervention) vs. women who were asked to drink their normal amount of fluid, and in the last 10 women an extra 100ml (controls). | Increase in amniotic fluid volume: WMD = 1.19 (95% CI: 0.44 – 1.94).  [Mean (SD): 1.5 (1.4) vs. 0.31 (0.86) in intervention and control groups, respectively]. |
| Oral maternal hydration in normal amniotic fluid volume | | | |
| Kilpatrick and Safford 1993 [6] | USA.  RCT. N = 40 women with normal amniotic fluid volume. | Compared the impact on amniotic fluid volume in women asked to drink 2 litres of water over 2 hours and re-examined 2-5 hours later (intervention) vs. group asked to drink 100ml water (controls). | Increase in amniotic fluid volume: WMD = 4.50 (95% CI: 2.92 – 6.08).  [Mean (SD): 3 (2.4) vs. -1.5 (2.7) in intervention and control groups, respectively]. |
| Intravenous/isotonic hydration in oligohydramnios | | | |
| Doi 1998 [2, 3] | Japan.  RCT. 84 women with low amniotic fluid index and gestational age more than 35 weeks (N = 21 intervention group, N = 21 controls). | Compared the impact of intravenous infusion of 2 litres of Ringer lactate over 2 hours (isotonic) (intervention) vs. controls. | Increase in amniotic fluid volume: WMD = 0.0 (95% CI: -0.67 – 0.67).  [Mean (SD): 0.5 (1.1) vs. 0.5 (1.1) in intervention and control groups, respectively]. |
| Intravenous/hypotonic hydration in oligohydramnios | | | |
| Doi 1998 [2, 3] | Japan.  RCT. 84 women with low amniotic fluid index and gestational age more than 35 weeks (N = 21 intervention group, N = 21 controls). | Assessed the impact of intravenous infusion of 2 litres of diluted Ringer lactate over 2 hours (hypotonic) (intervention) vs. controls. | Increase in amniotic fluid volume: WMD = 2.30 (95% CI: 1.36 – 3.24).  [Mean (SD): 2.8 (1.9) vs. 0.5 (1.1) in intervention and control groups, respectively]. |

References

1. Hofmeyr GJ, Gulmezoglu AM: **Maternal hydration for increasing amniotic fluid volume in oligohydramnios and normal amniotic fluid volume**. *Cochrane Database Syst Rev* 2002(1):CD000134.

2. Doi S, Osada H, Itoh K, Ikeda K, Sekiya S, Takehisa T: **Effect of maternal hydration on oligohydramnios: a comparison of three volume expansion methods.** *American Journal of Obstetrics and Gynecology;* 1998, **178**:S156.

3. Doi S, Osada H, Seki K, Sekiya S: **Effect of maternal hydration on oligohydramnios: a comparison of three volume expansion methods**. *Obstet Gynecol* 1998, **92**(4 Pt 1):525-529.

4. Kilpatrick SJ, Safford K, Pomeroy T, Hoedt L, Scheerer L, Laros RK: **Maternal hydration affects amniotic fliud index (AFI).** *American Journal of Obstetrics and Gynecology;* 1991, **164**:361.

5. Kilpatrick SJ, Safford KL, Pomeroy T, Hoedt L, Scheerer L, Laros RK: **Maternal hydration increases amniotic fluid index**. *Obstet Gynecol* 1991, **78**(6):1098-1102.

6. Kilpatrick SJ, Safford KL: **Maternal hydration increases amniotic fluid index in women with normal amniotic fluid**. *Obstet Gynecol* 1993, **81**(1):49-52.
