# Supplementary material for: Reducing stillbirths: screening and monitoring during pregnancy and labour
Source: BMC Pregnancy Childbirth. 2009 May 7;9(Suppl 1):S5. doi: 10.1186/1471-2393-9-S1-S5 (PMC2679411; doi:10.1186/1471-2393-9-S1-S5)
Supplement: Additional file 17 — Web Table 17. Component studies in Crowther 2001 meta-analysis: Impact of hospitalisation for bed rest for women with a multiple pregnancy on stillbirth and perinatal mortality. Component studies in Crowther 2001 review showing impact on stillbirths/perinatal mortality [file 1471-2393-9-S1-S5-S17.doc]

**Web Table 17. Component studies in Crowther 2001 [1] meta-analysis: Impact of hospitalisation for bed rest for women with a multiple pregnancy on stillbirth and perinatal mortality**

| **Source** | **Location and Type of Study** | **Intervention** | **Stillbirths / Perinatal Outcomes** |
| --- | --- | --- | --- |
| 1. Crowther 1989. [2] | Zimbabwe. Harare Central Hospital.  RCT. N=139 women with a twin pregnancy. | Compared the impact of the hospitalisation group (intervention) where women were admitted to the antenatal ward as soon after randomisation as convenient. Women were encouraged to rest in bed as much as possible, but ambulation was allowed. The control group was not routinely admitted and was encouraged to continue their normal activities at home. Women were selectively admitted if problems developed (pre-term labour, hypertension, pre-term pre-labour rupture of the membranes). | SBR: OR=0.99 (95% CI: 0.06-15.84) **[NS]**.  [1/140 vs. 1/138 in intervention and control groups, respectively].  PMR: OR=0.99 (95% CI: 0.14-7.07) **[NS]**.  [2/140 vs. 2/138 in intervention and control groups, respectively]. |
| 2. Crowther et al. 1990 [3] | Zimbabwe. Harare Maternity Hospital.  RCT. N=118 women with a twin pregnancy at 28-30 weeks gestation. | Compared the impact on pregnancy outcomes of the hospitalisation group (intervention) where women were admitted to the antenatal ward as soon after randomisation as convenient. Women were encouraged to rest in bed as much as possible, but ambulation was allowed. Women allocated to the control group were not routinely admitted and were encouraged to continue their normal activities at home. Women were selectively admitted if problems developed (hypertension, pre-term pre-labour rupture of the membranes, pre-term labour). | SBR: OR=0.24 (95% CI: 0.08-0.74).  [2/116 vs. 11/120 in intervention and control groups, respectively].  PMR: OR=0.37 (95% CI: 0.13-1.02) **[NS]**.  [4/112 vs. 12/120 in intervention and control groups, respectively]. |
| 3. Crowther 1991 [4] | Zimbabwe. Harare Central Hospital.  RCT. N=19 women with a triplet pregnancy at 24 weeks gestation or more. | Compared the impact of the hospitalisation group where women were asked to attend the antenatal ward as soon after recruitment as possible. Women were encouraged to rest in bed as much as possible, but ambulation was allowed. The control women were not routinely admitted and were encouraged to continue their normal activities at home. Women were selectively admitted if problems developed (hypertension, pre-term premature rupture of the membranes, pre-term labour). | SBR: OR=6.69 (95% CI: 0.13-338.79) **[NS]**.  [1/30 vs. 0/27 in intervention and control groups, respectively].  PMR: OR=0.31 (95% CI: 0.04-2.33) **[NS]**.  [1/30 vs. 3/27 in intervention and control groups, respectively]. |
| 4. Hartikainen-Sorri 1984 [5] | Finland (Oulu). Single centre.  RCT. N=73 women with a twin pregnancy. | Compared the impact of allocation to bed rest (intervention) where women were admitted to hospital for rest after the 29th week of gestation. The women in the control group were seen weekly in the specialised antenatal clinic. Selective admission if in pre-term labour, fetal distress developed or hypertension. | SBR: OR not estimable.  [0/64 vs. 0/90 in intervention and control groups, respectively].  PMR: OR=11.45 (95% CI: 1.14-115.52).  [3/64 vs. 0/90 in intervention and control groups, respectively]. |
| 5. MacLennan et al. 1990 [6] | Australia. Multicentred, tertiary care setting.  RCT. N=141 women with twin pregnancy. | Assessed the impact of allocation to bed rest (intervention) where women were admitted at 26 weeks gestation for 4 weeks. Weekend leave was allowed. Strict bed rest was not advocated. The women in the control group were not routinely admitted but seen in the clinic every two weeks. Normal home activities were encouraged. | SBR: OR=4.06 (95% CI: 0.81-20.42) **[NS]**.  [5/138 vs. 1/144 in intervention and control groups, respectively].  PMR: OR=3.61 (95% CI: 1.02-12.74).  [8/138 vs. 2/144 in intervention and control groups, respectively]. |
| 6. Saunders et al. 1985 [7] | Zimbabwe (Harare). Hospital bases study.  RCT. N=212 women with a twin pregnancy. | Assessed the effect on pregnancy outcomes of bed rest in hospital (intervention) where women were admitted for bed rest at 32 weeks gestation until the onset of labour. The control group women were not routinely admitted to hospital. Selective admission only for complications. | SBR: OR=1.69 (95% CI: 0.42-6.86) **[NS]**.  [5/210 vs. 3/214 in intervention and control groups, respectively].  PMR: OR=1.64 (95% CI: 0.54-4.94) **[NS]**.  [8/210 vs. 5/214 in intervention and control groups, respectively]. |

References

1. Crowther CA: **Hospitalisation and bed rest for multiple pregnancy**. *Cochrane Database Syst Rev* 2001(1):CD000110.

2. Crowther CA, Neilson JP, Verkuyl DA, Bannerman C, Ashurst HM: **Preterm labour in twin pregnancies: can it be prevented by hospital admission?** *Br J Obstet Gynaecol* 1989, **96**(7):850-853.

3. Crowther CA, Verkuyl DA, Neilson JP, Bannerman C, Ashurst HM: **The effects of hospitalization for rest on fetal growth, neonatal morbidity and length of gestation in twin pregnancy**. *Br J Obstet Gynaecol* 1990, **97**(10):872-877.

4. Crowther CA, Verkuyl DA, Ashworth MF, Bannerman C, Ashurst HM: **The effects of hospitalization for bed rest on duration of gestation, fetal growth and neonatal morbidity in triplet pregnancy**. *Acta Genet Med Gemellol (Roma)* 1991, **40**(1):63-68.

5. Hartikainen-Sorri AL, Jouppila P: **Is routine hospitalization needed in antenatal care of twin pregnancy?** *J Perinat Med* 1984, **12**(1):31-34.

6. MacLennan AH, Green RC, O'Shea R, Brookes C, Morris D: **Routine hospital admission in twin pregnancy between 26 and 30 weeks' gestation**. *Lancet* 1990, **335**(8684):267-269.

7. Saunders MC, Dick JS, Brown IM, McPherson K, Chalmers I: **The effects of hospital admission for bed rest on the duration of twin pregnancy: a randomised trial**. *Lancet* 1985, **2**(8459):793-795.
