# Supplementary material for: Reducing stillbirths: screening and monitoring during pregnancy and labour
Source: BMC Pregnancy Childbirth. 2009 May 7;9(Suppl 1):S5. doi: 10.1186/1471-2393-9-S1-S5 (PMC2679411; doi:10.1186/1471-2393-9-S1-S5)
Supplement: Additional file 18 — Web Table 18. Component studies in Meher et al. 2005: Impact of some rest in hospital vs. routine activity at home on stillbirth and perinatal mortality. Component studies in Meher et al. 2005 review showing impact on stillbirths/perinatal mortality [file 1471-2393-9-S1-S5-S18.doc]

**Web Table 18. Component studies in Meher et al. 2005 [1]: Impact of some rest in hospital vs. routine activity at home on stillbirth and perinatal mortality**

| **Source** | **Location and Type of Study** | **Intervention** | **Stillbirths / Perinatal Outcomes** |
| --- | --- | --- | --- |
| 1. Crowther 1992. [2] | Zimbabwe. One maternity hospital and 13 peripheral clinics.  Block randomised trial. N=218 primigravidae and multigravidae women with a singleton pregnancy at 28-38 weeks. | Compared the impact of intervention where women were admitted to hospital for rest. Allowed to move around the ward voluntarily. 4 hourly BP check and daily urinalysis. The controls had normal activity at home with no restrictions. Daily self analysis of urine for protein. Reviewed weekly for BP, weight, bloods. | SBR: RR=4.91 (95% CI: 0.24-101.10) **[NS]**.  [2/110 vs. 0/108 in intervention and control groups, respectively].  PMR: RR=1.96 (95% CI: 0.18-21.34) **[NS]**.  [2/110 vs. 1/108 in intervention and control groups, respectively].  NMR: RR=0.33 (95% CI: 0.01-7.95) **[NS]**.  [0/110 vs. 1/108 in intervention and control groups, respectively]. |

References

1. Meher S, Abalos E, Carroli G: **Bed rest with or without hospitalisation for hypertension during pregnancy**. *Cochrane Database Syst Rev* 2005(4):CD003514.

2. Crowther CA, Bouwmeester AM, Ashurst HM: **Does admission to hospital for bed rest prevent disease progression or improve fetal outcome in pregnancy complicated by non-proteinuric hypertension?** *Br J Obstet Gynaecol* 1992, **99**(1):13-17.
