# Supplementary material for: Reducing stillbirths: screening and monitoring during pregnancy and labour
Source: BMC Pregnancy Childbirth. 2009 May 7;9(Suppl 1):S5. doi: 10.1186/1471-2393-9-S1-S5 (PMC2679411; doi:10.1186/1471-2393-9-S1-S5)
Supplement: Additional file 19 — Component studies in Kroner et al. 2001: Impact of antenatal day care units on perinatal mortality. Component studies in Kroner et al. 2001 review showing impact on stillbirths/perinatal mortality [file 1471-2393-9-S1-S5-S19.doc]

**Web Table 19. Component studies in Kroner, et al. 2001 [1]: Impact of antenatal day care units on perinatal mortality**

| **Source** | **Location and Type of Study** | **Intervention** | **Stillbirths / Perinatal Outcomes** |
| --- | --- | --- | --- |
| 1. Tuffnell 1992. [2] | UK. Tertiary care setting.  RCT. N=54 women (N=30 intervention group, N=24 controls). | Compared the impact of the intervention where patients were referred to the day unit and were seen the morning after referral. Five blood-pressure readings were made; urine was checked for protein; platelet count and serum urate concentration were measured; and a cardiotocograph was done. Doppler waveform analyses were done if considered necessary by the clinician. After each visit the woman was reviewed, and further visits were arranged according to perceived clinical need; either discharged back to clinic, followed up in the day unit or admitted to hospital. Patients in the control group were managed according to the judgement and established practice of the referring clinician. This did not necessarily entail admission to the hospital. | PMR: OR not estimable.  [0/30 vs. 0/24 in intervention and control groups, respectively]. |

References

1. Kroner C, Turnbull D, Wilkinson C: **Antenatal day care units versus hospital admission for women with complicated pregnancy**. *Cochrane Database Syst Rev* 2001(4):CD001803.

2. Tuffnell DJ, Lilford RJ, Buchan PC, Prendiville VM, Tuffnell AJ, Holgate MP, Jones MD: **Randomised controlled trial of day care for hypertension in pregnancy**. *Lancet* 1992, **339**(8787):224-227.
