# Supplementary material for: Reducing stillbirths: screening and monitoring during pregnancy and labour
Source: BMC Pregnancy Childbirth. 2009 May 7;9(Suppl 1):S5. doi: 10.1186/1471-2393-9-S1-S5 (PMC2679411; doi:10.1186/1471-2393-9-S1-S5)
Supplement: Additional file 20 — Web Table 20. Component studies in Lavender et al. 2008 meta-analysis: Impact of different designs of partogram on neonatal morbidity or perinatal mortality. Component studies in Lavender et al. 2008 review showing impact on stillbirths/perinatal mortality [file 1471-2393-9-S1-S5-S20.doc]

**Web Table 20. Component studies in Lavender et al. 2008 [1] meta-analysis: Impact of different designs of partogram on neonatal morbidity or perinatal mortality**

| **Source** | **Location and Type of Study** | **Intervention** | **Stillbirths / Perinatal Outcomes** |
| --- | --- | --- | --- |
| ***2-hour action line vs, 4-hour action line*** | | | |
| Lavender 1998a [2] | UK (England).  RCT. Primigravid women (N=928) with uncomplicated pregnancies who presented in spontaneous labour at term. | Compared the impact of 2 hour action line partogram vs. 4 hour action line on pregnancy outcomes. | Serious neonatal morbidity or PMR: OR not estimable.  [0/315 vs. 0/311 in the 2 hour vs. 4 hour groups, respectively]. |
| Lavender 2006 [3] | UK (Northwest England).  RCT. Primigravid women (N=2975) with uncomplicated pregnancies, in spontaneous labour at term. | Compared the impact of 2 hour action line partogram vs. 4 hour action line on pregnancy outcomes. | Serious neonatal morbidity or PMR: OR not estimable.  [0/1490 vs. 0/1485 in the 2 hour vs. 4 hour groups, respectively]. |
| ***2-hour action line vs 3-hour action line*** | | | |
| Lavender 1998a [2] | UK (Northwest England),    RCT. Primigravid women (N=928) with uncomplicated pregnancies who presented in spontaneous labour at term. | Compared the impact on pregnancy outcome of 2 hour action line vs. 3 hour action line partogram. | Serious neonatal morbidity or PMR: OR not estimable.  [0/315 vs. 0/302 in the 2 hour vs. 3 hour group, respectively]. |
| ***3-hour action line vs 4-hour action line*** | | | |
| Lavender 1998a [2] | UK (Northwest England),  RCT. Primigravid women (N=928) with uncomplicated pregnancies who presented in spontaneous labour at term. | Compared the impact on pregnancy outcome of 3 hour action line vs. 4 hour action line partogram. | Serious neonatal morbidity or PMR: OR not estimable.  [0/302 vs. 0/311 in the 3 hour vs. 4 hour group, respectively]. |
| ***Alert line only vs alert+action lines*** | | | |
| Pattinson 2003 [4] | South Africa.  RCT. Healthy nulliparous women (N=694 who were in active spontaneous labour, at term, with a healthy singleton pregnancy and cephalic presentation. | Compared the impact on pregnancy outcome of aggressive management using a single alert line partogram, vainal exam every 2 hrs, and use of oxytocin after alert line crossed (intervention) vs. expectant (control) management with a 2-line partogram, with lines 4 hrs apart, vaginal exam every 4 hours, and oxytocin if action line crossed. | PMR: RR=7.12 (95% CI: 0.37–137.36) **[NS]**.  [3/344 vs. 0/350 in the intervention vs. control groups, respectively]. |

References

1. Lavender T, Hart A, Smyth RM: **Effect of partogram use on outcomes for women in spontaneous labour at term**. *Cochrane Database Syst Rev* 2008(4):CD005461.

2. Lavender T, Alfirevic Z, Walkinshaw S: **Partogram action line study: a randomised trial**. *Br J Obstet Gynaecol* 1998, **105**(9):976-980.

3. Lavender T, Alfirevic Z, Walkinshaw S: **Effect of different partogram action lines on birth outcomes: a randomized controlled trial**. *Obstet Gynecol* 2006, **108**(2):295-302.

4. Pattinson RC, Howarth GR, Mdluli W, Macdonald AP, Makin JD, Funk M: **Aggressive or expectant management of labour: a randomised clinical trial**. *BJOG* 2003, **110**(5):457-461.
