# Supplementary material for: Reducing stillbirths: screening and monitoring during pregnancy and labour
Source: BMC Pregnancy Childbirth. 2009 May 7;9(Suppl 1):S5. doi: 10.1186/1471-2393-9-S1-S5 (PMC2679411; doi:10.1186/1471-2393-9-S1-S5)
Supplement: Additional file 21 — Web Table 21. Component studies in Alfirevic and Devane 2006 meta-analysis: Impact of continuous cardiotocography on stillbirth and perinatal mortality. Component studies in Lavender et al. 2008 review showing impact on stillbirths/perinatal mortality [file 1471-2393-9-S1-S5-S21.doc]

**Web Table 21. Component studies in Alfirevic and Devane 2006 [1] meta-analysis: Impact of continuous cardiotocography on stillbirth and perinatal mortality**

| **Source** | **Location and Type of Study** | **Intervention** | **Stillbirths / Perinatal Outcomes** |
| --- | --- | --- | --- |
| ***Continuous CTG and FBS*** | | | |
| 1. Hansen et al. 1985 [2-4] | Copenhagen.  RCT. N=969 women (N=482 intervention group, N=487 controls). | Compared the impact of continuous CTG in conjunction with fetal blood sampling [FBS] (intervention) vs. intermittent auscultation (controls).  CTG: external or internal | PMR: RR=0.68 (95% CI: 0.11-4.04) **[NS]**.  [2/485 vs. 3/493 in intervention and control groups, respectively]. |
| 2. Haverkamp et al. 1979 [5-7] | Denver.  RCT. N=690 women (N=230 CTG without FBS group; N=229 CTG with FBS group; N=231 controls). | Compared the impact of continuous CTG with FBS (intervention) vs. intermittent auscultation (controls).  CTG: external until internal feasible. | PMR: RR=1.52 (95% CI: 0.06-37.01) **[NS]**.  [1/230 vs. 0/116 in intervention and control groups, respectively]. |
| 3. MacDonald et al. 1985 [8-12] | Dublin.  RCT. N=12,964 women (N=6474 intervention group, N=6490 controls). | Compared the impact of continuous CTG in conjunction with FBS (intervention) vs. intermittent auscultation (controls).  CTG: internal. | PMR: RR=1.00 (95% CI: 0.48-2.10) **[NS]**.  [14/6530 vs. 14/6554 in intervention and control groups, respectively]. |
| 4. Renou et al. 1976 [13, 14] | Australia.  RCT. N=350 women (N=175 intervention group, N=175 controls). | Compared the impact of continuous CTG with FBS (intervention) vs. intermittent auscultation (controls).  CTG: external | PMR: RR=1.00 (95% CI: 0.06-15.86) **[NS]**.  [1/175 vs. 1/175 in intervention and control groups, respectively]. |
| 5. Azhar et al.1989 [15] | Pakistan.  RCT. N=200 women (N=100 intervention group, N=100 controls). | Compared the impact of continuous CTG with FBS (intervention) vs. intermittent auscultation (controls).  CTG: external. | PMR: RR=0.80 (95% CI: 0.22-2.89) **[NS]**.  [4/100 vs. 5/100 in intervention and control groups, respectively]. |
| 6. Luthy et al. 1987 [16-20] | USA.  RCT. N=246 women (N=122 intervention group, N=124 controls) with babies weighing > 1750 g. | Compared the impact of continuous CTG with FBS (intervention) vs. intermittent auscultation (controls).  CTG: external until rupture of membranes then internal. | PMR: RR=0.96 (95% CI: 0.52-1.77) **[NS]**.  [17/122 vs. 18/124 in intervention and control groups, respectively]. |
| ***Continuous CTG only*** | | | |
| 7. Vintzileos et al. 1993 [21-24] | Athens.  Quasi-RCT. N=1428 women (N=746 intervention group, N=682 controls). | Compared the impact of continuous CTG without FBS (intervention) vs. intermittent auscultation (controls).  CTG: external unless trace was poor, then internal | PMR: RR=0.20 (95% CI: 0.04-0.94) **[NS]**.  [2/746 vs. 9/682 in intervention and control groups, respectively]. |
| 8. Leveno et al. 1986 [25, 26] | USA.  RCT. N=14,618 women with low-risk pregnancies (N=7288 intervention group, N=7330 controls). | Compared the impact of continuous CTG monitoring (intervention) vs. intermittent auscultation (controls).  CTG: no information on external or internal. | PMR: RR=0.80 (95% CI: 0.22-3.00) **[NS]**.  [4/7288 vs. 5/7330 in intervention and control groups, respectively]. |
| 9. Haverkamp et al. 1976 [27] | Denver.  RCT. N=483 women (N=242 intervention group, N=241 controls). | Compared the impact of continuous CTG without FBS (intervention) vs. intermittent auscultation (controls).  CTG: internal. | PMR: RR=1.99 (95% CI: 0.18-21.82) **[NS]**.  [2/242 vs. 1/241 in intervention and control groups, respectively]. |
| 10. Haverkamp et al. 1979 [5-7] | Denver.  RCT. N=690 women (N=230 CTG without FBS group; N=229 CTG with FBS group; N=231 controls). | Compared the impact of continuous CTG without FBS (intervention) vs. intermittent auscultation (controls).  CTG: external until internal feasible. | PMR: RR=2.50 (95% CI: 0.12-51.65) **[NS]**.  [2/233 vs. 0/116 in intervention and control groups, respectively]. |
| 11. Wood et al. 1981 [28] | Australia.  RCT. N=989 women (N=445 intervention group, N=482 controls). | Compared the impact of continuous CTG without FBS (intervention) vs. intermittent auscultation (controls).  CTG: external until membranes ruptured then internal. | PMR: RR=3.25 (95% CI: 0.13-79.55) **[NS]**.  [1/445 vs. 0/482 in intervention and control groups, respectively]. |
| 12. Kelso et al. 1978 [29] | Sheffield.  RCT. N=504 women (N=253 intervention group, N=251 controls). | Compared the impact of continuous internal CTG without FBS (intervention) vs. intermittent auscultation (controls).  CTG: internal. | PMR: RR=0.33 (95% CI: 0.01-8.08) **[NS]**.  [0/253 vs. 1/251 in intervention and control groups, respectively]. |

References

1. Alfirevic Z, Devane D, Gyte GM: **Continuous cardiotocography (CTG) as a form of electronic fetal monitoring (EFM) for fetal assessment during labour**. *Cochrane Database Syst Rev* 2006, **3**:CD006066.

2. Hansen PK, Smith SF, Nim J, Neldam S, Osler M: **Maternal attitudes to fetal monitoring**. *Eur J Obstet Gynecol Reprod Biol* 1985, **20**(1):43-51.

3. Neldam S, Osler M, Hansen PK, Nim J, Smith SF, Hertel J: **[Monitoring labor with cardiotocography and stethoscopic examination in normal and risk deliveries]**. *Ugeskr Laeger* 1985, **147**(37):2901-2907.

4. Neldam S, Osler M, Hansen PK, Nim J, Smith SF, Hertel J: **Intrapartum fetal heart rate monitoring in a combined low- and high-risk population: a controlled clinical trial**. *Eur J Obstet Gynecol Reprod Biol* 1986, **23**(1-2):1-11.

5. Haverkamp AD, Orleans M, Langendoerfer S, McFee J, Murphy J, Thompson HE: **A controlled trial of the differential effects of intrapartum fetal monitoring**. *Am J Obstet Gynecol* 1979, **134**(4):399-412.

6. Koszalka MF, Jr., Haverkamp AD, Orleans M, Murphy J: **The effects of internal electronic fetal heart rate monitoring on maternal and infant infections in high-risk pregnancies**. *J Reprod Med* 1982, **27**(10):661-665.

7. Langendoerfer S, Haverkamp AD, Murphy J, Nowick KD, Orleans M, Pacosa F, van Doorninck W: **Pediatric follow-up of a randomized controlled trial of intrapartum fetal monitoring techniques**. *J Pediatr* 1980, **97**(1):103-107.

8. Boylan P, MacDonald D, Grant AM, Pereira M, Chalmers I: **The Dublin randomised controlled trial of intrapartum fetal heart rate monitoring**. In: *Fetal heart rate monitoring.* Edited by W K. Berlin: Springer Verlag; 1985: 231-233.

9. MacDonald D, Grant A, Sheridan-Pereira M, Boylan P, Chalmers I: **The Dublin randomized controlled trial of intrapartum fetal heart rate monitoring**. *Am J Obstet Gynecol* 1985, **152**(5):524-539.

10. Ellison PH, Foster M, Sheridan-Pereira M, MacDonald D: **Electronic fetal heart monitoring, auscultation, and neonatal outcome**. *Am J Obstet Gynecol* 1991, **164**(5 Pt 1):1281-1289.

11. Garcia J, Corry M, MacDonald D, Elbourne D, Grant A: **Mothers' views of continuous electronic fetal heart monitoring and intermittent auscultation in a randomized controlled trial**. *Birth* 1985, **12**(2):79-86.

12. Grant A, O'Brien N, Joy MT, Hennessy E, MacDonald D: **Cerebral palsy among children born during the Dublin randomised trial of intrapartum monitoring**. *Lancet* 1989, **2**(8674):1233-1236.

13. Renou P, Chang A, Anderson I, Wood C: **Controlled trial of fetal intensive care**. *Am J Obstet Gynecol* 1976, **126**(4):470-476.

14. Wood C, Renou P: **Fetal heart rate monitoring, Chapter 23**. In: *Fetal physiology and medicine.* Edited by Nathanielsz PW BR. London: Saunders; 1976: 471-473.

15. Azhar NA, Neilson JP: **Randomised trial of electronic intrapartum fetal heart rate monitoring with fetal blood sampling versus intermittent auscultation in a developing country. Personal communication**. In*.*, Personal communication edn; 2001.

16. Killien MG, Shy K: **A randomized trial of electronic fetal monitoring in preterm labor: mothers' views**. *Birth* 1989, **16**(1):7-12.

17. Larson EB, van Belle G, Shy KK, Luthy DA, Strickland D, Hughes JP: **Fetal monitoring and predictions by clinicians: observations during a randomized clinical trial in very low birth weight infants**. *Obstet Gynecol* 1989, **74**(4):584-589.

18. Luthy DA, Shy KK, Van Belle G, Larson EB, Hughes J, Benedetti TJ, al e: **A randomized trial of electronic fetal heart rate monitoring in infants of low birth weight**. In: *Proceedings of 6th Annual Meeting of the Society of Perinatal Obstetricians: 1986 January 30-February 1; San Antonio, Texas, USA*; 1986 January 30-February 1.

19. Luthy DA, Shy KK, van Belle G, Larson EB, Hughes JP, Benedetti TJ, Brown ZA, Effer S, King JF, Stenchever MA: **A randomized trial of electronic fetal monitoring in preterm labor**. *Obstet Gynecol* 1987, **69**(5):687-695.

20. Shy KK, Luthy DA, Bennett FC, Whitfield M, Larson EB, van Belle G, Hughes JP, Wilson JA, Stenchever MA: **Effects of electronic fetal-heart-rate monitoring, as compared with periodic auscultation, on the neurologic development of premature infants**. *N Engl J Med* 1990, **322**(9):588-593.

21. Vintzileos AM, Antsaklis AJ, Varvarigos I, Karaiskakis P, Gazis I, Pappas C, al e: **A prospective randomized trial of intrapartum electronic fetal heart rate monitoring vs intermittent auscultation**. *American Journal of Obstetrics and Gynecology;* 1993, **168**:343.

22. Vintzileos AM, Antsaklis A, Varvarigos I, Papas C, Sofatzis I, Montgomery JT: **A randomized trial of intrapartum electronic fetal heart rate monitoring versus intermittent auscultation**. *Obstet Gynecol* 1993, **81**(6):899-907.

23. Vintzileos A, Nochimson D, Guzman E, Knuppel R: **Comparison of intrapartum electronic fetal heart rate monitoring vs intermittent auscultation in detecting fetal acidemia at birth**. *American Journal of Obstetrics and Gynecology;* 1995, **172**:367.

24. Vintzileos AM, Nochimson DJ, Antsaklis A, Varvarigos I, Guzman ER, Knuppel RA: **Comparison of intrapartum electronic fetal heart rate monitoring versus intermittent auscultation in detecting fetal acidemia at birth**. *Am J Obstet Gynecol* 1995, **173**(4):1021-1024.

25. Leveno KJ, Cunningham FG, Nelson S, Roark ML, Williams ML, Guzick DS, al e: **Selected versus universal electronic fetal monitoring: a randomized study of 31,352 women.** In: *Proceedings of 6th Annual Meeting fo the Society of Perinatal Obstetricians: 1986 January 30-February 1.; San Antonio, Texas, USA.*; 1986 January 30-February 1.

26. Leveno KJ, Cunningham FG, Nelson S, Roark M, Williams ML, Guzick D, Dowling S, Rosenfeld CR, Buckley A: **A prospective comparison of selective and universal electronic fetal monitoring in 34,995 pregnancies**. *N Engl J Med* 1986, **315**(10):615-619.

27. Haverkamp AD, Thompson HE, McFee JG, Cetrulo C: **The evaluation of continuous fetal heart rate monitoring in high-risk pregnancy**. *Am J Obstet Gynecol* 1976, **125**(3):310-320.

28. Wood C, Renou P, Oats J, Farrell E, Beischer N, Anderson I: **A controlled trial of fetal heart rate monitoring in a low-risk obstetric population**. *Am J Obstet Gynecol* 1981, **141**(5):527-534.

29. Kelso IM, Parsons RJ, Lawrence GF, Arora SS, Edmonds DK, Cooke ID: **An assessment of continuous fetal heart rate monitoring in labor. A randomized trial**. *Am J Obstet Gynecol* 1978, **131**(5):526-532.
