# Supplementary material for: Reducing stillbirths: screening and monitoring during pregnancy and labour
Source: BMC Pregnancy Childbirth. 2009 May 7;9(Suppl 1):S5. doi: 10.1186/1471-2393-9-S1-S5 (PMC2679411; doi:10.1186/1471-2393-9-S1-S5)
Supplement: Additional file 22 — Web Table 22. Component studies in Neilson 2006 meta-analysis: Impact of fetal electrocardiogram (ECG) on perinatal mortality. Component studies in Neilson meta-analysis showing impact on stillbirths/perinatal mortality [file 1471-2393-9-S1-S5-S22.doc]

**Web Table 22. Component studies in Neilson 2006 meta-analysis [1] : Impact of fetal electrocardiogram (ECG) on perinatal mortality.**

| **Source** | **Location and Type of Study** | **Intervention** | **Stillbirths / Perinatal Outcomes** |
| --- | --- | --- | --- |
| ***ST analysis*** | | | |
| 1. Amer-Wahlin et al. 2001 [2] | Sweden. 3 centres (Lund, Malmo, Gothenburg).  RCT. N=4966 women in labour between 1998 and 2000 (N=2519 intervention group, N=2477 controls). | Compared the impact on PMR of CTG plus ST analysis of fetal ECG (intervention) vs. CTG alone (controls). | PMR: RR=1.46 (95% CI: 0.24-8.71) **[NS]**.  [3/2519 vs. 2/2447 in intervention and control groups, respectively]. |
| 2. Ojala et al. 2006 [3] | Finland. Labour ward in tertiary-level university hospital.  RCT. N=1483 women between 2003-4 (N=733 intervention group, N=739 controls). | Compared the impact of CTG plus ECG waveform analysis (intervention) vs. CTG (controls). Fetal scalp sampling for pH estimation an option in either group. | PMR: RR not estimable.  [0/733 vs. 0/739 in intervention and control groups, respectively]. |
| 3. Westgate et al. 1993 [4-7] | England (Plymouth). District general hospital.  RCT. N=2434 high-risk labours more than 34 weeks’ gestation with no gross fetal abnormality (N=1219 intervention group, N=1215 controls). | Compared the impact of CTG plus ST analysis (intervention) vs. CTG alone (controls). | PMR: RR=4.98 (95% CI: 0.24-103.70) **[NS]**.  [2/1219 vs. 0/1215 in intervention and control groups, respectively]. |
| ***PR (time-interval) analysis*** | | | |
| 4. Strachan et al, for the FECG Study Group. 2000 [8] | UK (Nottingham and Dundee), China (Hong Kong), Netherlands (Amsterdam) and Singapore. Five centres.  RCT. N=957 women in labour (N=482 intervention, N=475 controls). | Compared the impact of CTG plus fetal ECG (intervention) vs. CTG alone (controls). | PMR: RR=2.96 (95% CI: 0.12-72.39) **[NS]**.  [1/482 vs. 0/475 in intervention and control groups, respectively]. |

References

1. Neilson JP: **Fetal electrocardiogram (ECG) for fetal monitoring during labour**. *Cochrane Database Syst Rev* 2006, **3**:CD000116.

2. Amer-Wahlin I, Hellsten C, Noren H, Hagberg H, Herbst A, Kjellmer I, Lilja H, Lindoff C, Mansson M, Martensson L *et al*: **Cardiotocography only versus cardiotocography plus ST analysis of fetal electrocardiogram for intrapartum fetal monitoring: a Swedish randomised controlled trial**. *Lancet* 2001, **358**(9281):534-538.

3. Ojala K, Vaarasmaki M, Makikallio K, Valkama M, Tekay A: **A comparison of intrapartum automated fetal electrocardiography and conventional cardiotocography--a randomised controlled study**. *BJOG* 2006, **113**(4):419-423.

4. Westgate J, Harris M, Curnow J, Greene K: **Plymouth randomised controlled trial of 2400 cases - ST waveform plus CTG vs CTG alone for intrapartum monitoring**. In: *Proceedings of 26th British Congress of Obstetrics and Gynaecology: 1992; Manchester, UK*; 1992.

5. Westgate J, Harris M, Curnow JSH, Greene KR: **Plymouth randomized trial of CTG vs ST waveform analysis plus CTG for intrapartum monitoring: 2400 cases**. *Journal of Perinatal Medicine;* 1992, **20**:268.

6. Westgate J, Harris M, Curnow JS, Greene KR: **Randomised trial of cardiotocography alone or with ST waveform analysis for intrapartum monitoring**. *Lancet* 1992, **340**(8813):194-198.

7. Westgate J, Harris M, Curnow JS, Greene KR: **Plymouth randomized trial of cardiotocogram only versus ST waveform plus cardiotocogram for intrapartum monitoring in 2400 cases**. *Am J Obstet Gynecol* 1993, **169**(5):1151-1160.

8. Strachan BK, van Wijngaarden WJ, Sahota D, Chang A, James DK: **Cardiotocography only versus cardiotocography plus PR-interval analysis in intrapartum surveillance: a randomised, multicentre trial. FECG Study Group**. *Lancet* 2000, **355**(9202):456-459.
