# Supplementary material for: Reducing stillbirths: screening and monitoring during pregnancy and labour
Source: BMC Pregnancy Childbirth. 2009 May 7;9(Suppl 1):S5. doi: 10.1186/1471-2393-9-S1-S5 (PMC2679411; doi:10.1186/1471-2393-9-S1-S5)
Supplement: Additional file 23 — Web Table 23. Component studies in East et al. 2007 meta-analysis: Impact of fetal pulse oximetry on fetal/neonatal death. Component studies in East et al. 2007 meta-analysis showing impact on stillbirths/perinatal mortality [file 1471-2393-9-S1-S5-S23.doc]

**Web Table 23. Component studies in East et al. 2007 [1] meta-analysis: Impact of fetal pulse oximetry on fetal/neonatal death**

| **Source** | **Location and Type of Study** | **Intervention** | **Stillbirths / Perinatal Outcomes** |
| --- | --- | --- | --- |
| ***Gestation from 36 weeks, fetal blood sampling (FBS) not required prior to study entry*** | | | |
| 1. East et al. on behalf of the FOREMOST Study Group. 2006 [2] | Australia. Multicentred (4 maternity hospitals).  RCT. N=601 women in labour. | Compared the impact on fetal outcome of the intervention where CTG plus fetal pulse oximetry was peformed. The control group had fetal heart rate monitoring (CTG) (doppler/fetal scalp electrode) only. | Fetal/neonatal death: RR=0.32 (95% CI: 0.01-7.88) **[NS]**.  [0/305 vs. 1/295 in intervention and control groups, respectively]. |
| 2. Garite et al. 2000 [3] | USA.  RCT. N=1189 women in labour. | Compared the impact of CTG plus fetal pulse oximetry (intervention) vs. control group with fetal heart rate monitoring (CTG) (doppler/fetal scalp electrode) only. | Fetal/neonatal death: RR=1.30 (95% CI: 0.22-7.75) **[NS]**.  [3/637 vs. 2/552 in intervention and control groups, respectively]. |
| ***Gestation from 36 weeks, FBS prior to study entry*** | | | |
| 3. Kuhnert and Schmidt 2004 [4] | Germany. Single-centre.  RCT. N=146 women in labour. | Compared the impact of CTG plus FBS plus pulse oximetry (intervention) vs. fetal heart rate monitoring by CTG only and FBS. | Fetal/neonatal death: RR not estimable.  [0/73 in both the groups]. |
| ***Gestation from 36 weeks, nonreassuring fetal status not required prior to study entry*** | | | |
| 4. Bloom et al. 2006 [5] | U.S.A.  RCT. N=5341 nulliparous women who were at term and in early labour. | Compared the impact of open (intervention) group where the fetal pulse oximetry values were displayed vs. masked group (control) where fetal pulse oximetry values not displayed, only recorded on computer.  Both groups: standard fetal heart rate monitoring; labour management at the clinician's discretion. | Fetal/neonatal death: RR=0.34 (95% CI: 0.01-8.44) **[NS]**.  [0/2629 vs. 1/2712 in intervention and control groups, respectively]. |

1. East CE, Chan FY, Colditz PB, Begg LM: **Fetal pulse oximetry for fetal assessment in labour**. *Cochrane Database Syst Rev* 2007(2):CD004075.

2. East CE, Brennecke SP, King JF, Chan FY, Colditz PB: **The effect of intrapartum fetal pulse oximetry, in the presence of a nonreassuring fetal heart rate pattern, on operative delivery rates: a multicenter, randomized, controlled trial (the FOREMOST trial)**. *Am J Obstet Gynecol* 2006, **194**(3):606 e601-616.

3. Garite TJ, Dildy GA, McNamara H, Nageotte MP, Boehm FH, Dellinger EH, Knuppel RA, Porreco RP, Miller HS, Sunderji S *et al*: **A multicenter controlled trial of fetal pulse oximetry in the intrapartum management of nonreassuring fetal heart rate patterns**. *Am J Obstet Gynecol* 2000, **183**(5):1049-1058.

4. Kuhnert M, Schmidt S: **Intrapartum management of nonreassuring fetal heart rate patterns: a randomized controlled trial of fetal pulse oximetry**. *Am J Obstet Gynecol* 2004, **191**(6):1989-1995.

5. Bloom SL, Spong CY, Thom E, Varner MW, Rouse DJ, Weininger S, Ramin SM, Caritis SN, Peaceman A, Sorokin Y *et al*: **Fetal pulse oximetry and cesarean delivery**. *N Engl J Med* 2006, **355**(21):2195-2202.
